# Global Stability for Charged Scalar Fields in an Asymptotically Flat Metric in Harmonic Gauge

**DOI:** 10.1007/s00023-023-01341-x

**Published:** 2023-07-24

**Authors:** Christopher Kauffman

**Affiliations:** Westfälische Wilhelms-Universität Münster: Westfalische Wilhelms-Universitat Munster, Münster, Germany

## Abstract

We prove global stability for the charged scalar field system on a background spacetime, which is close to $1+3$-dimensional Minkowski space and whose outward light cones converge to those for the Schwarzs-child metric at null infinity. The key technique to this proof is the use of a modified null frame, depending only on the mass *M* of the metric, which captures the asymptotic behavior of the metric at future null infinity. Our results are analogous to results obtained in Minkowski space by Lindblad and Sterbenz in (IMRP Int Math Res Pap 2006:52976, 2006) up to a change in coordinates, and will in the sequel be used to prove the full structure of the Einstein–charge scalar field system in these modified harmonic coordinates.

## Introduction

### The Maxwell–Klein–Gordon System

The Maxwell–Klein–Gordon system, also called the charge-scalar field system, relates the evolution of the curvature ${\textbf{F}}$ of a line bundle *V* with the evolution of a section $\varvec{\phi }$ of that bundle. This system may be thought of physically as a simplified model describing the evolution of charged matter, or mathematically as a toy model for the full Yang–Mills–Higgs system, using the abelian gauge group *U*(1) rather than the nonabelian groups of Yang–Mills theory.

Given a background $1+3$-dimensional Lorentzian manifold $({\mathcal {M}}, g)$, we take $V = {\mathcal {M}}\times {\mathbb {C}}$ to be the trivial complex line bundle over ${\mathcal {M}}$ with structure group $U(1) = \{e^{i\chi }\}$, along with a Hermitian inner product $\langle \cdot , \cdot \rangle _V$, under which the structure group is unitary. From this, we take a compatible connection *D* so that, taking $\phi$ and $\psi$ to be sections of *V*, we have

1.1 $$\begin{aligned} X(\langle \phi , \psi \rangle _V) = \langle D_X\phi , \psi \rangle _V + \langle \phi , D_X\psi \rangle _V. \end{aligned}$$

We may extend *D* to complex-valued (*k*, *l*)-tensors *T*, including scalar functions, using the Leibniz rule

1.2 $$\begin{aligned} D_X(\phi \otimes T) = (D_X\phi )\otimes T + \phi \otimes \nabla _X T, \end{aligned}$$

where $\nabla$ is the Levi–Civita connection on ${\mathcal {M}}$.

Given this, we choose a global section ${\textbf{1}}_V$ satisfying

1.3 $$\begin{aligned} \langle {\textbf{1}}_V, {\textbf{1}}_V\rangle _V \equiv 1. \end{aligned}$$

We define the potential 1-form ${\textbf{A}}$ on ${\mathcal {M}}$ associated with ${\textbf{1}}_V$ satisfying

1.4 $$\begin{aligned} D_X({\textbf{1}}_V) = i{\textbf{A}}(X){\textbf{1}}_V. \end{aligned}$$

It follows from Eq. (1.1) that ${\textbf{A}}$ has real coefficients.

Given a section $\psi$ of *V*, we associate a complex scalar function $\varvec{\psi }$ on ${\mathcal {M}}$ using

1.5 $$\begin{aligned} \psi = \varvec{\psi }\otimes {\textbf{1}}_V, \end{aligned}$$

so that

1.6 $$\begin{aligned} D_X\psi := D_X(\varvec{\psi }\otimes {\textbf{1}}_V) = (\nabla _X\varvec{\psi })\otimes {\textbf{1}}_V + \varvec{\psi }\otimes i{\textbf{A}}(X){\textbf{1}}_V. \end{aligned}$$

Alternatively,

1.7 $$\begin{aligned} D_X\varvec{\psi }= \nabla _X\varvec{\psi }+ i{\textbf{A}}(X)\varvec{\psi }. \end{aligned}$$

We extend this to tensors in the same way, associating complex-valued tensors $T'$ with elements of the product space so that (1.7) holds:

1.8 $$\begin{aligned} T' = T\otimes {\textbf{1}}_V. \end{aligned}$$

We will use this notation to suppress the use of ${\textbf{1}}_V$, and formulate the system for a scalar function $\varvec{\phi }$. We remark that the (pointwise-defined) quantities

1.9 $$\begin{aligned} \varvec{\psi }{\overline{\varvec{\psi }}} = \varvec{\psi }{\overline{\varvec{\psi }}}\langle 1_V, 1_V\rangle _V = \langle \psi , \psi \rangle _V, \quad |\varvec{\psi }| = \langle \psi , \psi \rangle ^{1/2} \end{aligned}$$

do not depend on our choice of ${\textbf{1}}_V$. Given a real scalar function *f*, the identity (1.2) implies the Leibniz rule

1.10 $$\begin{aligned} D_X(f\varvec{\psi }) = f D_X\varvec{\psi }+ X(f)\varvec{\psi }. \end{aligned}$$

We may define the curvature 2-form ${\textbf{F}}$ associated with the connection *D* using the formula

1.11 $$\begin{aligned} i{\textbf{F}}(X, Y) \cdot \varvec{\psi }= D_XD_Y\varvec{\psi }- D_YD_X\varvec{\psi }- D_{[X, Y]}\varvec{\psi }, \end{aligned}$$

or equivalently, as the exterior derivative

1.12 $$\begin{aligned} {\textbf{F}}= d{\textbf{A}}. \end{aligned}$$

Given a complex scalar function $\varvec{\phi }$ and a potential 1-form ${\textbf{A}}$ with exterior derivative ${\textbf{F}}$, we say $({\textbf{F}}, \varvec{\phi })$ is a solution of the Maxwell–Klein–Gordon system if it satisfies the Euler–Lagrange equations for the Lagrangian

1.13 $$\begin{aligned} \int _{{\mathcal {M}}} \frac{1}{2} \langle D_\alpha \varvec{\phi }, D^\alpha \varvec{\phi }\rangle + \frac{1}{4} {\textbf{F}}_{\alpha \beta } {\textbf{F}}^{\alpha \beta }, \end{aligned}$$

with indices raised with respect to *g*. Equivalently,

1.14a $$\begin{aligned} \Box _g^{{\mathbb {C}}}\varvec{\phi }&= D^\alpha D_\alpha \varvec{\phi }= 0, \end{aligned}$$

1.14b $$\begin{aligned} \nabla ^\beta {\textbf{F}}_{\alpha \beta }&= {\textbf{J}}[\varvec{\phi }]_\alpha = {\mathfrak {I}}\left( \varvec{\phi }\overline{D_\alpha \varvec{\phi }}\right) , \end{aligned}$$

1.14c $$\begin{aligned} \nabla ^\beta ( \star {\textbf{F}})_{\alpha \beta }&= 0. \end{aligned}$$

Here and in what follows, ${\mathfrak {R}}, {\mathfrak {I}}$ denote the projections onto the real and imaginary parts. We call $\Box _g^{{\mathbb {C}}}$ the complex covariant wave operator, and ${\textbf{J}}^\alpha$ is the current vector. The coupling between $\varvec{\phi }$ and ${\textbf{F}}$ arises from the right-hand side of (1.14b) along with the commutator

1.15 $$\begin{aligned}{}[D_\alpha , D_\beta ]\psi = i{\textbf{F}}_{\alpha \beta }\psi . \end{aligned}$$

The energy–momentum tensor of this system is

1.16 $$\begin{aligned} T_{\alpha \beta }= & {} T[\varvec{\phi }, {\textbf{F}}]_{\alpha \beta } = {\mathfrak {R}}\left( D_\alpha \varvec{\phi }\overline{D_\beta \varvec{\phi }} - \frac{1}{2}g_{\alpha \beta }D_\gamma \varvec{\phi }\overline{D^\gamma \varvec{\phi }}\right) \nonumber \\{} & {} + {\textbf{F}}_{\alpha \gamma }{\textbf{F}}_\beta ^{\,\,\gamma } - \frac{1}{4}g_{\alpha \beta }{\textbf{F}}_{\gamma \delta }{\textbf{F}}^{\gamma \delta }. \end{aligned}$$

We may also separate it into its scalar and field portions, respectively

1.17a $$\begin{aligned} T[\varvec{\phi }]_{\alpha \beta }&= {\mathfrak {R}}\left( D_\alpha \varvec{\phi }\overline{D_\beta \varvec{\phi }} - \frac{1}{2} g_{\alpha \beta }D_\gamma \varvec{\phi }\overline{D^\gamma \varvec{\phi }}\right) , \end{aligned}$$

1.17b $$\begin{aligned} T[{\textbf{F}}]_{\alpha \beta }&= {\textbf{F}}_{\alpha \gamma }{\textbf{F}}_\beta ^{\,\,\gamma } - \frac{1}{4}g_{\alpha \beta }{\textbf{F}}_{\gamma \delta }{\textbf{F}}^{\gamma \delta }. \end{aligned}$$

These satisfy the identities

1.18 $$\begin{aligned} \nabla ^\beta T[\varvec{\phi }]_{\alpha \beta } = {\textbf{F}}_{\alpha \gamma }{\textbf{J}}^\gamma , \quad \nabla ^\beta T[{\textbf{F}}]_{\alpha \beta } = -{\textbf{F}}_{\alpha \gamma }{\textbf{J}}^\gamma , \quad g^{\alpha \beta }T[{\textbf{F}}]_{\alpha \beta } = 0. \end{aligned}$$

The scalar divergence identity follows from (1.15) along with Eq. (1.1), and the corresponding vector divergence identity follows from antisymmetry along with

1.19 $$\begin{aligned} d{\textbf{F}}= 0. \end{aligned}$$

In the sequel [11], we use these stability results to couple the system (1.14) to Einstein’s field equations,

1.20 $$\begin{aligned} R_{\mu \nu } = T_{\mu \nu } - \tfrac{1}{2}g_{\mu \nu }g^{\alpha \beta }T_{\alpha \beta }. \end{aligned}$$

Given the system (1.14) along with suitable initial conditions on ${\textbf{F}}$ and $\varvec{\phi }$, one has some freedom in the choice of ${\textbf{1}}_V$, and correspondingly of the potential ${\textbf{A}}$, so that (1.14) is invariant. For a given real function $\psi$, if $({\textbf{F}}, \varvec{\phi })$ solves (1.14) with potential ${\textbf{A}}$, then $({\textbf{F}}, e^{-i\psi }\varvec{\phi })$ solves (1.14) with potential ${\textbf{A}}+ d\psi$. Fixing a gauge has historically aided in proving local and global existence results, even for the more general Yang–Mills–Higgs equations. Eardley and Moncrief [7] proved local and global existence using the temporal gauge ${\textbf{A}}_0 = 0$, and subsequently, Klainerman and Machedon [12, 13], extended their result using elliptic estimates arising from the Coulomb gauge $\nabla _x\cdot {\textbf{A}}= 0$. However, these results did not rule out polynomially growing energy, so their utility in establishing decay was limited. In [4], we obtain asymptotics for ${\textbf{A}}$ in the Lorenz gauge $\nabla _{t,x}\cdot {\textbf{A}}= 0$, which follows from a weak null structure similar to that for Einstein’s equations. Existence of scattering states in this gauge was later shown by He in [9]. However, the proof here is gauge-invariant.

Improved estimates for small initial data were later proven by Shu [25, 26]. A more rigorous proof of this was given by Lindblad and Sterbenz [18], and later Bieri, Miao, and Shashahani [2], who built on the results of Shu to prove global stability and decay for small initial data in a gauge-invariant way. Decay results for large Maxwell data were established by Yang [30, 31].

In [32], Yang and Yu extended the result of Yang to find decay bounds for solutions with large initial data. However, the decay required on ${\textbf{F}}$ in this paper is in a sense unphysical, in that after subtracting off a 2-form which picked up the charge, the remainder must decay rapidly enough that the dipole and quadrupole moments must vanish. Wei, Yang, and Yu, in [29], were later able to relax the decay assumptions of the initial data. Notably, in this paper they also extended partial decay results to the full Yang–Mills–Higgs system for charge-free fields. The extension to fields with nonzero charge remains an open problem of some interest, as in general Yang–Mills fields, asymptotics for fields with nonzero charge are not well understood.

We remark that the Maxwell–Klein–Gordon system in $1+4$ dimensions is of separate interest, as here the system becomes energy critical, see, for instance, [21] and its sequels.

Substantial work has also been done in the massive case, in which (1.14a) is replaced with an appropriate Klein–Gordon-type equation. Notably by Psarrelli [24] in the massive case, Analogous decay results for small initial data were given by Klainerman, Wang, and Yang in [14], and extended to the case of a large Maxwell field in [8].

Our work builds on the techniques in [18]; however, one quickly runs into the problem that, if treating *g* strictly as a perturbation of a background Minkowski metric in harmonic coordinates, certain conformal Morawetz- and Strichartz-type estimates fail to be coercive. The stability of components of *g* in these coordinates was nevertheless shown by Lindblad and Rodnianski [17] and was extended to systems with electromagnetic components by Loizelet [20] and later by Speck [27]. However, the techniques used therein give energy which grows like $t^\delta$, which would pose a substantial obstacle, since, for example, the right-hand side of (1.14b) is expected to have worse decay in $t-r$ than corresponding nonlinearities in Einstein’s vacuum equations. This energy growth comes from two sources. The first, which does not appear in the Maxwell–Klein–Gordon system, comes from the nature of the quadratic nonlinearities in the (harmonic gauge) Einstein vacuum equations. The second, with which we must content here, comes from the weak control on the null structure; specifically, that for $L = \partial _t + \partial _r$, the component *g*(*L*, *L*) decays like $t^{-1}$ near the light cone.

A geometric approach to this system, as in the landmark work of Christodoulou and Klainerman [5] and its extension to the Maxwell system by Zipser [33], would be to control this energy growth with a geometric null frame, at the cost of greatly increasing the complexity of the calculations required. We instead show that a modification of the null frame depending only on the ADM mass *M*, combined with refined estimates on the metric in harmonic coordinates found by Lindblad [16], serves as a sufficient approximation to the geometric null frame, while maintaining the relative simplicity of harmonic coordinate methods.

### The Background Spacetime

We consider spacetimes $({\mathcal {M}}, g)$ close to the $1+3$-dimensional Minkowski spacetime on a time interval [0, *T*], in the sense that, on this interval, *g* satisfies certain $L^2$ and $L^\infty$ estimates consistent with small-data solutions to Einstein’s Vacuum Equations in harmonic gauge. We will use both the Cartesian coordinates $(x^0, x^1, x^2, x^3)$, writing also $t=x^0$. We will also write points in ${\mathcal {M}}$ using the polar coordinate notation

1.21 $$\begin{aligned} (t, r, \omega ) \in {\mathbb {R}} \times [0, \infty ) \times {\mathbb {S}}^2, \quad t = x_0, \quad r = |x|, \quad \omega ^i = \tfrac{x^i}{r}, \end{aligned}$$

with the usual degeneracies of polar coordinates. We decompose the metric as follows:

1.22 $$\begin{aligned} g_{\alpha \beta } = m _{\alpha \beta } + h^0_{\alpha \beta } + h^1_{\alpha \beta }, \quad h^0_{\alpha \beta } = M \chi (\tfrac{r}{t+1})r^{-1}\delta _{\alpha \beta } \end{aligned}$$

where *M* is the ADM mass for *g*, $\chi = \chi (y)$ is a smooth cutoff function equal to 1 for $y\ge \frac{3}{4}$ and 0 for $y\le \frac{1}{4}$, such that $\partial \chi$ decays like $t^{-1}$, and $h^1$ is a small error which decays initially like $(1+|r|)^{-3/2-\alpha }$ for some $\alpha > 0$. Under this decomposition, our decay bounds take the form

1.23a $$\begin{aligned} M&< \varepsilon _g, \end{aligned}$$

1.23b $$\begin{aligned} \langle t+r^*\rangle |{\overline{{\widetilde{\partial }}}}{\mathcal {L}}_{{\widetilde{X}}}^Ih^1|+ \langle t-r^*\rangle |{\widetilde{\partial }}{\mathcal {L}}_{{\widetilde{X}}}^Ih^1|+|{\mathcal {L}}_{{\widetilde{X}}}^Ih^1|&< \varepsilon _g{\tau _{+}^{-1+\delta }}, \end{aligned}$$

1.23c $$\begin{aligned} \langle t-r^*\rangle |{\widetilde{\partial }}{\mathcal {L}}_{{\widetilde{X}}}^I h^1|_{{{\mathcal {L}}}{{\mathcal {T}}}} + |{\mathcal {L}}_{{\widetilde{X}}}^I h^1|_{{{\mathcal {L}}}{{\mathcal {T}}}}&< \varepsilon _g{\tau _{-}^{\gamma }}{\tau _{+}^{-1-\gamma +\delta }}. \end{aligned}$$

Here, $k\ge 11$ is an integer, *I* is a multiindex with $|I|\le k-6$, $\tau _{\pm }$ are defined in (1.29), the subscript ${{\mathcal {L}}}{{\mathcal {T}}}$ and the modified derivative norms $|{\widetilde{\partial }}\phi |, |{\overline{{\widetilde{\partial }}}}\phi |$ are defined, respectively, in (1.32) and (1.33), the set ${\widetilde{X}}\in \{{\widetilde{\partial }}_\alpha , {{\widetilde{\Omega }}_{{\alpha \beta }}}, {{\widetilde{S}}}\}$ is defined in (2.6), $\gamma > \frac{1}{2}$, and $\varepsilon _g>0$ is a small constant.

Additionally, for a small constant $\mu > 0$, we may define the metric weight

1.24 $$\begin{aligned} {w_\gamma }= {w_\gamma }(r^* - t) ={\left\{ \begin{array}{ll} 1+ (1+| r^* - t|)^{-2\mu } &{} r^* \le t, \\ 1 +(1+| r^* - t|)^{1+2\gamma } &{} r^* \ge t, \end{array}\right. } \end{aligned}$$

where $r^*$ is defined in (1.27). Then, for $|I| \le k$ we assume the $L^2$ bounds

1.25a $$\begin{aligned} \left\Vert |\partial {\mathcal {L}}_{{\widetilde{X}}}^Ih^1|{w_\gamma ^{{1/2}}}\right\Vert ^2_{L^2({\mathbb {R}}^3)} + \left\Vert {\tau _{-}^{-1}}|{\mathcal {L}}_{{\widetilde{X}}}^Ih^1|{w_\gamma ^{{1/2}}}\right\Vert ^2_{L^2({\mathbb {R}}^3)}&\le \varepsilon _g^2 (1+t)^{\delta }, \end{aligned}$$

1.25b $$\begin{aligned} \left\Vert \big (|\partial {\mathcal {L}}_{{\widetilde{X}}}^Ih^1|_{{{\widetilde{L}}}{{\widetilde{L}}}}+ |{{\overline{\partial }}}{\mathcal {L}}_{{\widetilde{X}}}^Ih^1|\big )({w_\gamma}\!\!')^{1/2}\right\Vert ^2_{L^2([0,T]\times {\mathbb {R}}^3)}&\le \varepsilon _g^2(1+T)^{\delta }, \end{aligned}$$

1.25c $$\begin{aligned} \left\Vert {\!\langle r^*\!\!\!-\!t\rangle }^{-1-s}{\!\langle r^*\!\!\!+\!t\rangle }^{-1+s}|{\mathcal {L}}_{{\widetilde{X}}}^Ih^{1\!}|_{{\mathcal {L}}{\mathcal {L}}} (w_\gamma ')^{1/2} \right\Vert _{L^2([0,T]\!\times {\mathbb {R}}^3)}&\le \!\varepsilon _g \end{aligned}$$

for $|I| \le k$. The fields we use are adaptations of the traditional commutator fields in Minkowski space. In addition to [16], they have been used by Oliver in [22], and later by Sterbenz and Oliver in [23] for a more general class of metrics. In the sequel we will show that these bounds follow from a bootstrap assumption for the Einstein field equations in harmonic coordinates,

1.26 $$\begin{aligned} \Box _gg_{\mu \nu } = {P}[\partial g, \partial g]_{\mu \nu }(g) + T_{\mu \nu } - \frac{1}{2}g_{\mu \nu }\text {tr}_g(T), \end{aligned}$$

where *P* is quadratic in derivatives of *g* and behaves nicely in the null decomposition. *T* will generally be smaller and decay faster than corresponding components of *P*, which appears in the vacuum equations, so the background spacetime of the coupled system may be approximated by solutions of the vacuum equations.

### The Modified Coordinates and Weights

The presence of the mass term in (1.22) means that, when attempting to establish a conformal Morawetz-type estimate, we cannot simply treat the background spacetime as a perturbation of Minkowski space. Instead, we use an interpolated tortoise coordinate $r^*$ and approximate optical functions ${u^{*{}}}= t + r^*$, ${\underline{u}^{*{}}}= t - r^*$. Since $h^0$ is supported when $r \gg M$, we do not have to worry about trapped geodesics, so we may use a conformal estimate rather than the purely radial estimate of [6]. This allows for a more intuitive treatment of the charged part of ${\textbf{F}}$. Similar estimates for Maxwell’s equations on Schwarzschild have been carried out by Anderson and Blue [1], and Sterbenz and Tataru [28], as well as for certain quasilinear wave equations by Lindblad and Tohaneanu [19].

Additionally, decay estimates on $h^1$ in the vacuum case mean that we cannot hope to recover the full conformal Morawetz estimate. We will instead prove a fractional Morawetz estimate, as in [18], with weights ${u^{*{2s}}}, {\underline{u}^{*{2s}}}$ which depend on the initial decay of $h^1$. We show the base estimate, with a discussion of the modified fields and the necessity of peeling estimates for *h*, in Sect. 2.6. A similar estimate for wave equations was shown by Lindblad and Schlue in [15]

We define the adapted tortoise coordinate

1.27 $$\begin{aligned} r^* = r + M\chi \ln (r), \end{aligned}$$

where $\chi$ is as in (1.22). It follows that $r^* = r$ in the far interior $r < (t+1)/4$ and $r^* = r+M\ln (r)$ for $r > 3(t+1)/4$. We then define the modified coordinates $({\widetilde{t}}, {\widetilde{x}})$ and approximate optical functions ${u^{*{}}}, {\underline{u}^{*{}}}$ by:

1.28 $$\begin{aligned} {\widetilde{t}}= {\widetilde{x}}^0 = t^* = t, \quad {\widetilde{x}}^i = \omega ^ir^*, \quad {u^{*{}}}= t - r^*, \quad {\underline{u}^{*{}}}= t + r^* \end{aligned}$$

It follows from (1.23) that $g^{\alpha \beta }\partial _\alpha {u^{*{}}}\partial _\beta {u^{*{}}}$ decays more rapidly along the light cone than $t-r$. Additionally, using the Japanese bracket

$$\begin{aligned} \langle y \rangle = (1+|y|^2)^{1/2}, \end{aligned}$$

we can define the optical weights

1.29 $$\begin{aligned} {\tau _{+}^{2}} = \langle {\underline{u}^{*{}}}\rangle ^2 \quad {\tau _{-}^{2}} = \langle {u^{*{}}}\rangle ^2 \quad {\tau _{0}}= {\tau _{-}}/{\tau _{+}}. \end{aligned}$$

Taking $\partial _r = \omega ^i\partial _i$ and , we can define1.30We have a natural modified null frame

1.31 $$\begin{aligned} {{\widetilde{L}}}= {\partial _{t^*}}+ {\partial _{r^*}}, \quad {\underline{{\widetilde{L}}}}= {\partial _{t^*}}- {\partial _{r^*}}, \quad {{\widetilde{S}}_{i}} = \tfrac{r}{r^*}S_i, \end{aligned}$$

where $S_i = \{S_1, S_2\}$ are piecewise defined fields forming an orthonormal frame tangent to the sphere (in the Minkowski metric). We define the sets

1.32 $$\begin{aligned} {{\mathcal {L}}}= \{{{\widetilde{L}}}\}, \quad {{\mathcal {T}}}= \{{{\widetilde{L}}}, {{\widetilde{S}}_{1}}, {{\widetilde{S}}_{2}}\}, \quad {{\mathcal {U}}}= \{{{\widetilde{L}}}, {\underline{{\widetilde{L}}}}, {{\widetilde{S}}_{1}}, {{\widetilde{S}}_{2}}\}, \end{aligned}$$

and use the following notation for partial norms

$$\begin{aligned} |T|_{{\mathcal {X}}{\mathcal {Y}}} = \sup _{X \in {\mathcal {X}}, Y \in {\mathcal {Y}}} |T_{XY}|. \end{aligned}$$

We additionally define

1.33 $$\begin{aligned} |{\widetilde{\partial }}\phi |^2 = \sum _{\widetilde{U}\in {{\mathcal {U}}}}| \widetilde{U} \phi |^2, \quad |{\overline{{\widetilde{\partial }}}} \phi |^2 = \sum _{\widetilde{T}\in {{\mathcal {T}}}}|\widetilde{T}\phi |^2 \end{aligned}$$

For norms of tensors where vector fields are not specified, we use the full null frame ${{\mathcal {U}}}$, e.g., for (0, 2)-tensors *T*, we have

$$\begin{aligned} |T| = |T|_{{{\mathcal {U}}}{{\mathcal {U}}}} \end{aligned}$$

Our modified null frame has two advantages: First, components of *g* and its Lie derivatives have improved bounds in the null decomposition, and second, these vectors commute well with the modified Lorentz fields used in [16], which is necessary in order to pass the nice weak null structure to derivatives. The peeling estimates obtained from this modification mirror those for Minkowski and are necessary in order to close the argument.

### The Main Theorem

Before stating our result, we define certain tensorial quantities which appear. For a generic 2-form ${\textbf{G}}$, we define the modified null decomposition:

1.34a $$\begin{aligned} {\alpha }_i[{\textbf{G}}]&= {\textbf{G}}_{{{\widetilde{L}}}{{\widetilde{S}}_{i}}}&\quad {\underline{\alpha }}_i[{\textbf{G}}]&= {\textbf{G}}_{{\underline{{\widetilde{L}}}}{{\widetilde{S}}_{i}}} \end{aligned}$$

1.34b $$\begin{aligned} \rho [{\textbf{G}}]&= \frac{1}{2}{\textbf{G}}_{{\underline{{\widetilde{L}}}}{{\widetilde{L}}}}&\sigma [{\textbf{G}}]&= \frac{1}{2}{\textbf{G}}_{{{\widetilde{S}}_{1}}{{\widetilde{S}}_{2}}} \end{aligned}$$

When there is no ambiguity, we remove the explicit dependence on ${\textbf{G}}$. Since the tangential terms are not uniquely defined, the following terms often show up in our calculations.1.35We additionally define the electromagnetic decomposition

1.36 $$\begin{aligned} {\textbf{E}}_i[{\textbf{G}}] = {\textbf{G}}_{0i}, \quad {\textbf{B}}_i[{\textbf{G}}] = (\star {\textbf{G}})_{0i}, \end{aligned}$$

where $\star {\textbf{G}}$ is the Hodge dual of ${\textbf{G}}$. We can decompose ${\textbf{E}}$ into its divergence-free and curl-free components, ${\textbf{E}}_{df}$ and ${\textbf{E}}_{cf}$, respectively.

Before stating our result, we define the norms governing our initial conditions. For a generic (0, *j*)-tensor ${\textbf{T}}$, we define:

1.37 $$\begin{aligned} \left\Vert {\textbf{T}}\right\Vert ^2_{H^{k, s_0}({\mathbb {R}}^3)} = \sum _{|I| \le k} \sum _{\alpha _i \in (0,3)}\int _{{\mathbb {R}}^3} (1+r^2)^{s_0+|I|}|{\underline{\nabla }}^I {\textbf{T}}({{\widetilde{\partial }}_{{\alpha _1}}},...{{\widetilde{\partial }}_{{\alpha _j}}})|^2 \, dx,\nonumber \\ \end{aligned}$$

where *I* is a multiindex. Here, ${\underline{\nabla }}$ and $\underline{D}$ are the covariant derivatives restricted to time slices.

Likewise, for a complex scalar field $\phi$, we have the corresponding quantity

1.38 $$\begin{aligned} \left\Vert \phi \right\Vert ^2_{H^{k, s_0}({\mathbb {R}}^3)} = \sum _{|I| \le k} \int _{{\mathbb {R}}^3} (1+r^2)^{s_0+ |I|}|\underline{D}^I \phi |^2 \, dx. \end{aligned}$$

#### Theorem 1.1

Take positive constants $s, s_0, \gamma , \mu , \delta , {{\overline{\mu }}}$ such that $\frac{1}{2}< s< 1< s_0 < 3/2$, $2\,s-1< \gamma <1$, $\gamma >1/2$, $\mu < 1/2$, and $4\max (\delta , {\overline{\mu }}) < \min (1-2\mu , s-\frac{1}{2}, 1-s, \gamma -\frac{1}{2}, 1-\gamma , 1+\gamma -2\,s)$. Additionally, take an integer $k \ge 11$.

There exist constants $\varepsilon _0$, $\varepsilon _1> 0$ such that if the metric, in the decomposition (1.22), satisfies (1.23) and (1.25) for $\varepsilon _g < \varepsilon _1$, and if we take initial conditions ${\textbf{E}}_0, {\textbf{B}}_0, \varvec{\phi }_0, \,\,\dot{\!\!\varvec{\phi }}_0$ for ${\textbf{F}}$ and $\varvec{\phi }$ satisfying

1.39 $$\begin{aligned} \left\Vert {\textbf{E}}_{0df} \right\Vert _{H^{k, s_0}({\mathbb {R}}^3)} + \left\Vert {\textbf{B}}_0 \right\Vert _{H^{k, s_0}({\mathbb {R}}^3)}+ \left\Vert D\varvec{\phi }_0 \right\Vert _{H^{k, s_0}({\mathbb {R}}^3)} + \Vert \,\,\dot{\!\!\varvec{\phi }}_0 \Vert _{H^{k, s_0}({\mathbb {R}}^3)} < \varepsilon \nonumber \\ \end{aligned}$$

at time $t = 0$ for $\varepsilon < \varepsilon _0$, then solutions to (1.14) exist for all time, with

$$\begin{aligned} {\textbf{E}}[{\textbf{F}}](0,x) = {\textbf{E}}_{0}, \quad {\textbf{B}}[{\textbf{F}}](0,x) = {\textbf{B}}_0, \quad \varvec{\phi }(0, x) = \varvec{\phi }_0, \quad D_t \varvec{\phi }(0,x) = \,\,\dot{\!\!\varvec{\phi }}_0 \end{aligned}$$

and satisfy the bounds 1.40a1.40b1.40c1.40d1.40e

#### Remark 1.2

More precise bounds are given in Theorem 7.2.

We outline the proof, which spans the remainder of this paper.

Section 2 opens with definitions and notation that we will use later on, and the bulk of the section is devoted to analytical identities and estimates, which follow from our bounds on $h^1$. We conclude this section by proving several Morawetz estimates, which provide motivation for the particular form of the conformal energy estimates we use, including the fractional Morawetz estimate, the modified null frame, and the weight *w*.

In Sects. 3 and 4, we establish fractional Morawetz-type estimates, which will be used to bound derivatives of ${\textbf{F}}^1$ and $\varvec{\phi }$, respectively, which are roughly adapted from [18].

Sections 5 and 6 establish $L^\infty$ estimates on field quantities. These are straightforward weighted Klainerman–Sobolev estimates, with some additional care taken to account for the contribution of the charge and certain error terms which arise from the fact that $u^*$ is only an approximate optical function. Additionally, we set up a Strichartz-type estimate, which will be useful to close the result.

In Sect. 7, we prove the main theorem, up to certain bounds on commutator terms which we show in Sects. 8 and 9.

Section 10 is an appendix in which we prove some common analytical estimates we use.

## Notation and Preliminary Identities

### General Notation

Here and in what follows, we will use ${\textbf{F}}$ and $\varvec{\phi }$ specifically to refer to solutions of the Maxwell–Klein–Gordon system. We will typically use (nonboldface) $\phi$ and $\psi$ to refer to generic scalar functions, and ${\textbf{G}}$ to refer to generic 2-forms. With some overload of notation, we define

2.1 $$\begin{aligned} {\textbf{J}}[\psi ]_\alpha = {\mathfrak {I}}(\psi \overline{D_\alpha \psi }), \quad {\textbf{J}}[{\textbf{G}}]_\alpha = \nabla ^\beta {\textbf{G}}_{\alpha \beta }. \end{aligned}$$

As we will be working in two different coordinate systems, we outline the differences here in order to ensure clarity. When we are working in harmonic coordinates, we will use Greek indices $\alpha , \beta , \ldots$ to refer to components with respect to this frame.

It will occasionally be simpler to describe concepts in terms of the generalized harmonic coordinates $({\widetilde{t}}, {\widetilde{x}})$, defined in (1.28). We will use *a*, *b*, *c*, *d* to refer to components in the modified frame. We will use ${\widetilde{g}}$, ${\widetilde{h}}^1$, ${\widetilde{\Gamma }}$ to refer to components of corresponding quantities. For purely geometric quantities, and in the null decomposition, we will use them interchangeably.

We will also use *i*, *j*, *k*, *l* to refer to spatial variables, which are raised and lowered with respect to *m* unless otherwise specified.

Indices are generally raised and lowered with respect to the metric *g*, with a few exceptions. For the background metrics *m* and ${{\widehat{m}}}$, we define $m^{\alpha \beta }$ and ${{\widehat{m}}}^{ab}$to be components of their respective inverses $m^{-1}$, ${{\widehat{m}}}^{-1}$. In Sect. 2.6.1, we will do everything with respect to *m*, and in Sect. 2.6.2 and a portion of Sect. 4, we will use ${{\widehat{m}}}$.

A sharp ($\sharp$) or flat ($\flat$) appearing in a Lie derivative denotes that we have raised or lowered (respectively) the index before applying the Lie derivative.

Given a covector $\omega _\alpha$ and a vector *X* with components $X^\alpha$, we say

$$\begin{aligned} \omega _X = \omega (X) = X^\alpha \omega _\alpha . \end{aligned}$$

We can extend this to (*k*, 0)-tensors, so that we use $F_{XY}$ and *F*(*X*, *Y*) interchangeably. Similarly, given a frame $\{X_i\}$, we define the decomposition of a (0, *k*) tensor *G* using

2.2 $$\begin{aligned} G^{\alpha _1\ldots \alpha _k} = \sum _{i_1, \ldots i_k = 1}^4 G^{X_{i_1} \ldots X_{i_k}}X_{i_1}^{\alpha _1}\ldots X_{i_k}^{\alpha _k} \end{aligned}$$

Additionally, we define the contraction operator $i_{{\widetilde{X}}}$ such that for a 2-form ${\textbf{G}}$, $(i_{{\widetilde{X}}}{\textbf{G}})({\widetilde{Y}}) = {\textbf{G}}({\widetilde{X}}, {\widetilde{Y}}).$

We take $a \lesssim b$ to mean

$$\begin{aligned} a \le C_{s, s_0, \delta , \gamma , \mu , {{\overline{\mu }}}, k}b, \end{aligned}$$

and similarly, $a \approx b$ means $a\lesssim b, b\lesssim a$. In particular, *C* may always be chosen independently of $\varepsilon , \varepsilon _g$, and *T*, for suitable $\varepsilon _0, \varepsilon _1$. As we will be revising the values of *C* and $\varepsilon _1$ throughout the course of the proof, we include the following requirement: If a statement is true for a given choice of $\varepsilon _1$, then it is true for all $\varepsilon '_1\in (0, \varepsilon _1]$ without changing the implicit constant. Therefore, after every step, we may increase *C* and decrease the $\varepsilon$ quantities accordingly without jeopardizing earlier results.

Subscripts in $L^p$ norms will denote the variable of integration and will be taken over a subset of ${\mathbb {R}}^3\times [0,T]$; i.e., $L^p_x$ is the integral in space, and $L^p_t$ is in time. $L^p_x$ norms will be assumed to be taken at time *t*, unless otherwise specified. Mixed norms will be denoted using $L^p_t(L^q_x)$, etc. Norms with only a numerical subscript *p* will denote the spacetime norm:

$$\begin{aligned} \left\Vert \phi \right\Vert _p = \left\Vert \phi \right\Vert _{L^p_t(L^p_x)} = \left\Vert \phi \right\Vert _{L^p([0,T]\times {\mathbb {R}}^3)}. \end{aligned}$$

Norms on other domains will be unambiguously denoted. We will use $\Sigma _\tau$ to mean $\{t = \tau \}\times {\mathbb {R}}^3$.

### Modified Coordinates and Vector Fields

Recalling the definition (1.28), we define the coordinate transformation symbols

2.3 $$\begin{aligned} A_\alpha ^a = \partial _\alpha \widetilde{x}^a, \quad \widetilde{A}_a^\alpha = {\widetilde{\partial }}_a x^\alpha , \end{aligned}$$

so, e.g.

$$\begin{aligned} \partial _\alpha = A_\alpha ^a {\widetilde{\partial }}_a, \quad {\widetilde{\partial }}_a = \widetilde{A}_a^\alpha \partial _\alpha . \end{aligned}$$

Straightforward calculation gives

2.4a $$\begin{aligned} A_\alpha ^0&= \delta _\alpha ^0, \end{aligned}$$

2.4b $$\begin{aligned} A_0^i&= -M\omega ^i\tfrac{r}{(t+1)^2}\chi '\ln (r), \end{aligned}$$

2.4c $$\begin{aligned} A_i^j&= \big (1+\tfrac{M\chi }{r}\big )\delta _i^j + \tfrac{M\chi \ln r - M\chi }{r}(\delta _i^j - \omega _i\omega ^j) + \omega _i\omega ^j\big (\tfrac{M}{t+1}\chi '\ln r\big ). \end{aligned}$$

Then, the following bounds hold:

2.5 $$\begin{aligned} |A_\alpha ^a - \delta _\alpha ^a| + |\widetilde{A}^\alpha _a - \delta ^\alpha _a|\lesssim \tfrac{M\lceil \chi \rceil \ln r}{r}, \quad |\partial _\beta A_\alpha ^a| + |\partial _\beta \widetilde{A}^\alpha _a| \lesssim \tfrac{M\lceil \chi \rceil \ln r}{r^2}. \end{aligned}$$

This follows from straightforward calculation for $A_\alpha ^a$, and the identity $\widetilde{A}_a^\alpha A_\alpha ^b = \delta _a^b$.

We define the modified commutator fields

2.6 $$\begin{aligned} \widetilde{{\mathbb {L}}}= & {} \{{\widetilde{\partial }}_a, \quad {{\widetilde{\Omega }}_{{ij}}} = {\widetilde{x}}^{i}{\widetilde{\partial }}_j - {\widetilde{x}}^j{\widetilde{\partial }}_i = \Omega _{ij}, \quad \nonumber \\ {{\widetilde{\Omega }}_{{0j}}}= & {} {\widetilde{t}}{\widetilde{\partial }}_j + {\widetilde{x}}^j{\widetilde{\partial }}_0, \quad {{\widetilde{S}}}= {\widetilde{t}}{\widetilde{\partial }}_0 + {\widetilde{x}}^i{\widetilde{\partial }}_i\}. \end{aligned}$$

The notation ${\widetilde{X}}, {\widetilde{Y}}$ always refer to vectors in this set, and ${\widetilde{X}}^I$ will mean an (ordered) set of these vectors multiindexed by *I*. We can define all other possible values of ${{\widetilde{\Omega }}_{{ab}}}$ with the convention ${{\widetilde{\Omega }}_{{ab}}} = -{{\widetilde{\Omega }}_{{ba}}}$. Then, we define

2.7 $$\begin{aligned} c_{{\widetilde{X}}} = {\left\{ \begin{array}{ll} 2 &{} {\widetilde{X}}= {{\widetilde{S}}}, \\ 0 &{} {\widetilde{X}}= {\widetilde{\partial }}_a\text { or } {\widetilde{X}}= {{\widetilde{\Omega }}_{{ab}}}. \end{array}\right. } \end{aligned}$$

For $M=0$, ${\mathcal {L}}_{{\widetilde{X}}}m = c_{{\widetilde{X}}}m$. Given a collection of Lorentz fields ${\widetilde{X}}$ and a multiindex *I*, we define $c_{{\widetilde{X}}}^I$ to be the product of $c_{{\widetilde{X}}}$ for each ${\widetilde{X}}$ indexed by *I*. Next, recalling (1.31), we define the radial Lorentz boost field

2.8 $$\begin{aligned} {{\widetilde{\Omega }}_{{0r^*}}} = \sum _i\omega ^i{{\widetilde{\Omega }}_{{0i}}} = t^*{\partial _{r^*}}+ r^*{\partial _{t^*}}= \tfrac{1}{2}\left( {\underline{u}^{*{}}}{{\widetilde{L}}}- {u^{*{}}}{\underline{{\widetilde{L}}}}\right) . \end{aligned}$$

Then,2.9which will simplify later estimates. The corresponding commutators are:

2.10a $$\begin{aligned}&{[}{\widetilde{\partial }}_a, {\widetilde{\partial }}_b] = 0, \quad [{\widetilde{\partial }}_a, {{\widetilde{\Omega }}_{{ij}}}] = \delta _{a[i}{\widetilde{\partial }}_{j]}, \quad [{\widetilde{\partial }}_a, {{\widetilde{S}}}] = {\widetilde{\partial }}_a \end{aligned}$$

2.10b $$\begin{aligned}&{[}{{\widetilde{S}}}, {{\widetilde{\Omega }}_{{ab}}}] = 0, \quad [{\widetilde{\partial }}_a, {{\widetilde{\Omega }}_{{0b}}}] = \delta _{a(0}{\widetilde{\partial }}_{b)}, \quad [{{\widetilde{\Omega }}_{{0i}}}, {{\widetilde{\Omega }}_{{jk}}}] = \delta _{ij}{{\widetilde{\Omega }}_{{0k}}} - \delta _{ik}{{\widetilde{\Omega }}_{{0j}}} \end{aligned}$$

2.10c $$\begin{aligned}&[{{\widetilde{\Omega }}_{{0i}}}, {{\widetilde{\Omega }}_{{0j}}}] ={{\widetilde{\Omega }}_{{ij}}}, \quad [{{\widetilde{\Omega }}_{{ij}}}, {{\widetilde{\Omega }}_{{kl}}}] = -\delta _{ik}{{\widetilde{\Omega }}_{{jl}}} + \delta _{il}{{\widetilde{\Omega }}_{{jk}}} + \delta _{jk}{{\widetilde{\Omega }}_{{il}}} - \delta _{jl}{{\widetilde{\Omega }}_{{ik}}}. \end{aligned}$$

as well as 2.11a2.11b2.11c2.11d2.11e

Here, *a*, *b*,  and *c* are homogeneous functions of degree 0 in *r* which satisfy the conditions

2.12 $$\begin{aligned} a_{i1}^1= & {} a_{i2}^2 = b_{ij1}^1 = b_{ij2}^2 = c_{i1}^1 = c_{i2}^2 = 0, \quad \nonumber \\ a_{i1}^2 + a_{i2}^1= & {} b_{ij1}^2 + b_{ij2}^1 = c_{i1}^2 + c_{i2}^1 = 0. \end{aligned}$$

We recall the Lie derivative formulas for one- and two-forms, respectively:

2.13 $$\begin{aligned} ({\mathcal {L}}_{X}\omega )_Y&= X(\omega _Y) - \omega ([X,Y]), \end{aligned}$$

2.14 $$\begin{aligned} ({\mathcal {L}}_{X} {\textbf{G}})_{YZ}&= X({\textbf{G}}_{YZ}) - {\textbf{G}}([X,Y], Z) - {\textbf{G}}(Y, [X,Z]). \end{aligned}$$

#### Lemma 2.1

Given a two-form ${\textbf{G}}$, and ${\tau _{0}^{}}= {\tau _{-}^{}}/{\tau _{+}^{}}$, and for a set of commutator fields ${\widetilde{X}}^I$, it follows that

2.15a $$\begin{aligned} {\widetilde{X}}^I({\alpha }_i[{\textbf{G}}])&\lesssim \sum _{\begin{array}{c} |J| \le |I| \end{array}}|\alpha [{\mathcal {L}}_{{\widetilde{X}}}^J{\textbf{G}}]| + |{\tau _{0}^{}}\rho [{\mathcal {L}}_{{\widetilde{X}}}^J{\textbf{G}}]| + |{\tau _{0}^{}}\sigma [{\mathcal {L}}_{{\widetilde{X}}}^J{\textbf{G}}]| + {\tau _{0}^{2}}|{\underline{\alpha }}[{\mathcal {L}}_{{\widetilde{X}}}^J{\textbf{G}}]|, \end{aligned}$$

2.15b $$\begin{aligned} {\widetilde{X}}^I(\rho [{\textbf{G}}])&\lesssim \sum _{\begin{array}{c} |J| \le |I| \end{array}}|\alpha [{\mathcal {L}}_{{\widetilde{X}}}^J{\textbf{G}}]| + |\rho [{\mathcal {L}}_{{\widetilde{X}}}^J{\textbf{G}}]| + |\sigma [{\mathcal {L}}_{{\widetilde{X}}}^J{\textbf{G}}]| + {\tau _{0}^{}}|{\underline{\alpha }}[{\mathcal {L}}_{{\widetilde{X}}}^J{\textbf{G}}]|, \end{aligned}$$

2.15c $$\begin{aligned} {\widetilde{X}}^I(\sigma [{\textbf{G}}])&\lesssim \sum _{\begin{array}{c} |J| \le |I| \end{array}}|\alpha [{\mathcal {L}}_{{\widetilde{X}}}^J{\textbf{G}}]| + |\rho [{\mathcal {L}}_{{\widetilde{X}}}^J{\textbf{G}}]| + |\sigma [{\mathcal {L}}_{{\widetilde{X}}}^J{\textbf{G}}]| + {\tau _{0}^{}}|{\underline{\alpha }}[{\mathcal {L}}_{{\widetilde{X}}}^J{\textbf{G}}]|, \end{aligned}$$

2.15d $$\begin{aligned} {\widetilde{X}}^I({\underline{\alpha }}_i[{\textbf{G}}])&\lesssim \sum _{\begin{array}{c} |J| \le |I| \end{array}}|\alpha [{\mathcal {L}}_{{\widetilde{X}}}^J{\textbf{G}}]| + |\rho [{\mathcal {L}}_{{\widetilde{X}}}^J{\textbf{G}}]| + |\sigma [{\mathcal {L}}_{{\widetilde{X}}}^J{\textbf{G}}]| + |{\underline{\alpha }}[{\mathcal {L}}_{{\widetilde{X}}}^J{\textbf{G}}]|. \end{aligned}$$

when $$r^* \ge \tfrac{t+1}{4}$$.

#### Proof

This follows from induction using (2.11). Specifically, we define the class $\Phi ^M$ to be the set of functions, which can be written as a finite sum:

$$\begin{aligned} \sum _{m + n\ge M}\Big (\tfrac{1}{r^*}\Big )^m\Big (\tfrac{{u^{*{}}}}{r^*}\Big )^nP(\tfrac{r^*}{t+1}, \tfrac{t}{r^*}, \omega ), \end{aligned}$$

for a compactly supported function *P*. Then, if $\phi \in \Phi ^m$, ${\widetilde{X}}\phi \in \Phi ^m$. Additionally, for any ${\widetilde{X}}$, we can write

2.16a $$\begin{aligned} {[}{{\widetilde{L}}}, {\widetilde{X}}]&= \phi ^0_1{{\widetilde{L}}}+ \sum _j\phi ^1_{1j}{{\widetilde{S}}_{j}}, \end{aligned}$$

2.16b $$\begin{aligned} {[}{{\widetilde{S}}_{i}}. {\widetilde{X}}]&= \phi ^0_{2i}{{\widetilde{L}}}+\sum _j\phi ^0_{2ij}{{\widetilde{S}}_{j}} + \phi ^1_{2i}{\underline{{\widetilde{L}}}}, \end{aligned}$$

2.16c $$\begin{aligned} {[}{\underline{{\widetilde{L}}}}, {\widetilde{X}}]&= \phi ^0_{3}{{\widetilde{L}}}+\sum _j\phi ^0_{3j}{{\widetilde{S}}_{j}} + \phi ^0_{4}{\underline{{\widetilde{L}}}}, \end{aligned}$$

where all $\phi ^0$ functions are in $\Phi ^0$, and all $\phi ^1$ functions are in $\Phi ^1$. Repeating this and noting that if $\phi \in \Phi ^m$, then, for some *C* depending on $\phi$, and for $r^* > \frac{t+1}{4}$,

$$\begin{aligned} |\phi |\le C\big (\tfrac{\langle {u^{*{}}}\rangle }{r^*}\big )^m. \end{aligned}$$

$$\square $$

### Assumptions on the Metric

The modified null frame gives better decay for certain components of the metric, which we will quantify in this section. We define

2.17 $$\begin{aligned} g_{\alpha \beta } = m_{\alpha \beta } + h^0_{\alpha \beta } + h^1_{\alpha \beta }, \quad g^{\alpha \beta } = m^{\alpha \beta } + H_0^{\alpha \beta } + H_1^{\alpha \beta }, \end{aligned}$$

where

2.18 $$\begin{aligned} h^0_{\alpha \beta } = \tfrac{M\chi }{r}\delta _{\alpha \beta }, \quad H_0^{\alpha \beta } = - \tfrac{M\chi }{r}\delta ^{\alpha \beta }. \end{aligned}$$

We define ${\widehat{m}}$ to be the Minkowski metric with respect to the new frame:

2.19 $$\begin{aligned} {\widehat{m}} = -d{\widetilde{t}}^{\,2} + \sum _{i}d\widetilde{x_i}^2 \end{aligned}$$

#### Proposition 2.2

Define

2.20 $$\begin{aligned} m^0_{\alpha \beta } = m_{\alpha \beta } + h^0_{\alpha \beta }, \quad m_0^{\alpha \beta } = m^{\alpha \beta } + H_0^{\alpha \beta }, \end{aligned}$$

as well as2.21Additionally, define  using (2.21) for $1\le \alpha , \beta \le 3$ and 0 if $\alpha = 0$ or $\beta = 0$. Then,2.22and2.23

#### Proof

We sketch out the proof, as a more thorough version appears in the sequel. This is trivially true when $\chi = 0$, so we may reduce this to standard derivatives in the $\widetilde{x}_a$ frame without worrying about behavior at the origin. Using (2.4), every component of the difference can be written in the form

$$\begin{aligned} \frac{MP_1(\tfrac{\chi }{r}, \tfrac{\chi \ln r}{r}, \tfrac{\chi '\ln r}{r}, \frac{t}{r}, \omega )}{1+MP_2(\tfrac{\chi }{r}, \tfrac{\chi \ln r}{r}, \tfrac{\chi '\ln r}{r}, \frac{t}{r}, \omega )}, \end{aligned}$$

where each term in $P_2$ has a factor of $\tfrac{\chi }{r}$, $\tfrac{\chi \ln r}{r}$, or $\tfrac{\chi '\ln r}{r}$, and each term in $P_1$ contains a factor of $(\tfrac{\chi }{r})^2$, $(\tfrac{\chi \ln r}{r})^2$, $\tfrac{\chi }{r}\tfrac{\chi \ln r}{r}$, or $\tfrac{\chi '\ln r}{r}$. By induction, this holds also for Lie derivatives, if we let the polynomials depend on $\frac{1}{r}$ and $\frac{ \ln r}{r}$ terms containing higher derivatives of $\chi$. The bound (2.22) directly follows, noting that the denominator is uniformly bounded below for small *M*. The bound (2.23) follows from a similar argument after expressing each quantity in the modified coordinate system. $\square$

#### Remark 2.3

In this modified frame, we note that tangential components of the metric decay like $r^{-1}\ln r$, whereas corresponding components in the standard null frame decay like $r^{-1}$. This arises from the way the tangential components of the metric “pick up” the contribution of the ADM mass. Since these components will be coupled with tangential derivatives and components of $\varvec{\phi }$ and ${\textbf{F}}$, which have sharper decay rates, this will not play a significant role in our later analysis.

Now we look at $h^1, H_1$. Since the main result is in a sense a part of a bootstrap argument for the full EMKG system, we assume $L^\infty$ estimates on low derivatives of $h^1$ and $H_1$ and $L^2$ estimates on high derivatives of $h^1$ and $H_1$. For now, we use the abstract *h* for both (i.e., if an estimate is true for *h*, then it holds for $h^1$ and $H_1$, with indices raised and lowered as appropriate). We look at the set of metrics *g* satisfying the $L^\infty$ norms

2.24a $$\begin{aligned} M&< \varepsilon _g, \end{aligned}$$

2.24b $$\begin{aligned} |{\mathcal {L}}_{{\widetilde{X}}}^Ih|&< \varepsilon _g{\tau _{+}^{-1+\delta }}, \end{aligned}$$

2.24c $$\begin{aligned} |{\mathcal {L}}_{{\widetilde{X}}}^I h|_{{{\mathcal {L}}}{{\mathcal {T}}}}&< \varepsilon _g{\tau _{0}^{\gamma }}{\tau _{+}^{-1+\delta }}, \end{aligned}$$

for a multiindex $I, |I| \le k - 6$, and

2.25a $$\begin{aligned} \left\Vert |\partial {\mathcal {L}}_{{\widetilde{X}}}^Ih|w_\gamma ^{1/2}\right\Vert _{L^2_x} + \left\Vert {\tau _{-}^{-1}}|{\mathcal {L}}_{{\widetilde{X}}}^Ih|w_\gamma ^{1/2}\right\Vert _{L^2_x}&< \varepsilon _g (1+t)^{\delta /2}, \end{aligned}$$

2.25b $$\begin{aligned} \left\Vert \big (|{{\overline{\partial }}}{\mathcal {L}}_{{\widetilde{X}}}^Ih| + |\partial {\mathcal {L}}_{{\widetilde{X}}}^Ih|_{{{\widetilde{L}}}{{\widetilde{L}}}}\big ) (w'_g)^{1/2}\right\Vert _{L^2_t(L^2_x)}&< \varepsilon _g(1+T)^{\delta /2}, \end{aligned}$$

2.25c $$\begin{aligned} \left\Vert {\tau _{+}^{s-1}}{\tau _{-}^{-1-s}}|{\mathcal {L}}_{{\widetilde{X}}}^Ih|_{{{\widetilde{L}}}{{\widetilde{L}}}}\right\Vert _{L^2_t(L^2_x)}&< \varepsilon _g \end{aligned}$$

for $$|I| \le k$$ and

2.26 $$\begin{aligned} w_\gamma = {\left\{ \begin{array}{ll} 1 + (1+t-r^*)^{-2\mu }&{} r^* \le t, \\ 1+(1 + r^* - t)^{1+2\gamma } &{} r^* \ge t. \end{array}\right. } \end{aligned}$$

In the energy estimate which will appear in the sequel, the second norm on the left of (2.25a) follows from a weighted Hardy estimate. The norms in (2.25b) follow from a weighted energy estimate and the harmonic coordinate condition, respectively.

In order for the estimate to close, we will assume $0<4\delta , 4{\overline{\mu }} <\min (1-2\mu , s-\frac{1}{2}, 1-s, \gamma - \frac{1}{2}, 1-\gamma , 1+\gamma -2\,s)$, $2\mu + \delta < 1$, and $k\ge 11$, and $\varepsilon _g>0$ is sufficiently small. Due to the freedom in choosing harmonic coordinates, we assume the initial conditions that at time $t = 0$,

2.27 $$\begin{aligned} g_{00} = -\left( 1-\tfrac{M\chi }{r}\right) , \quad g_{0i} = 0 \end{aligned}$$

We have analogous estimates on components of the raised metric. In particular, if we look at components of $g^{-1}$ in the null frame we see that every error term appearing in $g^{{\underline{{\widetilde{L}}}}{\underline{{\widetilde{L}}}}}$ and $g^{{\underline{{\widetilde{L}}}}{{\widetilde{S}}_{i}}}$ contains a term decaying like ${\tau _{0}^{\gamma '}}{\tau _{+}^{-1+\delta }}$ or ${\tau _{+}^{-2+2\delta }}$. It therefore follows from (2.23) and (2.24) that

2.28a $$\begin{aligned} |g^{{\underline{{\widetilde{L}}}}{\underline{{\widetilde{L}}}}}| + |g^{{\underline{{\widetilde{L}}}}{{\widetilde{S}}}}|&\lesssim \varepsilon _g{\tau _{0}^{\gamma }}{\tau _{+}^{-1+\delta }}, \end{aligned}$$

2.28b $$\begin{aligned} \left| g^{{{\widetilde{L}}}{\underline{{\widetilde{L}}}}} + \frac{1}{2}\right| + \left| g^{{{\widetilde{S}}_{i}}{{\widetilde{S}}_{i}}} - 1\right|&\lesssim \varepsilon _g{\tau _{+}^{-1+\delta }}, \end{aligned}$$

2.28c $$\begin{aligned} |g^{{{\widetilde{L}}}{{\widetilde{L}}}}| + |g^{{{\widetilde{S}}_{1}}{{\widetilde{S}}_{2}}}|+ |g^{{{\widetilde{L}}}{{\widetilde{S}}_{i}}}|&\lesssim \varepsilon _g{\tau _{+}^{-1+\delta }}. \end{aligned}$$

### Lie Derivatives and Commutators

We recall the definition of the deformation tensor

2.29 $$\begin{aligned} {^{({X})}}\pi = {\mathcal {L}}_{X}g = 2\cdot \text {symm}(\nabla X). \end{aligned}$$

If *X* is Killing or conformal Killing, ${^{({X})}}\pi$ is 0 or a scalar multiple of *g*, respectively. In general, we cannot assume any Killing or conformal Killing fields. However, if *g* is close to Minkowski, we can still establish useful estimates on ${^{({X})}}\pi$, as follows.

We first take a notational tool $\widetilde{{\mathcal {L}}}_{{\widetilde{X}}}$, defined for the modified Lorentz fields, such that

2.30a $$\begin{aligned} \widetilde{{\mathcal {L}}}_{{{\widetilde{X}}}}(T^{\alpha \beta })&= ({\mathcal {L}}_{{{\widetilde{X}}}}T)^{\alpha \beta } + c_{{\widetilde{X}}}T^{\alpha \beta }, \end{aligned}$$

2.30b $$\begin{aligned} \widetilde{{\mathcal {L}}}_{{{\widetilde{X}}}}(T_{\alpha \beta })&= ({\mathcal {L}}_{{{\widetilde{X}}}}T)_{\alpha \beta } - c_{{\widetilde{X}}}T_{\alpha \beta }, \end{aligned}$$

where the $c_{{\widetilde{X}}}$ are the Killing coefficients defined in (2.7). We define the iterated reduced deformation tensors

2.31a $$\begin{aligned} {{^{({{{\widetilde{X}}}^I})}{\widetilde{\pi }}}}_{\alpha \beta }&:= (\widetilde{{\mathcal {L}}}_{{{\widetilde{X}}}}^Ig)_{\alpha \beta }, \end{aligned}$$

2.31b $$\begin{aligned} {{^{({{{\widetilde{X}}}^I})}{\widetilde{\Pi }}}}^{\alpha \beta }&:= (\widetilde{{\mathcal {L}}}_{{{\widetilde{X}}}}^Ig^{-1})^{\alpha \beta }. \end{aligned}$$

These are similar up to a sign, which follow from expanding ${{^{({{{\widetilde{X}}}^I})}{\widetilde{\pi }}}} = \widetilde{{\mathcal {L}}}_{{{\widetilde{X}}}}^I m^0 + \widetilde{{\mathcal {L}}}_{{{\widetilde{X}}}}^Ih^1$, and bounding the two terms on the right using (2.22) and either (2.24) or (2.25). Additionally, the reduced deformation tensors satisfy

2.32 $$\begin{aligned} {{^{({{{\widetilde{X}}}})}{\widetilde{\pi }}}}^{\alpha \beta } = -{{^{({{{\widetilde{X}}}})}{\widetilde{\Pi }}}}^{\alpha \beta }, \quad {{^{({{{\widetilde{X}}}^I})}{\widetilde{\pi }}}}^{\alpha \beta } = -{{^{({{{\widetilde{X}}}^I})}{\widetilde{\Pi }}}}^{\alpha \beta } + O\left( \sum _{|I'|\le |I|}|{{^{({{{\widetilde{X}}}^{I'}})}{\widetilde{\Pi }}}}|_{{{\mathcal {U}}}{{\mathcal {U}}}}^2\right) ,\nonumber \\ \end{aligned}$$

which follow from applying $\widetilde{{\mathcal {L}}}_{{\widetilde{X}}}^I$ to the identity

$$\begin{aligned} g_{\alpha \beta } = g_{\alpha \gamma }g^{\gamma \delta }g_{\delta \beta } \end{aligned}$$

once and multiple times, respectively. For any vector field *X*, taking the trace of (2.29) gives

2.33 $$\begin{aligned} \nabla \cdot X = \tfrac{1}{2} g^{\alpha \beta }({\mathcal {L}}_{X}g)_{\alpha \beta }, \end{aligned}$$

and consequently, subtracting off $c_X\text {tr}(g)$ and expanding the Lie derivatives using (2.30) gives

2.34 $$\begin{aligned} \left| {\widetilde{Y}}\left( {\mathcal {L}}_{{\widetilde{X}}}^I(\nabla \cdot {\widetilde{X}}_1)\right) \right|\lesssim & {} \sum _{\begin{array}{c} |I_1|+|I_2| \le |I|+1 \end{array}}\left| {\widetilde{Y}}\left( g^{\alpha \beta }{{^{({{\widetilde{X}}^{I_2}})}{\widetilde{\pi }}}}_{\alpha \beta }\right) \right| \nonumber \\{} & {} + \left| {\widetilde{Y}}\left( {{^{({{\widetilde{X}}^{I_1}})}{\widetilde{\Pi }}}}^{\alpha \beta }{{^{({{\widetilde{X}}^{I_2}})}{\widetilde{\pi }}}}_{\alpha \beta }\right) \right| . \end{aligned}$$

These quantities also satisfy analogous estimates to (2.24):

#### Proposition 2.4

For sufficiently small $\varepsilon _g$, the following estimates follow from (2.24)

2.35a $$\begin{aligned} |{{^{({{{\widetilde{X}}}^I})}{\widetilde{\pi }}}}| +|{{^{({{{\widetilde{X}}}^I})}{\widetilde{\Pi }}}}|&\lesssim \varepsilon _g{\tau _{+}^{-1+\delta }}, \end{aligned}$$

2.35b $$\begin{aligned} |{{^{({{\widetilde{X}}^I})}{\widetilde{\pi }}}}_{{{\mathcal {L}}}{{\mathcal {T}}}}|+|{{^{({{\widetilde{X}}^I})}{\widetilde{\Pi }}}}_{{{\mathcal {L}}}{{\mathcal {T}}}}|&\lesssim \varepsilon _g{\tau _{-}^{\gamma }}{\tau _{+}^{-\gamma -1+\delta }}, \end{aligned}$$

for $$|I| \le k - 6$$. Additionally,

2.36a $$\begin{aligned} |{{^{({{{\widetilde{X}}}^I})}{\widetilde{\pi }}}} - \widetilde{{\mathcal {L}}}_{{\widetilde{X}}}^I h^1| + |{{^{({{{\widetilde{X}}}^I})}{\widetilde{\Pi }}}} - \widetilde{{\mathcal {L}}}_{{\widetilde{X}}}^I H_1|&\lesssim \varepsilon _g{\tau _{+}^{-1+\delta }}, \end{aligned}$$

2.36b $$\begin{aligned} |{{^{({{{\widetilde{X}}}^I})}{\widetilde{\pi }}}} - \widetilde{{\mathcal {L}}}_{{\widetilde{X}}}^I h^1|_{{{\mathcal {L}}}{{\mathcal {T}}}} + |{{^{({{{\widetilde{X}}}^I})}{\widetilde{\Pi }}}} - \widetilde{{\mathcal {L}}}_{{\widetilde{X}}}^I H_1|_{{{\mathcal {L}}}{{\mathcal {T}}}}&\lesssim \varepsilon _g{\tau _{-}^{\gamma }}{\tau _{+}^{-\gamma -1+\delta }}, \end{aligned}$$

for all *I*, where the constant in $\lesssim$ depends on *I*.

#### Proof

These follow directly from noting

$$\begin{aligned} \widetilde{{\mathcal {L}}}_{{\widetilde{X}}}{{\widehat{m}}}= 0, \quad \widetilde{{\mathcal {L}}}_{{\widetilde{X}}} g = \widetilde{{\mathcal {L}}}_{{\widetilde{X}}}(g-{{\widehat{m}}}) \end{aligned}$$

in both the upper and lower indices, and iterating the estimates (2.22) and (2.23). $\square$

We now commute standard and covariant derivatives through Lie derivatives. First, for standard derivatives $\partial _\gamma$, and for tensors $T^{\alpha _1\alpha _2...\alpha _m}_{\beta _1\beta _2...\beta _n}$, we have that

2.37 $$\begin{aligned}{}[\partial _\gamma , {\mathcal {L}}_{X}]T&= -(\partial _\gamma \partial _\delta X^{\alpha _1})T^{\delta \alpha _2...\alpha _m}_{\beta _1\beta _2...\beta _n} - ... -(\partial _\gamma \partial _\delta X^{\alpha _m})T^{\alpha _1\alpha _2...\delta }_{\beta _1\beta _2...\beta _n} \nonumber \\&\quad + (\partial _\gamma \partial _{\beta _1} X^{\delta })T^{\alpha _1\alpha _2...\alpha _m}_{\delta \beta _2...\beta _n} + ... + (\partial _\gamma \partial _{\beta _n} X^{\delta })T^{\alpha _1\alpha _2...\alpha _m}_{\beta _1\beta _2...\delta }. \end{aligned}$$

We have an analogous result for the covariant derivative:

2.38 $$\begin{aligned}{}[\nabla _\gamma , {\mathcal {L}}_{X}]T&= -(\nabla _\gamma \nabla _\delta X^{\alpha _1})T^{\delta \alpha _2...\alpha _m}_{\beta _1\beta _2...\beta _n} - ... -(\nabla _\gamma \nabla _\delta X^{\alpha _m})T^{\alpha _1\alpha _2...\delta }_{\beta _1\beta _2...\beta _n} \nonumber \\&\quad + (\nabla _\gamma \nabla _{\beta _1} X^{\delta })T^{\alpha _1\alpha _2...\alpha _m}_{\delta \beta _2...\beta _n} + ... + (\nabla _\gamma \nabla _{\beta _n} X^{\delta })T^{\alpha _1\alpha _2...\alpha _m}_{\beta _1\beta _2...\delta }. \end{aligned}$$

In each case, if *T* is a scalar, the corresponding commutator is 0. In the Minkowski metric, these are again 0 whenever *X* is a Lorentz field, as $X^\alpha$ is constant or linear in the standard frame.

For all vector fields *X* and all antisymmetric (2, 0)-tensors ${\textbf{G}}$, we have the identity

2.39 $$\begin{aligned}{}[\nabla _\beta , {\mathcal {L}}_{X}]{\textbf{G}}^{\alpha \beta } = -(\nabla _\delta \nabla _\beta X^\beta ){\textbf{G}}^{\alpha \delta }. \end{aligned}$$

This is straightforward to prove:

$$\begin{aligned}{}[\nabla _\beta , {\mathcal {L}}_{X}]{\textbf{G}}^{\alpha \beta }&= \nabla _\beta \left( X^\delta \nabla _\delta {\textbf{G}}^{\alpha \beta } - (\nabla _\delta X^\alpha ){\textbf{G}}^{\delta \beta } - (\nabla _\delta X^\beta ){\textbf{G}}^{\alpha \delta }\right) \\&\quad - X^\delta \nabla _\delta \nabla _\beta {\textbf{G}}^{\alpha \beta } + (\nabla _\delta X^\alpha )\nabla _\beta {\textbf{G}}^{\delta \beta } \\&= X^\delta [\nabla _\beta , \nabla _\delta ]{\textbf{G}}^{\alpha \beta } - (\nabla _\beta \nabla _\delta X^\alpha ){\textbf{G}}^{\delta \beta } - (\nabla _\beta \nabla _\delta X^\beta ){\textbf{G}}^{\alpha \delta }. \end{aligned}$$

Expanding the first term using the Riemann curvature tensor, symmetrizing the derivatives in the middle term and commuting the derivatives in the last term, then taking advantage of the antisymmetry of ${\textbf{G}}$ and the Bianchi identity

$$\begin{aligned} R^\alpha _{\beta \gamma \delta } + R^\alpha _{\gamma \delta \beta } + R^{\alpha }_{\delta \beta \gamma } = 0 \end{aligned}$$

gives us the desired identity.

Likewise, we can define the complex Lie derivative in a method similar to (1.2).

2.40 $$\begin{aligned} {{\mathcal {L}}_{X}^{{\mathbb {C}}{ }}}(\phi \otimes T) = D_X\phi \otimes T + \phi \otimes {\mathcal {L}}_{X}T, \end{aligned}$$

or equivalently, after choosing a section ${\textbf{1}}_V$,

2.41 $$\begin{aligned} {{\mathcal {L}}_{X}^{{\mathbb {C}}{ }}} = {\mathcal {L}}_{X} + i{\textbf{A}}_X. \end{aligned}$$

This implies the Leibniz rules for complex-valued tensors $T_1, T_2$,

2.42 $$\begin{aligned} {{\mathcal {L}}_{X}^{{\mathbb {C}}{ }}}(T_1\otimes T_2)= & {} ({\mathcal {L}}_{X}T_1)\otimes T_2 + T_1\otimes ({{\mathcal {L}}_{X}^{{\mathbb {C}}{ }}}T_2), \quad \nonumber \\ {\mathcal {L}}_{X}(T_1\otimes \overline{T_2})= & {} {{\mathcal {L}}_{X}^{{\mathbb {C}}{ }}}T_1\otimes \overline{T_2} + T_1\otimes (\overline{{{\mathcal {L}}_{X}^{{\mathbb {C}}{ }}}T_2}). \end{aligned}$$

In the first equation of (2.42), we remark that $T_1$ is a “true” complex-valued tensor, and all others are elements of a product space $V\otimes T_k^l$. We can write the commutators

2.43a $$\begin{aligned}{}[D_\beta , {{\mathcal {L}}_{{\widetilde{X}}}^{{\mathbb {C}}{ }}}]\psi&= i{\textbf{F}}_{\beta {\widetilde{X}}}\psi , \end{aligned}$$

2.43b $$\begin{aligned} \psi&= ig^{\alpha \beta }{\textbf{F}}_{\beta {\widetilde{X}}}\psi + {^{({{\widetilde{X}}})}}\pi ^{\alpha \beta }D_\beta \psi , \end{aligned}$$

2.43c $$\begin{aligned} \eta ^\alpha&= -R^\alpha _{\gamma \alpha \beta }\eta ^\gamma + i{\textbf{F}}_{\alpha \beta }\eta ^\alpha \end{aligned}$$

2.43d $$\begin{aligned} {\widetilde{X}}^\beta [D_\alpha , D_\beta ]\eta ^\alpha - \eta ^\beta [\nabla _\alpha , \nabla _\beta ]{\widetilde{X}}^\alpha&= i{\textbf{F}}_{\alpha \beta }\eta ^\alpha {\widetilde{X}}^\beta \end{aligned}$$

2.43e $$\begin{aligned} \eta ^\alpha&= i{\textbf{F}}_{\alpha \beta }\eta ^\alpha {\widetilde{X}}^\beta - (\nabla _\beta \nabla _\alpha {\widetilde{X}}^\alpha )\eta ^\beta , \end{aligned}$$

2.43f $$\begin{aligned} \eta ^\alpha&= \nabla _\beta (\nabla \cdot {\widetilde{X}})\eta ^\alpha \end{aligned}$$

The identity (2.43a) follows from expanding and using the identity $[D_\alpha , D_\beta ]\phi = i{\textbf{F}}_{\alpha \beta }\phi$ and is a direct analogue of the Cartan formula, (2.43b) follows from writing $D^\alpha = g^{\alpha \beta } D_\beta$, then applying (2.43a) and (2.32), (2.43c) comes from rewriting $D_\alpha = \nabla _\alpha +i{\textbf{A}}_\alpha$ and expanding the commutator, (2.43d) follows from the interchange symmetry of the Riemann curvature tensor, and (2.43e) follows from (2.43d) and straightforward calculation. Combining (2.43b) and (2.43e) gives

2.44 $$\begin{aligned}{}[\Box _g^{{\mathbb {C}}}, D_{{\widetilde{X}}}]\psi= & {} [D_\alpha D^\alpha , {{\mathcal {L}}_{{{\widetilde{X}}}}^{{\mathbb {C}}{ }}}]\psi = iD^\beta ({\textbf{F}}_{\beta {{\widetilde{X}}}}\psi ) + D_\alpha ({^{({{{\widetilde{X}}}})}}\pi ^{\alpha \beta }D_\beta \psi ) \nonumber \\{} & {} + i{\textbf{F}}_{\alpha {{\widetilde{X}}}}D^\alpha \psi - \nabla _\beta (\nabla \cdot {{\widetilde{X}}})D^\beta \psi . \end{aligned}$$

### The Charge Contribution

It follows from elliptic theory that even in the case that $\varvec{\phi }$ is compactly supported, we cannot assume that ${\textbf{F}}$ will decay faster than $r^{-2}$ (to see this, decompose ${\textbf{E}}[{\textbf{F}}]$ into its divergence-free and curl-free components, and consider the potential function of the curl-free part). Fortunately, it was shown in [18] that ${\textbf{F}}$ can be decomposed into ${\textbf{F}}^0+{\textbf{F}}^1$, where ${\textbf{F}}^0$ is explicitly defined and ${\textbf{F}}^1$ decays rapidly. We adapt the consideration there to our class of spacetimes.

We define the charge

2.45 $$\begin{aligned} q[{\textbf{F}}](t) = {\int _{\Sigma _{t}}}\!\! -\sqrt{|g|}{\textbf{J}}^0 dx \end{aligned}$$

Since ${\textbf{J}}$ is divergence-free, we may drop the dependence on time assuming sufficient decay of $\varvec{\phi }\overline{D\varvec{\phi }}$. We define the charge 1-form

2.46 $$\begin{aligned} {\textbf{A}}^{\!q} = \left( \int _0^r \frac{q}{4\pi }\frac{{\overline{\chi }}(s^*-t-2)}{s^{*2}}\, ds^*\right) \,dt, \end{aligned}$$

where $s^* = s + M\chi \ln (s)$. Additionally, ${{\overline{\chi }}}$ is a smooth increasing function satisfying

2.47 $$\begin{aligned} {{\overline{\chi }}}(y) = {\left\{ \begin{array}{ll} 1 &{} y > 1, \\ 0 &{} y < 0, \end{array}\right. } \end{aligned}$$

We can now define ${\textbf{F}}^0 = d{\textbf{A}}^{\!q}$, or

2.48 $$\begin{aligned} {\textbf{F}}^0_{0i} = \omega ^{i}\left( \frac{q}{4\pi }\frac{{{\overline{\chi }}}(r^*-t-2)\partial _r(r^*)}{r^{*2}}\right) . \end{aligned}$$

We take the null decomposition:

2.49a $$\begin{aligned} \rho [{\textbf{F}}^0]&= \left( \frac{q}{4\pi }\frac{{{\overline{\chi }}}(r^*-t-2)}{r^{*2}}\right) , \end{aligned}$$

2.49b $$\begin{aligned} \alpha [{\textbf{F}}^0]&= {\underline{\alpha }}[{\textbf{F}}^0] = \sigma [{\textbf{F}}^0] = 0. \end{aligned}$$

We can use this to establish component estimates on all Lie derivatives of ${\textbf{F}}^0$. Fortunately, our choice of ${\textbf{F}}^0$ makes this process relatively straightforward. We have in particular the estimates

2.50a $$\begin{aligned} |\alpha [{\mathcal {L}}_{{\widetilde{X}}}^I{\textbf{F}}^0]|&\lesssim |q|\lceil {\overline{\chi }}\rceil {\tau _{-}^{}}{\tau _{+}^{-3}}, \end{aligned}$$

2.50b $$\begin{aligned} |\rho [{\mathcal {L}}_{{\widetilde{X}}}^I{\textbf{F}}^0]|&\lesssim |q|\lceil {\overline{\chi }}\rceil {\tau _{+}^{-2}}, \end{aligned}$$

2.50c $$\begin{aligned} |\sigma [{\mathcal {L}}_{{\widetilde{X}}}^I{\textbf{F}}^0]|&\lesssim |q|\lceil {\overline{\chi }}\rceil {\tau _{+}^{-2}}, \end{aligned}$$

2.50d $$\begin{aligned} |{\underline{\alpha }}[{\mathcal {L}}_{{\widetilde{X}}}^I{\textbf{F}}^0]|&\lesssim |q|\lceil {\overline{\chi }}\rceil {\tau _{+}^{-2}}. \end{aligned}$$

These follow from the commutator terms (2.11), using an analogous argument to Lemma (2.1). We put off discussion of the associated current vector until later.

### A Model Morawetz Inequality

Here we prove some model conformal Morawetz-type inequalities, which will motivate features of our energy estimate. We consider the equation

$$\begin{aligned} \partial _\alpha (g^{\alpha \gamma }\partial _\gamma \phi ) = 0 \end{aligned}$$

in Minkowski space, where *g* is close to the Minkowski metric. We take the null frame $\{L = \partial _t+\partial _r, \underline{L} = \partial _t - \partial _r, S_1, S_2\}$, where $S_j$ are piecewise defined orthonormal fields tangent to spheres of fixed radius, and the conformal field

2.51 $$\begin{aligned} K_0 = \tfrac{1}{2}({\tau _{+}^{2}})(\partial _t+\partial _r) + \tfrac{1}{2}({\tau _{-}^{2}})(\partial _t-\partial _r) = (1+t^2+r^2)\partial _t + 2tr\partial _r. \end{aligned}$$

Additionally, we have the optical weights in Minkowski space

$$\begin{aligned} {\tau _{+}^{2}} = 1+(t+r)^2 \quad {\tau _{-}^{2}} = 1+(t-r)^2, \quad {\tau _{0}^{2}} = {\tau _{-}^{2}}/{\tau _{+}^{2}}. \end{aligned}$$

#### The Model Inequality

Our first estimate will show the use of peeling estimates on the metric, as well as give insight as to why additional decay coming from harmonic coordinates is necessary.

##### Theorem 2.5

Let $$H^{\alpha \beta } = g^{\alpha \beta } - m^{\alpha \beta }$$ satisfy

2.52a $$\begin{aligned} {\tau _{+}^{}}|L(H)|_{LL}+{\tau _{-}^{}}|\underline{L}(H)|_{LL}+|H_{LL}|&\le \varepsilon _H{\tau _{0}^{2}}, \end{aligned}$$

2.52b $$\begin{aligned} {\tau _{+}^{}}|L(H)|_{TU}+{\tau _{-}^{}}|\underline{L}(H)|_{TU}+|H_{TU}|&\le \varepsilon _H{\tau _{0}^{}}, \quad T\in \{L, S_1, S_2\}, \quad U\in \{L,\underline{L}, S_1, S_2\} \end{aligned}$$

2.52c $$\begin{aligned} {\tau _{+}^{}}|L(H)|_{\underline{L}\underline{L}}+{\tau _{-}^{}}|\underline{L}(H)|_{\underline{L}\underline{L}}+|H_{\underline{L}\underline{L}}|&\le \varepsilon _H, \end{aligned}$$

for some constant $\varepsilon _H > 0$, where indices are raised and lowered according to the Minkowski metric and

$$\begin{aligned} |X(H)|_{YZ} = |X(H_{\alpha \beta })Y^\alpha Z^\beta |. \end{aligned}$$

There exists a constant $\varepsilon _0$ such that, given a smooth function $\phi$ with compact support, and energy

2.53 $$\begin{aligned} {\mathcal {E}}(t) = {\int _{\Sigma _{t}}}\left( \frac{{\tau _{+}^{2}}}{4}\left( \frac{L(r\phi )}{r}\right) ^2 + \frac{{\tau _{-}^{2}}}{4}\left( \frac{\underline{L}(r\phi )}{r}\right) ^2 + \frac{{\tau _{+}^{2}}+{\tau _{-}^{2}}}{4}\sum _j|S_j\phi |^2\right) \, dx,\nonumber \\ \end{aligned}$$

the estimate

2.54 $$\begin{aligned} {\mathcal {E}}(T) \lesssim {\mathcal {E}}(0) + \varepsilon _H\int _0^T\frac{{\mathcal {E}}(t)}{1+t} + \int _0^T{\int _{\Sigma _{t}}}\left| \frac{K_0(r\phi )}{r}\partial _\alpha (g^{\alpha \gamma }\partial _\gamma \phi )\right| \end{aligned}$$

for all $$\varepsilon _H < \varepsilon _0$$.

##### Remark 2.6

This is a standard conformal energy estimate on a perturbed background spacetime and implies slowly growing conformal energy, as seen in, e.g., [10]. We briefly remark on the metric assumptions (2.52). We expect the $H_{LL}$ component to decay more rapidly along the light cone than other components, which is heuristically consistent with the harmonic coordinate condition. However, under examination of (2.18), (2.24), one sees that neither $H^0_{LL}$ nor $H^1_{LL}$ satisfy the bounds (2.52). Therefore, while this toy model demonstrates the utility of peeling estimates on metric components, a more delicate analysis will be necessary for the full system.

##### Proof

Our main tool is the divergence theorem applied to the quantity

2.55 $$\begin{aligned} P^\alpha = -\left( \tfrac{K_0(r\phi )}{r}g^{\alpha \gamma }\partial _\gamma \phi - \tfrac{1}{2} K_0^\alpha g^{\gamma \delta }\partial _\gamma \phi \partial _\delta \phi + \tfrac{1}{2}(L^\alpha +\underline{L}^\alpha )\phi ^2\right) . \end{aligned}$$

The field $K_0$ is conformal Killing (but not Killing) with respect to the Minkowski metric. Integrating along time slices gives

2.56 $$\begin{aligned} E(t)&= {\int _{\Sigma _{t}}}P^0 = {\int _{\Sigma _{t}}}\left( \tfrac{K_0(r\phi )}{r}\partial _t\phi + \tfrac{{\tau _{+}^{2}}+{\tau _{-}^{2}}}{4}(-L\phi \underline{L}\phi + |S_j\phi |^2) - \phi ^2\right) \nonumber \\&\quad + \left( -\tfrac{K_0(r\phi )}{r}(H^{L\gamma }\partial _\gamma \phi + H^{\underline{L}\gamma }\partial _\gamma \phi )+ \tfrac{1}{4}({\tau _{+}^{2}}+{\tau _{-}^{2}})H^{\gamma \delta }\partial _\gamma \phi \partial _\delta \phi \right) , \end{aligned}$$

Therefore,

2.57 $$\begin{aligned} E(T)-E(0) = \int _0^T{\int _{\Sigma _{t}}}\partial _\alpha P^\alpha \, dx \, dt. \end{aligned}$$

We must show that *E* and ${\mathcal {E}}$ are equivalent and that the integral on the right-hand side is bounded by the right-hand side of (2.54) up to a constant. Writing

$$\begin{aligned} \partial _t\phi = \tfrac{1}{2}\left( \tfrac{L(r\phi )}{r} + \tfrac{\underline{L}(r\phi )}{r}\right) , \quad -L\phi \underline{L}\phi = -\left( \tfrac{L(r\phi )}{r}\right) \left( \tfrac{\underline{L}(r\phi )}{r}\right) - \tfrac{2\partial _r(r\phi )}{r}\tfrac{\phi }{r} + \left( \tfrac{\phi }{r}\right) ^2 \end{aligned}$$

so the first line of (2.56) is equivalent to

2.58 $$\begin{aligned}{} & {} {\int _{\Sigma _{t}}}\Big (\tfrac{{\tau _{+}^{2}}}{4}\left( \tfrac{L(r\phi )}{r}\right) + \tfrac{{\tau _{-}^{2}}}{4}\left( \tfrac{\underline{L}(r\phi )}{r}\right) \nonumber \\{} & {} \quad + \tfrac{{\tau _{+}^{2}}+{\tau _{-}^{2}}}{4}\left( \sum _j|S_j\phi |^2 - \tfrac{2\partial _r(r\phi )}{r}\tfrac{\phi }{r} + \left( \tfrac{\phi }{r}\right) ^2\Big ) - \phi ^2\right) . \end{aligned}$$

Adding the spatial divergence

2.59 $$\begin{aligned} \partial _i\left[ \left( \tfrac{{\tau _{+}^{2}}+{\tau _{-}^{2}}}{4}\tfrac{\omega _i\phi ^2}{r}\right) \right] = \tfrac{{\tau _{+}^{2}}+{\tau _{-}^{2}}}{4}\tfrac{\phi ^2}{r^2} + \tfrac{{\tau _{+}^{2}}+{\tau _{-}^{2}}}{4}\tfrac{2\phi \partial _r\phi }{r} + \phi ^2. \end{aligned}$$

and expanding $r\partial _r\phi = \partial _r(r\phi )-\phi$ gives $\mathcal {{E}}(t)$.

Now we consider the terms in (2.56) containing *H*, for which we will use the estimates (2.52). It suffices to show that the error terms can be bounded uniformly by $\frac{1}{2}{\mathcal {E}}(t)$. We show this for the terms containing $H^{\gamma \delta }\partial _\gamma \phi \partial _\delta \phi$. Other terms follow similarly. Writing $|{{\overline{\partial }}}\phi |^2 = |L\phi |^2 + |S_1\phi |^2 + |S_1\phi |^2$, then

2.60 $$\begin{aligned} |H^{\gamma \delta }\partial _\gamma \phi \partial _\delta \phi |{} & {} \lesssim \varepsilon _H{\tau _{0}^{2}}|\underline{L}\phi |^2 + \varepsilon _H{\tau _{0}^{}}|\underline{L}\phi ||{{\overline{\partial }}}\phi | + \varepsilon _H|{{\overline{\partial }}}\phi |^2 \nonumber \\{} & {} \lesssim \varepsilon _H{\tau _{0}^{2}}|\underline{L}\phi |^2 + \varepsilon _H|{{\overline{\partial }}}\phi |^2, \end{aligned}$$

so Lemma 10.6 implies

2.61 $$\begin{aligned} {\int _{\Sigma _{t}}}{\tau _{+}^{2}}|L(\phi )|^2 + {\tau _{-}^{2}}|\underline{L}(\phi )|^2 + {\tau _{+}^{2}}\Big |\tfrac{\phi }{r}\Big |^2\, dx \lesssim {\mathcal {E}}(t), \end{aligned}$$

so

2.62 $$\begin{aligned} {\int _{\Sigma _{t}}} \frac{1}{4}({\tau _{-}^{2}}+{\tau _{+}^{2}})|H^{\gamma \delta }\partial _\gamma \phi \partial _\delta \phi |{} & {} \lesssim {\int _{\Sigma _{t}}} \varepsilon _H{\tau _{0}^{2}}{\tau _{+}^{2}}|\underline{L}\phi |^2 + \varepsilon _H{\tau _{+}^{2}}|{{\overline{\partial }}}\phi |^2\nonumber \\{} & {} \lesssim \varepsilon _H{\mathcal {E}}(t). \end{aligned}$$

The quantity on the right is bounded by $\frac{1}{2}{\mathcal {E}}(t)$ for sufficiently small $\varepsilon _H$. We consider the divergence

2.63 $$\begin{aligned} \partial _\alpha P^\alpha&= -\tfrac{K_0(r\phi )}{r}\partial _\alpha (g^{\alpha \beta }\partial _\beta \phi ) - g^{\alpha \beta }\partial _\alpha \Big (\tfrac{K_0(r\phi )}{r}\Big )\partial _\beta \phi \nonumber \\&\quad + \tfrac{1}{2} K_0(g^{\alpha \beta })\partial _\alpha \phi \partial _\beta \phi + \tfrac{1}{2}(\partial _\alpha K_0^\alpha )(g^{\beta \gamma }\partial _\beta \phi \partial _\gamma \phi ) \nonumber \\&\quad + g^{\alpha \beta }K_0(\partial _\alpha \phi )\partial _\beta \phi - 2\phi \partial _t\phi . \end{aligned}$$

To bound this, we start with the identities

$$\begin{aligned} \tfrac{K_0(r\phi )}{r} = K_0(\phi ) + 2t\phi , \quad \partial _\alpha K_0^\alpha = 8t, \quad \frac{1}{2} {\mathcal {L}}_{K_0}(m^{-1}) = -2tm^{-1}. \end{aligned}$$

Then,

$$\begin{aligned} \partial _\alpha \Big (\tfrac{K_0(r\phi )}{r}\Big ) = \partial _\alpha K_0\phi + \partial _\alpha (2t\phi ) = [\partial _\alpha , K_0]\phi + K_0(\partial _\alpha \phi ) + \partial _\alpha (2t\phi ), \end{aligned}$$

so

$$\begin{aligned} - g^{\alpha \beta }\partial _\alpha \Big (\tfrac{K_0(r\phi )}{r}\Big )\partial _\beta \phi +g^{\alpha \beta }K_0(\partial _\alpha \phi )\partial _\beta \phi = -g^{\alpha \beta }[\partial _\alpha , K_0]\phi \partial _\beta \phi - g^{\alpha \beta }\partial _\alpha (2t\phi )\partial _\beta \phi . \end{aligned}$$

Equation (2.63) can thus be rewritten as:

$$\begin{aligned} \partial _\alpha P^\alpha&= -\tfrac{K_0(r\phi )}{r}\partial _\alpha (g^{\alpha \beta }\partial _\beta \phi ) + \tfrac{1}{2} ({\mathcal {L}}_{K_0} H)^{\alpha \beta }\partial _\alpha \phi \partial _\beta \phi + 2t(H^{\beta \gamma }\partial _\beta \phi \partial _\gamma \phi ) - 2H^{0\beta }\phi \partial _\beta \phi . \end{aligned}$$

as well as the null decomposition

2.64 $$\begin{aligned} L^{\alpha }[\partial _\alpha , K_0]= 2(t+r)L\phi , \quad \underline{L}^{\alpha }[\partial _\alpha , K_0] = 2(t-r)\underline{L}\phi , \quad S_j^\alpha [\partial _\alpha , K_0] = 0,\nonumber \\ \end{aligned}$$

then, from the bounds (2.52), (2.60), a null decomposition, and the estimate $t < {\tau _{+}^{}}$ we can say

2.65a $$\begin{aligned} | 2t(H^{\beta \gamma }\partial _\beta \phi \partial _\gamma \phi )|&\lesssim \varepsilon _H{\tau _{-}^{2}}{\tau _{+}^{-1}}|\underline{L}\phi |^2 + \varepsilon _H{\tau _{+}^{}}|{{\overline{\partial }}}\phi |^2 \end{aligned}$$

2.65b $$\begin{aligned} |H^{0\beta }\phi \partial _\beta \phi |&\lesssim \varepsilon _H{\tau _{0}^{}}|\phi ||\underline{L}\phi | + \varepsilon _H|\phi ||{{\overline{\partial }}}\phi | \lesssim \varepsilon _H({\tau _{+}^{-1}}|\phi |^2 + {\tau _{+}^{}}|{{\overline{\partial }}}\phi |^2 + {\tau _{-}^{2}}{\tau _{+}^{-1}}|\underline{L}\phi |^2)\end{aligned}$$

2.65c $$\begin{aligned} |H^{\alpha \beta }[\partial _\alpha , K_0]\phi \partial _\beta \phi |&\lesssim \varepsilon _H{\tau _{+}^{}}(|{{\overline{\partial }}}\phi |^2 + {\tau _{0}^{}}|{{\overline{\partial }}}\phi ||\underline{L}\phi | +{\tau _{0}^{2}}|\underline{L}\phi |^2) \end{aligned}$$

2.65d $$\begin{aligned} |K_0(H^{\alpha \beta })\partial _\alpha \phi \partial _\beta \phi |&\lesssim \varepsilon _H{\tau _{+}^{}}(|{{\overline{\partial }}}\phi |^2 + {\tau _{0}^{}}|{{\overline{\partial }}}\phi ||\underline{L}\phi | +{\tau _{0}^{2}}|\underline{L}\phi |^2). \end{aligned}$$

Noting cancellations in (2.63) and bounding the integral of each term in (2.65) in space by $(1+t)^{-1}{\mathcal {E}}(t)$ completes the proof. $\square$

#### The Modified Inequalities

The bound (2.54) in itself is not particularly helpful for two reasons. First, an application of Gronwall’s lemma to the bound (2.54) gives slowly growing energy, which is not enough to close the results of Sect. 8. Second, in harmonic coordinates we cannot expect $H_{LL}$ to decay faster than $t^{-1}$ along the light cone, due to the presence of a nonzero ADM mass.

We approach this problem from both sides. First, in order to improve the bound on $H_{LL}$, we use a modified null frame which gives us improved decay of $|g_{LL}|$ along the (modified) light cone, see (2.22) and (2.24). We choose a frame which accounts for the presence of the ADM mass, such that $g_{LL}$ decays like $\langle r \rangle ^{-1-\alpha }$ at time 0, for some constant $\alpha > 0$. We then note that the harmonic coordinate condition $\Gamma _{{{\widetilde{L}}}} = 0$ can be treated as a transport equation for $g_{{{\widetilde{L}}}{{\widetilde{L}}}}$ along the flow lines of ${\underline{{\widetilde{L}}}}$. (Full details may be found in the sequel [11]). The second step, which we detail later, will be the use of a fractional Morawetz estimate.

We now verify that modified metric indeed gives an improved estimate. We define

2.66 $$\begin{aligned} \widetilde{K} = (1+{\underline{u}^{*{}}}^2){{\widetilde{L}}}+ (1+{u^{*{}}}^2){\underline{{\widetilde{L}}}}\end{aligned}$$

##### Lemma 2.7

Given the inverse metric $m_0^{\alpha \beta } = m^{\alpha \beta } + H_0^{\alpha \beta }$, and the energyit follows that for sufficiently small *M*,

2.67 $$\begin{aligned} E_0[\phi ](T) - E_0[\phi ](0) \lesssim M\int _0^T\tfrac{E_0[\phi ](t)}{1+t} + \int _0^T {\int _{\Sigma _{t}}} \left| \tfrac{K_0^*(r^*\phi )}{r^*}{\widetilde{\partial }}_\alpha ({\widetilde{m}}_0^{\alpha \beta }{\widetilde{\partial }}_\beta \phi ) \right| ,\nonumber \\ \end{aligned}$$

where ${\widetilde{m}}_0$ is $m+H_0$ expressed in the $\widetilde{x}^\alpha$ coordinates. Alternatively, for any $c, {\overline{\mu }}> 0$,

2.68 $$\begin{aligned} E_0[\phi ](T) - E_0[\phi ](0)\lesssim & {} M\!\int _0^T\tfrac{E_0[\phi ](t)}{1+t} + c\int _0^T\!{\int _{\Sigma _{t}}}{\tau _{-}^{-1-2{\overline{\mu }}}}{\tau _{+}^{2}}\Big |\tfrac{{{\widetilde{L}}}(r^*\!\phi )}{r^*}\Big |^2\nonumber \\{} & {} + \tfrac{1}{c}\int _0^T \!{\int _{\Sigma _{t}}}{\tau _{+}^{2}}{\tau _{-}^{1+2{\overline{\mu }}}} \left| {\widetilde{\partial }}_\alpha ({\widetilde{m}}_0^{\alpha \beta }{\widetilde{\partial }}_\beta \phi ) \right| ^2. \end{aligned}$$

##### Proof

The estimate (2.67) follows almost directly from Theorem 2.5 using the bounds (2.23) (with the assumption $M\le \varepsilon _H$). We must additionally supplement (2.23) with the bound

2.69 $$\begin{aligned} |{\mathcal {L}}_{K_0}m_0^{\alpha \beta }\partial _\alpha \phi \partial _\beta \phi |\lesssim M{\tau _{+}^{}}(|{{\overline{\partial }}}\phi |^2 + {\tau _{0}^{}}|{{\overline{\partial }}}\phi ||\underline{L}\phi | +{\tau _{0}^{2}}|\underline{L}\phi |^2), \end{aligned}$$

which follows from a careful null decomposition along with the expansion (2.4).

(2.68) follows from the Cauchy–Schwartz inequality. $\square$

##### Remark 2.8

We could more directly prove a slightly weaker version of this result using only the bounds (2.23), which would introduce a factor of $\langle \ln (1+t)\rangle ^2$ in the integrated energy estimate. Our later use of the fractional estimate will in essence provide an additional power of $\langle t\rangle ^{2s-2}$ for these terms, which would allow us to close our argument even with the weaker bounds.

In (2.67), we have slowly growing energy even when ${\widetilde{\partial }}_\alpha ({\widetilde{m}}_0^{\alpha \beta }{\widetilde{\partial }}_\beta \phi )$ vanishes, due to error terms arising from the curvature of $m_0$. In particular, (2.65d) contains a term like $|({\mathcal {L}}_{K_0}H)_{LL}||\underline{L}\phi |^2$, and in null coordinates ${\underline{u}^{*{2}}}{{\widetilde{L}}}H_{LL}$ cannot be assumed to decay faster than $t^{-1}$ (one could obtain stronger decay using a double null foliation, but this of course introduces additional difficulties elsewhere). We thus motivate our use of the fractional field $\widetilde{K^s}{}$ used by Lindblad and Sterbenz in [18]. Using the fractional weight will allow us to multiply the weights on the right-hand side of (2.65d) by ${\tau _{+}^{2s-2}}$. Consequently, for $s<1$, the $|\underline{L}\phi |^2$ terms appearing in the energy on the right-hand side of (2.67) appear with an improved weight, for which Gronwall’s lemma would give global energy bounds.

This allows us to close our argument with the weaker assumption $|g_{LL}| \lesssim \epsilon {\tau _{0}^{2s}}{\tau _{+}^{-\delta }}$ for some $s>\frac{1}{2}, \delta > 0$, with analogous weaker assumptions for other components and derivatives of *H*.

Additionally, for the Maxwell–Klein–Gordon system, bounding the second term on the right of (2.68) is nontrivial. By introducing a weight *w* which grows in $r^* - t$, we can introduce a spacetime term to our energy. We state (without proof) another model estimate, which follows directly from multiplying $P^\alpha$ by *w* and noting that $\nabla w$ is approximately null.

##### Lemma 2.9

Given the inverse metric $m_0$, and the energyalong with the interior spacetime energywhere

$$\begin{aligned} w= & {} w(r^* - t) = {\left\{ \begin{array}{ll} 1+(1+(t-r^*))^{-2{\overline{\mu }}} &{} r^*< t, \\ 1+(1+(r^*-t))^{1+2\gamma } &{} r^*> t, \end{array}\right. } \quad \quad \\ w'\approx & {} {\left\{ \begin{array}{ll} (1+(t-r^*))^{-1-2{\overline{\mu }}} &{} r^* < t, \\ 1+(1+(r^*-t))^{2\gamma } &{} r^* > t, \end{array}\right. } \end{aligned}$$

for some constants ${\overline{\mu }}, \gamma > 0$, we have the estimate

2.70 $$\begin{aligned} E_w[\phi ](T) + S_w[\phi ](T)\lesssim & {} E_w[\phi ](0) + M\int _0^T\frac{E_w[\phi ](t)\langle \ln (1+t)\rangle ^2}{1+t}\nonumber \\{} & {} + \int _0^T {\int _{\Sigma _{t}}} \left| \frac{K_0^*(r^*\phi )}{r^*}{\widetilde{\partial }}_\alpha ({\widetilde{m}}_0^{\alpha \beta }{\widetilde{\partial }}_\beta \phi ) w\right| . \end{aligned}$$

## $L^2$ Estimates for ${\textbf{F}}$

In this section, we prove an energy estimate, which we will use to bound certain quantities derived from ${\textbf{F}}$. Our basic approach here follows the fractional Morawetz estimate used in [18], with the substitution of the modified vector fields, and with additional calculations taken in order to bound the error terms.

### Structure of the Estimate

We first define the field

3.1 $$\begin{aligned} \widetilde{K^s}{}= \frac{1}{2}\left( (1+{\underline{u}^{*{2s}}}){{\widetilde{L}}}+ (1+{|u^{*}|^{2s}}){\underline{{\widetilde{L}}}}\right) \end{aligned}$$

for $1/2< s< (1+\gamma - 4\delta )/2 < 1$, which, for $r^* \le t$, can be seen as an interpolation between the fields $\widetilde{Z} = \tfrac{1}{2}({\underline{u}^{*{}}}{{\widetilde{L}}}+ {u^{*{}}}{\underline{{\widetilde{L}}}})$ and $\widetilde{K}_0 = \frac{1}{2}({\underline{u}^{*{2}}}{{\widetilde{L}}}+ {u^{*{2}}}{\underline{{\widetilde{L}}}})$, which correspond to conformal Killing fields in Minkowski space. We add the field ${\widetilde{\partial }}_t$ in order to ensure that we have a timelike field close to the light cone.

Our core energy estimate will follow from the divergence theorem applied to a quantity $- T[{\textbf{G}}]_{\alpha \beta }\widetilde{K^s}{}^\alpha w$. This does not apply directly to our field ${\textbf{F}}$, since the consequent weighted energy is finite only if the charge is 0. We will instead take the charge decomposition given in Sect. 2.5, and use this on ${\textbf{F}}^1$ and its derivatives.

### Weights and Notation

Before we arrive at the statement for the basic estimate, we define the weights:

3.2a $$\begin{aligned} w&= {\tau _{-}^{2(s_0-s)}}{{\overline{\chi }}}(-{u^{*{}}}) + (1-{\overline{\chi }}(-{u^{*{}}})), \end{aligned}$$

3.2b $$\begin{aligned} {\widetilde{w}}&= (1 + (2-{u^{*{}}})^{2(s_0-s)}){\overline{\chi }}(-{u^{*{}}}) + (1+(2+{u^{*{}}})^{-2{{\overline{\mu }}}})(1-{{\overline{\chi }}}(-{u^{*{}}})) \nonumber \\&\quad + (1+{\underline{u}^{*{}}})^{-2{{\overline{\mu }}}}\left( (2-{u^{*{}}})^{2(s_0-s) + 2{{\overline{\mu }}}}{\overline{\chi }}(-{u^{*{}}}) + 1 - {\overline{\chi }}(-{u^{*{}}})\right) , \end{aligned}$$

3.2c $$\begin{aligned} w_{{\overline{\mu }}}&= {\tau _{-}^{2(s_0-s)}}{{\overline{\chi }}}(-{u^{*{}}}) + {\tau _{-}^{2{{\overline{\mu }}}}}(1-{\overline{\chi }}(-{u^{*{}}})), \end{aligned}$$

3.2d $$\begin{aligned} w'&= {\tau _{-}^{2(s_0-s) - 1}}{{\overline{\chi }}}(-{u^{*{}}}) + {\tau _{-}^{-1-2{{\overline{\mu }}}}}(1-{\overline{\chi }}(-{u^{*{}}})). \end{aligned}$$

We recall the assumption that $s_0 < 3/2$, and $0< 2{{\overline{\mu }}} < s-1/2$. Here ${{\overline{\chi }}}$ is the same as in Eq. (2.47).

We briefly discuss these weights, which are adapted from weights in [18]. Our energy estimate will be conducted with respect to the weight ${\widetilde{w}}$, which satisfies the following properties:

3.3 $$\begin{aligned} {\widetilde{w}} \approx w, \quad -\frac{1}{2}{\underline{{\widetilde{L}}}}({\widetilde{w}}) \approx w', \quad -\frac{1}{2}{{\widetilde{L}}}({\widetilde{w}}) \approx {\tau _{0}^{1+2{{\overline{\mu }}}}}w'. \end{aligned}$$

Note that $w'$ describes the derivative of ${\widetilde{w}}$, and we never differentiate *w* directly. Additionally, $w_{{\overline{\mu }}}$ satisfies

$$\begin{aligned} {\tau _{-}^{}}w' w_{{\overline{\mu }}} \approx w^2, \end{aligned}$$

which will be used in our current norm. We also define

$$\begin{aligned} \tau _w = {\left\{ \begin{array}{ll} {\tau _{-}^{1+2{{\overline{\mu }}}}} &{} r^* \le t, \\ {\tau _{-}^{}}&{} r^* > t. \end{array}\right. } \end{aligned}$$

We motivate this by noting

$$\begin{aligned} \tau _w^{-1} \approx \frac{w'}{w}, \quad {\tau _{-}^{-1-2{{\overline{\mu }}}}}\le \tau _w^{-1} \le {\tau _{-}^{-1}}. \end{aligned}$$

Recalling the definitions (1.34) and (1.35), we also define

3.4a $$\begin{aligned} |{\overline{{\textbf{G}}}}|^2&= |{\alpha }[{\textbf{G}}]|^2 + |\rho [{\textbf{G}}]|^2 + |\sigma [{\textbf{G}}]|^2, \end{aligned}$$

3.4b $$\begin{aligned} |{\textbf{G}}_N|^2&= |\alpha [{\textbf{G}}]|^2 + |\rho [{\textbf{G}}]|^2 + |\sigma [{\textbf{G}}]|^2 + |\alpha [{\textbf{G}}]||{\underline{\alpha }}[{\textbf{G}}]|, \end{aligned}$$

3.4c $$\begin{aligned} |{\textbf{G}}|^2&= |{\alpha }[{\textbf{G}}]|^2 + |\rho [{\textbf{G}}]|^2 + |\sigma [{\textbf{G}}]|^2 + |{\underline{\alpha }}[{\textbf{G}}]|^2, \end{aligned}$$

The components in $|{\overline{{\textbf{G}}}}|^2$ are analogous to derivatives of a scalar field $\phi$ along the light cone, and $|{\textbf{G}}_N|^2$ is analogous to a form satisfying the null condition. Likewise, we can define the current associated with ${\textbf{G}}$,

3.5 $$\begin{aligned} {\textbf{J}}[{\textbf{G}}]_\alpha = \nabla ^\beta {\textbf{G}}_{\alpha \beta }, \end{aligned}$$

and the spacetime weighted current $L^2$ norm

3.6 $$\begin{aligned} \left\Vert {\textbf{J}}\right\Vert _{L^2[w]}= & {} \left\Vert {\tau _{+}^{s}}{\tau _{0}^{-1/2-{{\overline{\mu }}}}}{\tau _{-}^{1/2}} {\textbf{J}}^{{\underline{{\widetilde{L}}}}} w^{1/2}_{{\overline{\mu }}}\right\Vert _2 + \left\Vert {\tau _{+}^{s}}{\tau _{-}^{1/2}}|{\textbf{J}}^{{{\widetilde{S}}_{i}}}|w_{{\overline{\mu }}}^{1/2}\right\Vert _2\nonumber \\{} & {} + \left\Vert {\tau _{0}^{s-1/2-{{\overline{\mu }}}}}{\tau _{-}^{s+1/2}}|{\textbf{J}}^{{{\widetilde{L}}}}| w_{{\overline{\mu }}}^{1/2}\right\Vert _2. \end{aligned}$$

We define also the energies we will use in our theorem. For any two-form ${\textbf{G}}_{\alpha \beta }$ defined on $[0,T] \times {\mathbb {R}}^3$, we define the time-slice energy

3.7 $$\begin{aligned} E_0[{\textbf{G}}](T) = \sup _{0 \le t \le T}{\int _{\Sigma _{t}}}\left( {\tau _{+}^{2s}}\left( |{\alpha }|^2 + |\rho |^2 + |\sigma |^2\right) + {\tau _{-}^{2s}}|{\underline{\alpha }}|^2\right) w \, dx, \end{aligned}$$

the spacetime energy

3.8 $$\begin{aligned} S_0[{\textbf{G}}](T)= & {} \int _0^T{\int _{\Sigma _{t}}} \left( {\tau _{+}^{2s}}|{\alpha }|^2\right. \nonumber \\{} & {} \left. + {\tau _{0}^{1+2{{\overline{\mu }}}}}\left( {\tau _{+}^{2s}}(|\rho |^2 + |\sigma |^2) + {\tau _{-}^{2s}}|{\underline{\alpha }}|^2\right) \right) w' \, dx \, dt, \end{aligned}$$

and, defining the region $C_{U, T}$ to be the subset of $[0,T]\times {\mathbb {R}}^3$ where ${u^{*{}}}= U$, the weak conical energy

3.9 $$\begin{aligned} C_0[{\textbf{G}}](T) = \sup _{U}\int _{C_{U, T}}-\left( {\tau _{+}^{2s}}\,T[{\textbf{G}}]({{\widetilde{L}}}, \nabla {u^{*{}}}) + {\tau _{-}^{2s}}\,T[{\textbf{G}}]({\underline{{\widetilde{L}}}}, \nabla {u^{*{}}})\right) w \, dV_C,\nonumber \\ \end{aligned}$$

where $T[{\textbf{G}}]$ is defined in (1.17b). We consequently define the weighted energy of a 2-form with zero charge,

3.10 $$\begin{aligned} {\mathcal {E}}_0[{\textbf{G}}](T) = E_0[{\textbf{G}}](T) + S_0[{\textbf{G}}](T) + C_0[{\textbf{G}}](T), \end{aligned}$$

as well as the iterated energy

3.11 $$\begin{aligned} {\mathcal {E}}_k[{\textbf{G}}](T) = \sum _{\begin{array}{c} |I| \le k \end{array}} {\mathcal {E}}_0[{\mathcal {L}}_{{\widetilde{X}}}^I{\textbf{G}}](T). \end{aligned}$$

#### Theorem 3.1

Let ${\textbf{G}}$ be a 2-form defined on $[0,T]\times {\mathbb {R}}^3$, which satisfies $\nabla ^\beta {\textbf{G}}_{\alpha \beta } = {\textbf{J}}$. Then, ${\textbf{G}}$ satisfies the estimate

3.12 $$\begin{aligned} E_0[{\textbf{G}}](T) + S_0[{\textbf{G}}](T) + C_0[{\textbf{G}}](T) \lesssim E_0[{\textbf{G}}](0) + \left\Vert {\textbf{J}}[{\textbf{G}}]\right\Vert ^2_{L^2[w]}. \end{aligned}$$

provided that the quantities on the right-hand side are bounded.

#### Remark 3.2

We say $C_0$ is weak because it is not positive definite. In particular, we do not know that the vectors ${{\widetilde{L}}}, \nabla u$ are causal, so $C_0$ could have an (unsigned) error of size $\varepsilon _g|{\underline{\alpha }}|^2$. Later we will bound this error by higher-order energies using weighted Klainerman–Sobolev-type inequalities applied to the energy $E_0$. For now, we note that $C_0 \ge 0$ by taking $U=T$ in the definition.

#### Proof

We take

3.13 $$\begin{aligned} P[{\textbf{G}}]_\alpha = -T[{\textbf{G}}]_{\alpha \beta }\widetilde{K^s}{}^\beta {\widetilde{w}}. \end{aligned}$$

which has divergence

3.14 $$\begin{aligned} \nabla ^\alpha P[{\textbf{G}}]_\alpha = {\textbf{G}}_{\beta \gamma }\widetilde{K^s}{}^\beta J[{\textbf{G}}]^\gamma {\widetilde{w}} - T[{\textbf{G}}]_{\alpha \beta }\widetilde{K^s}{}^{\beta ;\alpha }{\widetilde{w}} - T[{\textbf{G}}]_{\alpha \beta }\widetilde{K^s}{}^\beta \nabla ^\alpha ({\widetilde{w}}).\nonumber \\ \end{aligned}$$

We apply the divergence theorem on $[0,t]\times {\mathbb {R}}^3$ (with $t\in [0,T]$) to get

3.15 $$\begin{aligned} {\int _{\Sigma _{T}}}-P[{\textbf{G}}]^0 + {\int _{\Sigma _{0}}}P[{\textbf{G}}]^0= & {} \int _0^t {\int _{\Sigma _{t}}}\left( {\textbf{G}}(\widetilde{K^s}{}, {\textbf{J}}[{\textbf{G}}]) {\widetilde{w}} \right. \nonumber \\{} & {} \left. - T[{\textbf{G}}]_{\alpha \beta }\widetilde{K^s}{}^{\beta ;\alpha }{\widetilde{w}} - T[{\textbf{G}}]_{\alpha \beta }\widetilde{K^s}{}^\beta \nabla ^\alpha ({\widetilde{w}})\right) ,\nonumber \\ \end{aligned}$$

where all integrals are evaluated with respect to the volume element of *g*.

We briefly detail our strategy. The left-hand side will give $E_0(0)$ and $E_0(T)$. We will bound the term containing $({\textbf{G}}(\widetilde{K^s}{}, {\textbf{J}})$ using our current norm combined with Hölder’s inequality. The approximate conformality of $\widetilde{K^s}{}$ will allow us to discard the term containing $Q[{\textbf{G}}]_{\alpha \beta }\widetilde{K^s}{}^{\beta ;\alpha }$. The term containing $\nabla {\widetilde{w}}$ will give $S_0$. Additionally, we may repeat this estimate over regions exterior to the cone ${u^{*{}}}= c$ to get $C_0$.

Recalling the identity (2.29), we have the commutators

3.16 $$\begin{aligned} {[}\widetilde{K^s}{}, {{\widetilde{L}}}]= & {} -\tfrac{1}{2}{{\widetilde{L}}}({\underline{u}^{*{2s}}}){{\widetilde{L}}}, \quad [\widetilde{K^s}{}, {\underline{{\widetilde{L}}}}] = -\tfrac{1}{2}{\underline{{\widetilde{L}}}}({|u^{*}|^{2s}}){\underline{{\widetilde{L}}}}, \quad \nonumber \\ {[}\widetilde{K^s}{}, {{\widetilde{S}}_{j}}]= & {} -\tfrac{1}{2}\tfrac{{\underline{u}^{*{2s}}} - {|u^{*}|^{2s}}}{r^*}{{\widetilde{S}}_{j}}. \end{aligned}$$

The identity (2.14) gives the null decomposition

3.17a $$\begin{aligned} ({\mathcal {L}}_{\widetilde{K^s}{}}g)_{{{\widetilde{L}}}{{\widetilde{L}}}}&= \widetilde{K^s}{}(g_{{{\widetilde{L}}}{{\widetilde{L}}}}) + {{\widetilde{L}}}({\underline{u}^{*{2s}}})g_{{{\widetilde{L}}}{{\widetilde{L}}}}, \end{aligned}$$

3.17b $$\begin{aligned} ({\mathcal {L}}_{\widetilde{K^s}{}}g)_{{\underline{{\widetilde{L}}}}{\underline{{\widetilde{L}}}}}&= \widetilde{K^s}{}(g_{{\underline{{\widetilde{L}}}}{\underline{{\widetilde{L}}}}}) + {\underline{{\widetilde{L}}}}({u^{*{2s}}})g_{{\underline{{\widetilde{L}}}}{\underline{{\widetilde{L}}}}}, \end{aligned}$$

3.17c $$\begin{aligned} ({\mathcal {L}}_{\widetilde{K^s}{}}g)_{{{\widetilde{L}}}{\underline{{\widetilde{L}}}}}&= \widetilde{K^s}{}(g_{{\underline{{\widetilde{L}}}}{{\widetilde{L}}}}) + 2s({\underline{u}^{*{2s-1}}} + \text {sgn}({u^{*{}}}){|u^{*}|^{2s-1}})(g_{{\underline{{\widetilde{L}}}}{{\widetilde{L}}}}), \end{aligned}$$

3.17d $$\begin{aligned} ({\mathcal {L}}_{\widetilde{K^s}{}}g)_{{{\widetilde{L}}}{{\widetilde{S}}_{j}}}&= \widetilde{K^s}{}(g_{{{\widetilde{L}}}{{\widetilde{S}}_{j}}}) + \left( 2s{\underline{u}^{*{2s-1}}} + \tfrac{1}{2}\tfrac{{\underline{u}^{*{2s}}} - {|u^{*}|^{2s}}}{r^*}\right) g_{{{\widetilde{L}}}{{\widetilde{S}}_{j}}}, \end{aligned}$$

3.17e $$\begin{aligned} ({\mathcal {L}}_{\widetilde{K^s}{}}g)_{{\underline{{\widetilde{L}}}}{{\widetilde{S}}_{j}}}&= \widetilde{K^s}{}(g_{{\underline{{\widetilde{L}}}}{{\widetilde{S}}_{j}}}) + \left( 2s\cdot \text {sgn}({u^{*{}}}){|u^{*}|^{2s-1}} + \tfrac{1}{2}\tfrac{{\underline{u}^{*{2s}}} - {|u^{*}|^{2s}}}{r^*}\right) g_{{\underline{{\widetilde{L}}}}{{\widetilde{S}}_{j}}}, \end{aligned}$$

3.17f $$\begin{aligned} ({\mathcal {L}}_{\widetilde{K^s}{}}g)_{{{\widetilde{S}}_{i}}{{\widetilde{S}}_{j}}}&= \widetilde{K^s}{}(g_{{{\widetilde{S}}_{i}}{{\widetilde{S}}_{j}}}) + \tfrac{{\underline{u}^{*{2s}}} - {|u^{*}|^{2s}}}{r^*}g_{{{\widetilde{S}}_{i}}{{\widetilde{S}}_{j}}}, \end{aligned}$$

with other components following from symmetry. Additionally,

3.18 $$\begin{aligned} \tfrac{{\underline{u}^{*{2s}}} - {|u^{*}|^{2s}}}{r^*}= & {} t^{2s-1}\tfrac{(1+r^*/t)^{2s} - |1-r^*/t|^{2s}}{r^*/t} \nonumber \\= & {} r^{*2s-1}((1+t/{r^*})^{2s} - |1-t/r^*|^{2s}), \end{aligned}$$

and take the $t^{2\,s-1}$ and $r^{*2\,s-1}$ terms for $r^*/t \rightarrow 0$, $t/r^* \rightarrow 0$, respectively, which gives us

$$\begin{aligned} \tfrac{1}{2}\tfrac{{\underline{u}^{*{2s}}} - {|u^{*}|^{2s}}}{r^*} \lesssim {\tau _{+}^{2s-1}} \end{aligned}$$

Combining (2.24) with (3.17) and the relation $2\,s + 2\delta < 1+\gamma$ gives

3.19a $$\begin{aligned} \left| ({\mathcal {L}}_{\widetilde{K^s}{}}g)_{{{\widetilde{L}}}{{\widetilde{L}}}}\right|&\lesssim \varepsilon _g {\tau _{+}^{-1-\delta }}{\tau _{-}^{\gamma }} \end{aligned}$$

3.19b $$\begin{aligned} \left| ({\mathcal {L}}_{\widetilde{K^s}{}}g)_{{\underline{{\widetilde{L}}}}{\underline{{\widetilde{L}}}}}\right|&\lesssim \varepsilon _g{\tau _{+}^{2s-2+\delta }}, \end{aligned}$$

3.19c $$\begin{aligned} \left| ({\mathcal {L}}_{\widetilde{K^s}{}}g)_{{{\widetilde{L}}}{\underline{{\widetilde{L}}}}} + 2\left( 2s({\underline{u}^{*{2s-1}}} + \text {sgn}({u^{*{}}}){|u^{*}|^{2s-1}})\right) \right|&\lesssim \varepsilon _g{\tau _{+}^{2s-2+\delta }}, \end{aligned}$$

3.19d $$\begin{aligned} \left| ({\mathcal {L}}_{\widetilde{K^s}{}}g)_{{{\widetilde{L}}}{{\widetilde{S}}_{j}}} \right|&\lesssim \varepsilon _g{\tau _{+}^{-1-\delta }}{\tau _{-}^{\gamma }} \end{aligned}$$

3.19e $$\begin{aligned} \left| ({\mathcal {L}}_{\widetilde{K^s}{}}g)_{{\underline{{\widetilde{L}}}}{{\widetilde{S}}_{j}}} \right|&\lesssim \varepsilon _g{\tau _{+}^{2s-2+\delta }}, \end{aligned}$$

3.19f $$\begin{aligned} \left| ({\mathcal {L}}_{\widetilde{K^s}{}}g)_{{{\widetilde{S}}_{i}}{{\widetilde{S}}_{j}}} - \delta _{ij}\tfrac{{\underline{u}^{*{2s}}} - {|u^{*}|^{2s}}}{r^*}\right|&\lesssim \varepsilon _g{\tau _{+}^{2s-2+\delta }}, \end{aligned}$$

The right-hand side vanishes if we replace *g* with ${{\widehat{m}}}$.We now look at terms which will appear in the energy momentum tensor. First, recalling the definition (3.4), a null decomposition on ${\textbf{G}}$ combined with (2.23) and (2.24) gives

3.20 $$\begin{aligned}{} & {} \left| {\textbf{G}}_{\gamma \delta }{\textbf{G}}^{\gamma \delta } - 2|\sigma |^2 + 2|\rho |^2 + 2\alpha \cdot {\underline{\alpha }}\right| \nonumber \\{} & {} \quad \lesssim \varepsilon _g({\tau _{+}^{-1+\delta }}|{\textbf{G}}_N|^2 + {\tau _{-}^{\gamma }}{\tau _{+}^{-1-\gamma +\delta }}|{\textbf{G}}|^2). \end{aligned}$$

Similar reasoning gives

3.21a $$\begin{aligned} \left| T_{{{\widetilde{L}}}{{\widetilde{L}}}}[{\textbf{G}}] - |{\alpha }|^2\right|&\lesssim \varepsilon _g({\tau _{+}^{-1+\delta }}|{\alpha }|^2 + {\tau _{0}^{\gamma }}{\tau _{+}^{-1+\delta }}|{\textbf{G}}_N|^2 + {\tau _{0}^{2\gamma }}{\tau _{+}^{-2+2\delta }}|{\textbf{G}}|^2), \end{aligned}$$

3.21b $$\begin{aligned} \left| T_{{{\widetilde{L}}}{\underline{{\widetilde{L}}}}}[{\textbf{G}}] - (|\sigma |^2 + |\rho |^2)\right|&\lesssim \varepsilon _g({\tau _{+}^{-1+\delta }}|{\textbf{G}}_N|^2 + {\tau _{0}^{\gamma }}{\tau _{+}^{-1+\delta }}|{\textbf{G}}|^2), \end{aligned}$$

3.21c $$\begin{aligned} \left| T_{{\underline{{\widetilde{L}}}}{\underline{{\widetilde{L}}}}}[{\textbf{G}}] - |{\underline{\alpha }}|^2\right|&\lesssim \varepsilon _g{\tau _{+}^{-1+\delta }}|{\textbf{G}}|^2, \end{aligned}$$

3.21d $$\begin{aligned} \left| T_{{{\widetilde{S}}_{1}}{{\widetilde{S}}_{1}}} + T_{{{\widetilde{S}}_{2}}{{\widetilde{S}}_{2}}}- (|\sigma |^2 + |\rho |^2)\right|&\lesssim \varepsilon _g({\tau _{+}^{-1+\delta }}|{\textbf{G}}_N|^2 + {\tau _{0}^{\gamma }}{\tau _{+}^{-1+\delta }}|{\textbf{G}}|^2), \end{aligned}$$

3.21e $$\begin{aligned} \left| T_{{{\widetilde{S}}_{j}}{{\widetilde{S}}_{j}}}\right|&\lesssim |{\textbf{G}}_N|^2 + \varepsilon _g{\tau _{0}^{\gamma }}{\tau _{+}^{-1+\delta }}|{\textbf{G}}|^2, \end{aligned}$$

3.21f $$\begin{aligned} \left| T_{{{\widetilde{S}}_{1}}{{\widetilde{S}}_{2}}}\right|&\lesssim |{\textbf{G}}_N|^2 + \varepsilon _g{\tau _{0}^{\gamma }}{\tau _{+}^{-1+\delta }}|{\textbf{G}}|^2, \end{aligned}$$

3.21g $$\begin{aligned} \left| T_{{{\widetilde{L}}}{{\widetilde{S}}_{j}}}\right|&\lesssim |\alpha ||{\overline{{\textbf{G}}}}| + \varepsilon _g({\tau _{0}^{\gamma }}{\tau _{+}^{-1+\delta }}|{\textbf{G}}||{\overline{{\textbf{G}}}}| + {\tau _{0}^{2\gamma }}{\tau _{+}^{-2+2\delta }}|{\textbf{G}}|^2), \end{aligned}$$

3.21h $$\begin{aligned} \left| T_{{\underline{{\widetilde{L}}}}{{\widetilde{S}}_{j}}}\right|&\lesssim |{\textbf{G}}||{\overline{{\textbf{G}}}}| + \varepsilon _g({\tau _{0}^{\gamma }}{\tau _{+}^{-1+\delta }}|{\textbf{G}}|^2). \end{aligned}$$

We now look at terms containing

$$\begin{aligned} \nabla ^\alpha \widetilde{K^s}{}^\beta T_{\alpha \beta }. \end{aligned}$$

We will symmetrize it and apply the identity (2.29).

#### Lemma 3.3

For a symmetric (0,2)-tensor *T* and a metric *g* satisfying (2.24),

3.22 $$\begin{aligned} \nabla ^\alpha \widetilde{K^s}{}^\beta T_{\alpha \beta }= & {} \tfrac{1}{2}\left( \tfrac{{\underline{u}^{*{2s}}} - {|u^{*}|^{2s}}}{r^*} - {\partial _{r^*}}\left( {\underline{u}^{*{2s}}} - {|u^{*}|^{2s}}\right) \right) T_{{{\widetilde{L}}}{\underline{{\widetilde{L}}}}} \nonumber \\{} & {} + \tfrac{1}{2}\tfrac{{\underline{u}^{*{2s}}} - {|u^{*}|^{2s}}}{r^*}\textrm{tr }(T) + R_1[T], \end{aligned}$$

where $R_1[T]$ is a remainder quantity satisfying

3.23 $$\begin{aligned} \left| R_1[T]\right| \lesssim \varepsilon _g\left( \tfrac{{\tau _{-}^{}}}{{\tau _{+}^{1+2\delta }}}|T| + {\tau _{+}^{2s-2+2\delta }}|T|_{{{\mathcal {T}}}{{\mathcal {U}}}}\right) . \end{aligned}$$

Additionally,

3.24 $$\begin{aligned} \tfrac{1}{2}\left( \tfrac{{\underline{u}^{*{2s}}} - {|u^{*}|^{2s}}}{r^*} - {\partial _{r^*}}\left( {\underline{u}^{*{2s}}} - {|u^{*}|^{2s}}\right) \right) \ge 0. \end{aligned}$$

If *T* is the energy-momentum tensor for some 2-form ${\textbf{G}}$, then

3.25 $$\begin{aligned} \left| R_1[T]\right| \lesssim \frac{\varepsilon _g}{{\tau _{+}^{1+2\delta }}}\left( {\tau _{+}^{2s}}(|{\alpha }[{\textbf{G}}]|^2 + |\rho [{\textbf{G}}]|^2 + |\sigma [{\textbf{G}}]|^2) + {\tau _{-}^{2s}}|{\underline{\alpha }}[{\textbf{G}}]|^2\right) . \end{aligned}$$

#### Proof

Symmetrizing and applying the identity (2.29) gives

3.26 $$\begin{aligned} \nabla ^\alpha \widetilde{K^s}{}^\beta T_{\alpha \beta } = \frac{1}{2}({{\widehat{m}}}+ (g-{{\widehat{m}}}))^{\alpha \gamma }({{\widehat{m}}}+ (g-{{\widehat{m}}}))^{\beta \delta }({\mathcal {L}}_{\widetilde{K^s}{}}g)_{\alpha \beta }T_{\gamma \delta }. \end{aligned}$$

We first examine the terms containing ${{\widehat{m}}}^{\alpha \gamma }{{\widehat{m}}}^{\beta \delta }$, for which we write

3.27 $$\begin{aligned} \frac{1}{2}{{\widehat{m}}}^{\alpha \gamma }{{\widehat{m}}}^{\beta \delta }({\mathcal {L}}_{\widetilde{K^s}{}}g)_{\alpha \beta }T_{\gamma \delta }= & {} \frac{1}{2}{{\widehat{m}}}^{\alpha \gamma }{{\widehat{m}}}^{\beta \delta }({\mathcal {L}}_{\widetilde{K^s}{}}{{\widehat{m}}})_{\alpha \beta }T_{\gamma \delta }\nonumber \\{} & {} + \frac{1}{2}{{\widehat{m}}}^{\alpha \gamma }{{\widehat{m}}}^{\beta \delta }({\mathcal {L}}_{\widetilde{K^s}{}}(g-{{\widehat{m}}}))_{\alpha \beta }T_{\gamma \delta }. \end{aligned}$$

We take the null decomposition and apply the estimates (3.19) (recalling that the right-hand side vanishes for $g = {{\widehat{m}}}$) to get

3.28 $$\begin{aligned} \frac{1}{2}{{\widehat{m}}}^{\alpha \gamma }{{\widehat{m}}}^{\beta \delta }({\mathcal {L}}_{\widetilde{K^s}{}}{{\widehat{m}}})_{\alpha \beta }T_{\gamma \delta }= & {} -\frac{1}{4} {\partial _{r^*}}\left( {\underline{u}^{*{2s}}} - {|u^{*}|^{2s}}\right) (T_{{{\widetilde{L}}}{\underline{{\widetilde{L}}}}} + T_{{\underline{{\widetilde{L}}}}{{\widetilde{L}}}})\nonumber \\{} & {} + \frac{1}{2}\sum _{i}\tfrac{{\underline{u}^{*{2s}}} - {|u^{*}|^{2s}}}{r^*}T_{{{\widetilde{S}}_{i}}{{\widetilde{S}}_{i}}}. \end{aligned}$$

This gives the first two terms on the left-hand side of (3.22) up to the error term

$$\begin{aligned} \tfrac{1}{2}\tfrac{{\underline{u}^{*{2s}}} - {|u^{*}|^{2s}}}{r^*}(g - {{\widehat{m}}})^{\alpha \beta }T_{\alpha \beta }. \end{aligned}$$

Expanding in the null frame and applying (2.28) allows us to bound this within $R_1$. Similarly, expanding and applying (3.19) gives the bound

3.29 $$\begin{aligned} \frac{1}{2}{{\widehat{m}}}^{\alpha \gamma }{{\widehat{m}}}^{\beta \delta }({\mathcal {L}}_{\widetilde{K^s}{}}(g-{{\widehat{m}}}))_{\alpha \beta }T_{\gamma \delta } \lesssim \varepsilon _g\Big (\tfrac{{\tau _{-}^{}}}{{\tau _{+}^{1+2\delta }}}|T| + {\tau _{+}^{2s-2+\delta }}|T|_{{{\mathcal {T}}}{{\mathcal {U}}}}\Big ). \end{aligned}$$

We now look at terms in (3.26) containing at least one factor of $g-{{\widehat{m}}}$. We may reduce this to bounding

$$\begin{aligned} \nabla ^\alpha \widetilde{K^s}{}^\beta T_{\alpha \beta } = \frac{1}{2} (g-{{\widehat{m}}})^{\alpha \gamma }{{\widehat{m}}}^{\beta \delta }({\mathcal {L}}_{\widetilde{K^s}{}}{{\widehat{m}}})_{\alpha \beta }T_{\gamma \delta }, \end{aligned}$$

which follows from symmetry of *T* and the fact that all terms which are quadratic in $g-{{\widehat{m}}}$ (including ${\mathcal {L}}_{\widetilde{K^s}{}}(g-{{\widehat{m}}})$) must decay like ${\tau _{+}^{2s-3+2\delta }}$ or better, which follows from (3.19) and (2.28). We take the null decomposition. We have only one nonzero term containing $T_{{\underline{{\widetilde{L}}}}{\underline{{\widetilde{L}}}}}$, which we can bound by

$$\begin{aligned} |(g-{{\widehat{m}}})^{{\underline{{\widetilde{L}}}}{\underline{{\widetilde{L}}}}}{{\widehat{m}}}^{{{\widetilde{L}}}{\underline{{\widetilde{L}}}}}({\mathcal {L}}_{\widetilde{K^s}{}}{{\widehat{m}}})_{{\underline{{\widetilde{L}}}}{{\widetilde{L}}}}T_{{\underline{{\widetilde{L}}}}{\underline{{\widetilde{L}}}}}|\lesssim \varepsilon _g {\tau _{-}^{\gamma }}{\tau _{+}^{2s-2-\gamma +\delta }}|T| \le \varepsilon _g {\tau _{-}^{}}{\tau _{+}^{-1-\delta }}. \end{aligned}$$

All other components may be bounded pointwise by $\varepsilon _g{\tau _{+}^{2s-2+\delta }}|T|_{{{\mathcal {T}}}{{\mathcal {U}}}}$.

In order to prove (3.24), we fix *t* and write $U(r^*) = {\underline{u}^{*{2\,s}}} - {|u^{*}|^{2\,s}}$. Then, $U(0) = 0$, and $U'' < 0$. By the mean value theorem, $\frac{U(r^*)}{r^*} = U'(r^\star )$ for some $r^\star \in (0, r^*)$. Since $U'$ is decreasing, $\frac{U(r^*)}{r^*} - U'(r^*)$ is always positive.

The estimate (3.25) follows almost directly from (3.23) and (3.21), noting

$$\begin{aligned} {\tau _{+}^{2s-2+\delta }}|T|_{{{\mathcal {T}}}{{\mathcal {U}}}}\lesssim & {} {\tau _{+}^{2s-2+\delta }}|{\textbf{G}}||{\overline{{\textbf{G}}}}| + {\tau _{+}^{2s-3+2\delta }}|{\textbf{G}}|^2\\\lesssim & {} {\tau _{+}^{2s-1-\delta }}|{\overline{{\textbf{G}}}}|^2 + {\tau _{+}^{2s-3+3\delta }}|{\textbf{G}}|^2. \end{aligned}$$

$$\square $$

Now we look at the terms where the derivative falls on the weight. We decompose

3.30 $$\begin{aligned} \nabla ^\alpha (w)= & {} g^{\alpha {\underline{{\widetilde{L}}}}}{\underline{{\widetilde{L}}}}({\widetilde{w}}) + g^{\alpha {{\widetilde{L}}}}{{\widetilde{L}}}({\widetilde{w}}) = -\tfrac{1}{2}{\underline{{\widetilde{L}}}}({\widetilde{w}}){{\widetilde{L}}}^\alpha - \tfrac{1}{2}{{\widetilde{L}}}({\widetilde{w}}){\underline{{\widetilde{L}}}}^\alpha \nonumber \\{} & {} + (g-{{\widehat{m}}})^{\alpha {\underline{{\widetilde{L}}}}}{\underline{{\widetilde{L}}}}({\widetilde{w}})+(g-{{\widehat{m}}})^{\alpha {{\widetilde{L}}}}{{\widetilde{L}}}({\widetilde{w}}). \end{aligned}$$

(3.21), along with the inequalities $1+2{{\overline{\mu }}} < \min (2\,s, 2\gamma )$, $1+\gamma -2\,s \ge 4\delta$, implies

3.31a $$\begin{aligned} |T_{\widetilde{K^s}{}{{\widetilde{L}}}} - ({\tau _{+}^{2s}}|\alpha |^2 + {\tau _{-}^{2s}}(|\sigma |^2 + |\rho |^2)|&\lesssim \varepsilon _g({\tau _{+}^{2s-1+\delta }}|\alpha |^2 \nonumber \\&\quad + {\tau _{-}^{\gamma }}{\tau _{+}^{2s-1-\gamma +\delta }}|{\textbf{G}}_N|^2 + {\tau _{0}^{1+2{\overline{\mu }}}}{\tau _{-}^{\gamma }}|{\textbf{G}}|^2) \end{aligned}$$

3.31b $$\begin{aligned} |T_{\widetilde{K^s}{}{\underline{{\widetilde{L}}}}} - ({\tau _{+}^{2s}}(|\sigma |^2 + |\rho |^2)+ {\tau _{-}^{2s}}|{\underline{\alpha }}|^2)|&\lesssim \varepsilon _g({\tau _{+}^{2s-1+\delta }}|{\textbf{G}}_N|^2 + {\tau _{-}^{2s}}|{\textbf{G}}|^2). \end{aligned}$$

The inequality

$$\begin{aligned} {\tau _{0}^{\gamma }}{\tau _{+}^{2s-1+\delta }}|{\textbf{G}}_N|^2 \lesssim {\tau _{+}^{2s}}{\tau _{0}^{1+2\mu }}|{\overline{{\textbf{G}}}}|^2 + {\tau _{+}^{2s}}|\alpha |^2 + {\tau _{+}^{2s-2+2\delta }}{\tau _{0}^{2\gamma }}|{\textbf{G}}|^2 \end{aligned}$$

implies

3.32 $$\begin{aligned} -\tfrac{1}{2}{\underline{{\widetilde{L}}}}({\widetilde{w}})T_{\widetilde{K^s}{}{{\widetilde{L}}}} - \tfrac{1}{2}{{\widetilde{L}}}({\widetilde{w}})T_{\widetilde{K^s}{}{\underline{{\widetilde{L}}}}} > rsim (1-C\varepsilon _g)S_0[{\textbf{G}}]. \end{aligned}$$

Therefore, for sufficiently small $\varepsilon _1$, we obtain the requisite spacetime integral. To bound other terms, we start with the estimates

3.33a $$\begin{aligned} |T_{\widetilde{K^s}{}\alpha }(g-{{\widehat{m}}})^{\alpha {\underline{{\widetilde{L}}}}}{\underline{{\widetilde{L}}}}({\widetilde{w}})|&\lesssim \varepsilon _g{\tau _{+}^{-1+\delta }}(|T_{\widetilde{K^s}{}{{\widetilde{L}}}}|+{\tau _{0}^{\gamma }}|T_{\widetilde{K^s}{}{\underline{{\widetilde{L}}}}}|+{\tau _{0}^{\gamma }}|T_{\widetilde{K^s}{}{{\widetilde{S}}_{1}}}|+{\tau _{0}^{\gamma }}|T_{\widetilde{K^s}{}{{\widetilde{S}}_{2}}}|)w' \end{aligned}$$

3.33b $$\begin{aligned} |T_{\widetilde{K^s}{}\alpha }(g-{{\widehat{m}}})^{\alpha {{\widetilde{L}}}}{{\widetilde{L}}}({\widetilde{w}})|&\lesssim \varepsilon _g{\tau _{+}^{-1+\delta }}{\tau _{0}^{1+2{{\overline{\mu }}}}}(|T_{\widetilde{K^s}{}{{\widetilde{L}}}}|+|T_{\widetilde{K^s}{}{\underline{{\widetilde{L}}}}}|+|T_{\widetilde{K^s}{}{{\widetilde{S}}_{1}}}|+|T_{\widetilde{K^s}{}{{\widetilde{S}}_{2}}}|)w' \end{aligned}$$

These may be straightforwardly bounded using (3.21). First,

$$\begin{aligned} \int _0^T{\int _{\Sigma _{t}}}|\varepsilon _g{\tau _{+}^{-1+\delta }}T_{\widetilde{K^s}{}{{\widetilde{L}}}}w'|\lesssim \varepsilon _gS_0. \end{aligned}$$

We handle metric error terms on the right-hand side of (3.21) (those containing $\varepsilon _g$) by first bounding the correlated terms in (3.33) (pointwise) by $\varepsilon _g{\tau _{0}^{\gamma }}{\tau _{+}^{2s-2+2\delta }}|{\textbf{G}}|^2w'$. Using $2s-2-\gamma +2\delta < -1-2{\overline{\mu }}$, these are bounded by $S_0[{\textbf{G}}]$. The remaining integral is bounded by

$$\begin{aligned} C\int _0^T{\int _{\Sigma _{t}}}|\varepsilon _g{\tau _{+}^{-1+\delta }}{\tau _{0}^{\gamma }}({\tau _{+}^{2s}}(|\alpha |^2+|\rho |^2+|\sigma |^2) + {\tau _{-}^{2s}}|{\underline{\alpha }}|^2)w'|. \end{aligned}$$

The assumptions on $\gamma , \delta , {\overline{\mu }}$ in Theorem 1.1 imply ${\tau _{0}^{\gamma }}{\tau _{+}^{-1+\delta }}\le {\tau _{0}^{1+2{{\overline{\mu }}}}}$, so consequently

3.34 $$\begin{aligned} \int _0^T{\int _{\Sigma _{t}}}T[{\textbf{G}}]_{\alpha \beta }\widetilde{K^s}{}^\beta \nabla ^\alpha ({\widetilde{w}})\, dx\, dt \ge (1-C\varepsilon _g)S_0[{\textbf{G}}] \end{aligned}$$

We now bound the boundary terms. Since $|g|\approx 1$, it suffices to show that

3.35 $$\begin{aligned} {\int _{\Sigma _{t}}}P[{\textbf{G}}]^0 \approx E_0[{\textbf{G}}](t). \end{aligned}$$

In order to bound this, we apply (2.23) and (2.24), and (3.21).

The bounds for the light cone follow from divergence theorem on regions $t\in (0,T)$, ${u^{*{}}}\le U$. It suffices to bound

3.36 $$\begin{aligned} \int _{C_{U, T}}-\nabla ^\alpha ({u^{*{}}})\sqrt{|g|}P[{\textbf{G}}]_\alpha \, dV_{{\overline{g}}}, \end{aligned}$$

where $V_{{\overline{g}}}$ is the volume with respect to the induced metric, which is equivalent to the background Minkowski metric. By (2.23) and (2.24),

3.37 $$\begin{aligned} \left| -\nabla ^\alpha ({u^{*{}}})P[{\textbf{G}}]_\alpha - \frac{1}{2} P[{\textbf{G}}]_{{\widetilde{L}}}\right|\lesssim & {} \varepsilon _g{\tau _{+}^{-1+\delta }}|P[{\textbf{G}}]|_{{\widetilde{L}}}\nonumber \\{} & {} +\varepsilon _g {\tau _{0}^{\gamma }}{\tau _{+}^{-1+\delta }}\left( |P[{\textbf{G}}]_{{\widetilde{S}}_{1}}| +|P[{\textbf{G}}]_{{\widetilde{S}}_{2}}| + |P[{\textbf{G}}]_{\underline{{\widetilde{L}}}}|\right) .\nonumber \\ \end{aligned}$$

(3.21) gives the component estimate

3.38 $$\begin{aligned} \left| -\nabla ^\alpha ({u^{*{}}})P[{\textbf{G}}]_\alpha - \frac{1}{2} P[{\textbf{G}}]_{{\widetilde{L}}}\right|\lesssim & {} \varepsilon _g\left( {\tau _{+}^{2s-1+\delta }}|\alpha |^2 + {\tau _{-}^{2s}}{\tau _{+}^{-1+\delta }}|{\overline{{\textbf{G}}}}|^2 \right. \nonumber \\{} & {} \left. + {\tau _{-}^{2s+\gamma }}{\tau _{+}^{-1-\gamma +\delta }}|{\textbf{G}}|^2\right) {\widetilde{w}}. \end{aligned}$$

This is unfortunately not positive definite in itself. We define

3.39 $$\begin{aligned} C^+_0[{\textbf{G}}](T) = \sup _{{u^{*{}}}}\int _{C_{U, T}}\left( {\tau _{+}^{2s}}|\alpha |^2+ {\tau _{-}^{2s}}(|\sigma |^2+|\rho |^2)\right) w \, dV_C, \end{aligned}$$

In the course of our $L^\infty$ estimates, we will show

3.40 $$\begin{aligned} |C^+_0[{\textbf{G}}](T)|\lesssim |C_0[{\textbf{G}}](T)| + \varepsilon _g|E_2[{\textbf{G}}](T)|. \end{aligned}$$

by integrating certain $L^\infty$ estimates which depend only on our time-slice and interior energies. The result (except for the conical energy terms) follows from an application of the divergence theorem (in Minkowski space) on the quantity $\sqrt{|g|}P[{\textbf{G}}]^\alpha$, over the time slab $[0,T] \times {\mathbb {R}}^3$. First,

$$\begin{aligned} {\int _{\Sigma _{T}}}\sqrt{|g|}P[F]^0 - {\int _{\Sigma _{0}}}\sqrt{|g|}P[F]^0= & {} \int _0^T {\int _{\Sigma _{t}}}\sqrt{|g|} \left( {\textbf{G}}(\widetilde{K^s}{}, J) {\widetilde{w}}\right. \nonumber \\{} & {} \left. - T[{\textbf{G}}]_{\alpha \beta }\widetilde{K^s}{}^{\beta ;\alpha }{\widetilde{w}} - T[{\textbf{G}}]_{\alpha \beta }\widetilde{K^s}{}^\beta \nabla ^\alpha ({\widetilde{w}})\right) \, dx \, dt. \end{aligned}$$

Applying (3.35) on the left, Lemma 3.3 and (3.34) on the right, and moving the resulting terms to the left, gives

3.41 $$\begin{aligned} E_0[{\textbf{G}}](T) + S_0[{\textbf{G}}](T)\lesssim & {} E_0[{\textbf{G}}](0) + \int _0^T {\int _{\Sigma _{t}}}\left| {\textbf{G}}(\widetilde{K^s}{}, {\textbf{J}})\right| w\, dx\, dt\nonumber \\{} & {} + \int _0^T \tfrac{E_0(t)}{(1+t)^{1+2\delta }} \, dt. \end{aligned}$$

The last term on the right is bounded by $C\varepsilon _1E_0[{\textbf{G}}](T)$. To bound the current term, we take the null decomposition and obtain the bounds

3.42a $$\begin{aligned} |{\textbf{J}}^{{\underline{{\widetilde{L}}}}}{\textbf{G}}_{\widetilde{K^s}{}{\underline{{\widetilde{L}}}}}|&\lesssim {\tau _{+}^{2s}}|{\textbf{J}}^{{\underline{{\widetilde{L}}}}}||\rho | \end{aligned}$$

3.42b $$\begin{aligned} |{\textbf{J}}^{{\widetilde{S}}_{j}}{\textbf{G}}_{\widetilde{K^s}{}{{\widetilde{S}}_{j}}}|&\lesssim |{\textbf{J}}^{{{\widetilde{S}}_{j}}}|\left( {\tau _{+}^{2s}}|\alpha | + {\tau _{-}^{2s}}|{\underline{\alpha }}|\right) \end{aligned}$$

3.42c $$\begin{aligned} |{\textbf{J}}^{{{\widetilde{L}}}}{\textbf{G}}_{\widetilde{K^s}{}{{\widetilde{L}}}}|&\lesssim {\tau _{-}^{2s}}|{\textbf{J}}^{{{\widetilde{L}}}}||{\underline{\alpha }}| \end{aligned}$$

Applying Hölder’s inequality with the current norm (3.6) gives

3.43 $$\begin{aligned} \int _0^T {\int _{\Sigma _{t}}}|{\textbf{G}}(\widetilde{K^s}{}, {\textbf{J}})|w \, dx \, dt\lesssim & {} S_0[{\textbf{G}}](T)^{1/2}\left\Vert {\textbf{J}}\right\Vert _{L^2[w]} \nonumber \\\lesssim & {} C^{-1}S_0[{\textbf{G}}](T) + C\left\Vert {\textbf{J}}\right\Vert ^2_{L^2[w]}. \end{aligned}$$

We apply this to (3.41). Then, for sufficiently large *C* independent of $\varepsilon , \varepsilon _g$, we may rewrite (3.41) as

3.44 $$\begin{aligned} E_0[{\textbf{G}}](T) + S_0[{\textbf{G}}](T)\le & {} C'E_0[{\textbf{G}}](0) + \frac{1}{2}(E_0[{\textbf{G}}](T)\nonumber \\{} & {} + S_0[{\textbf{G}}](T)) + C'\left\Vert {\textbf{J}}\right\Vert ^2_{L^2[w]} \end{aligned}$$

for some $C'$, and subtract off the $E_0[{\textbf{G}}](T)$ and $S_0[{\textbf{G}}](T)$ terms on the right-hand side while retaining a coercive energy on the left. Finally, in order to include the conical energy, we repeat the divergence theorem on regions of the form

$$\begin{aligned} ([0,T] \times {\mathbb {R}}^3) \cap \{{u^{*{}}}\le U\} \end{aligned}$$

and take the maximum integral over the reduced light cones. $\square$

## $L^2$ Estimates for $\varvec{\phi }$

We now establish a conformal energy estimate for a generic function $\phi$. Our approach is a modification of the one used in [3] and builds on the results of Sect. 2.6. We recall the acceleration field

$$\begin{aligned} K_0 = \frac{1}{2}(\underline{u}^2(\partial _t+\partial _r) + u^2(\partial _t - \partial _r)). \end{aligned}$$

Repeating the argument of Theorem 3.1, would give an error term of $t(\text {tr }Q[\varvec{\phi }])$ (which vanishes for $Q[{\textbf{G}}]$). We instead reframe the argument of Sect. 2.6, which uses the fact that $K_0$ is a conformal Killing field.

Away from the spatial origin the identities (1.2), (1.10) imply

4.1 $$\begin{aligned} \Box _g^{{\mathbb {C}}}\phi = \Box _g^{{\mathbb {C}}}\tfrac{r^*\phi }{r^*} = \tfrac{1}{r^*}\Box _g^{{\mathbb {C}}}(r^*\phi ) + 2 \nabla ^\alpha (\tfrac{1}{r^*})D_\alpha (r^*\phi ) + (r^*\phi )\Box _g(\tfrac{1}{r^*}) \end{aligned}$$

The singular behavior at $r^* = 0$ is of concern, as $\frac{1}{r^*}$ is not a solution of the geometric wave equation. We instead establish an estimate with a background metric ${{\widehat{m}}}$, as defined in 2.19, and bound the remainder as error terms.

We first state the energies we will use, which are analogous to those in Theorem 3.1. We define the standard energy 4.2aIt follows from Lemma 10.7 that this is equivalent to4.2b

Likewise, we have the spacetime energy4.3and the weak conical energy

4.4 $$\begin{aligned} C_0[\phi ](T) = \sup _{U}\int _{C_{U,T}} T(\nabla {u^{*{}}}, \widetilde{K^s}{}) w \, dV_C, \end{aligned}$$

where the partial energy momentum tensor *T* is defined in equation (4.13). We take the opportunity to define $U^\star$ to be the value of *U* for which $C_0$ is defined, and $C^U_{ext}$ to be the set

$$\begin{aligned} C^U_{ext}= \bigcup _{{u^{*{}}}\le U}C_{{u^{*{}}},T} \end{aligned}$$

We then define the strong conical energy

$$\begin{aligned} C_0^+[\phi ](U,T) = \int _{C_{U,T}}\left( \left| {\tau _{+}^{2s}}\tfrac{D_{{{\widetilde{L}}}}(r^*\phi )}{r^*}\right| ^2 + {\tau _{-}^{2s}}\sum _i\left| D_{{{\widetilde{S}}_{i}}}\phi \right| ^2 + {\tau _{+}^{2s}}{\tau _{0}^{2}}\left| \tfrac{\phi }{r^*}\right| ^2\right) w \, dV_C. \end{aligned}$$

We will show later

$$\begin{aligned} |C_0^+[\phi ](U,T)| \lesssim |C_0[\phi ](T)| + \varepsilon _g |E_2[\phi ](T)|. \end{aligned}$$

We also define the combined energies

4.5 $$\begin{aligned} {\mathcal {E}}_0[\phi ](T) = E_0[\phi ](T) + S_0[\phi ](T) + C_0[\phi ](T) \end{aligned}$$

and

4.6 $$\begin{aligned} {\mathcal {E}}_k[\phi ](T) = \sum _{|I| \le k} {{\mathcal {E}}}_0[D_{{\widetilde{X}}}^I\phi ](T). \end{aligned}$$

If $({\textbf{F}}, \varvec{\phi })$ solves (1.14), we define the full energy

4.7 $$\begin{aligned} {\mathcal {E}}_k(T) = {\mathcal {E}}_k[\varvec{\phi }](T) + {\mathcal {E}}_k[{\textbf{F}}^1](T) + |q[{\textbf{F}}]|^2. \end{aligned}$$

When there is no ambiguity, we write ${\mathcal {E}}_k$. We define the analogous quantities $E_k[\phi ](T)$, $S_k[\phi ](T)$, $C_k[\phi ](T)$ similarly. We can now state the main theorem of this section.

### Theorem 4.1

For a sufficiently regular function $\phi$ with sufficient spatial decay on $[0,T]\times {\mathbb {R}}^3$, we have

4.8 $$\begin{aligned} {\mathcal {E}}_0[\phi ](T) \lesssim {\mathcal {E}}_0[\phi ](0) + \left( |q|+\left\Vert {{\textbf{F}}^1} \right\Vert ^{red}_{L^{\infty }[w]}\right) {\mathcal {E}}_0[\phi ](T) + \left\Vert {\tau _{+}^{s}}{\tau _{-}^{1/2}}(\Box _g^{{\mathbb {C}}}\phi ) w_{\delta }^{1/2}\right\Vert _2^2,\nonumber \\ \end{aligned}$$

where

4.9 $$\begin{aligned} \left\Vert {{\textbf{F}}^1} \right\Vert ^{red}_{L^{\infty }[w]}= & {} \sup _{[0,T]\times {\mathbb {R}}^3}{\tau _{+}^{}}{\tau _{-}^{1/2+s}}{\underline{\alpha }}[{{\textbf{F}}^1}]w^{1/2} \nonumber \\{} & {} + {\tau _{+}^{1+s}}{\tau _{-}^{1/2}}(|\rho [{{\textbf{F}}^1}]| + |\sigma [{{\textbf{F}}^1}]|)w^{1/2} + {\tau _{+}^{3/2+s}}|{\alpha }[{{\textbf{F}}^1}]|w^{1/2}.\nonumber \\ \end{aligned}$$

### Remark 4.2

It is here that we must exclude the endpoint $s=\frac{1}{2}$, as we can no longer rely on the conformal structure of the metric to bound (4.22).

We will use Lemma 4.5 to show this for ${{\widehat{m}}}$, then we handle the error terms using Lemma 4.6. We start with some preliminary estimates.

### Remark 4.3

For the majority of this section, we will raise and lower the metric with respect to ${{{\widehat{m}}}}$. To avoid ambiguity, we will use the notation

$$\begin{aligned} \nabla _{{{\widehat{m}}}}^\alpha = {{\widehat{m}}}^{\alpha \beta }\nabla _\alpha , \quad D_{{{\widehat{m}}}}^\alpha = {{\widehat{m}}}^{\alpha \beta }D_\alpha . \end{aligned}$$

We note the following inequalities, which will be used many times in the future.

### Proposition 4.4

Given the definitions (4.2a) and (4.3), the following estimates hold:

4.10a $$\begin{aligned} \left\Vert {\tau _{+}^{-1/2-\delta }}{\tau _{-}^{s}}|D\phi |w^{1/2} \right\Vert _2^2&\lesssim E_0[\phi ](T) \end{aligned}$$

4.10b $$\begin{aligned} \left\Vert {\tau _{+}^{s-3/2-\delta }}|\phi |w^{1/2} \right\Vert _2^2&\lesssim E_0[\phi ](T) \end{aligned}$$

4.10c $$\begin{aligned} \left\Vert {\tau _{+}^{s-1/2-\delta }}|{\overline{D}}\phi |w^{1/2} \right\Vert _2^2&\lesssim E_0[\phi ](T) \end{aligned}$$

4.10d $$\begin{aligned} \left\Vert {\tau _{+}^{2s-2+\delta }}|{\overline{D}}\phi ||D\phi |w\right\Vert _1&\lesssim E_0[\phi ](T) \end{aligned}$$

4.10e $$\begin{aligned} \left\Vert {\tau _{+}^{-s}} \frac{D_{\widetilde{K^s}{}}(r^*\phi )}{r^*}(w')^{1/2}\right\Vert _2^2&\lesssim S_0[\phi ](T). \end{aligned}$$

### Proof

Inequalities (4.10a)–(4.10d) follow from the inequality

4.11 $$\begin{aligned} \int _0^T \frac{E_0[\phi ](t)}{(1+t)^{1+2\delta }}\, dt \lesssim E_0[\phi ](T). \end{aligned}$$

Additionally, (4.10d) follows from an application of Hölder’s inequality, combined with the inequality $s-3/2+2\delta < -1/2-\delta$. Inequality (4.10e) comes from our spacetime energy norm and the inequality $\frac{1}{2} + {{\overline{\mu }}} < s$. $\square$

Then it follows from (2.24), (2.23), (4.10a) and (4.10d), as well as the inequality $2s-1-\gamma +4\delta < 0$, that

4.12 $$\begin{aligned} \left\Vert {\tau _{+}^{2s-1}}H_1^{\gamma \delta }D_\gamma \phi \overline{D_\delta \phi } w\right\Vert _1 \lesssim \varepsilon _g E_0[\phi ](T), \end{aligned}$$

which will be useful later. We define the modified energy momentum tensor

4.13 $$\begin{aligned} {\widetilde{T}}[\phi ]_{\alpha \beta } = \frac{1}{r^{*2}}{\mathfrak {R}}\left( D_\alpha (r^*\phi )\overline{D_\beta (r^*\phi )} - \frac{1}{2} {{\widehat{m}}}_{\alpha \beta }{{\widehat{m}}}^{\gamma \delta }D_\gamma (r^*\phi )\overline{D_\delta (r^*\phi )} \right) .\nonumber \\ \end{aligned}$$

It follows that

4.14 $$\begin{aligned} \nabla _{{\widehat{m}}}^\alpha {\widetilde{T}}[\phi ]_{\alpha \beta }= & {} -\tfrac{2\nabla _{{\widehat{m}}}^\alpha (r^*)}{r^{*}}{\widetilde{T}}[\phi ]_{\alpha \beta }\nonumber \\{} & {} + \tfrac{1}{r^{*2}}{\mathfrak {R}}\left( \Box _{{\widehat{m}}}^{{\mathbb {C}}}(r^*\phi )\overline{D_\beta (r^*\phi )}\right) \nonumber \\{} & {} + \tfrac{1}{r^{*2}}{\mathfrak {I}}\left( r^*\phi \overline{D_{{{\widehat{m}}}}^{\alpha }(r^*\phi )}\right) {\textbf{F}}_{\alpha \beta }. \end{aligned}$$

Identity (4.1) gives

$$\begin{aligned} \tfrac{1}{r^{*2}}{\mathfrak {R}}\left( \Box _{{\widehat{m}}}^{{\mathbb {C}}}(r^*\phi )\overline{D_\beta (r^*\phi )}\right) = {\mathfrak {R}}\left( \left( \Box _{{\widehat{m}}}^{{\mathbb {C}}}\phi - 2\nabla _{{\widehat{m}}}^\alpha \tfrac{1}{r^*}D_\alpha (r^*\phi )- r^{*}\phi \Box _{{\widehat{m}}}\left( \tfrac{1}{r^*}\right) \right) \tfrac{\overline{D_\beta (r^*\phi )}}{r^*}\right) . \end{aligned}$$

Combining these and taking the necessary cancellations gives us

4.15 $$\begin{aligned} \nabla _{{\widehat{m}}}^\alpha {\widetilde{T}}[\phi ]_{\alpha \beta }= & {} \tfrac{\nabla _{{\widehat{m}}}^\alpha r^*}{r^{*3}}{{\widehat{m}}}_{\alpha \beta }D_\gamma (r^*\phi )\overline{D_{{{\widehat{m}}}}^\gamma (r^*\phi )}\nonumber \\{} & {} +{\mathfrak {R}}\left( \left( \Box _{{\widehat{m}}}^{{\mathbb {C}}}\phi - r^{*}\phi \Box _{{\widehat{m}}}\left( \tfrac{1}{r^*}\right) \right) \overline{\tfrac{D_\beta (r^*\phi )}{r^*}}\right) + {\mathfrak {I}}\left( \phi \tfrac{\overline{D_{{{\widehat{m}}}}^{\alpha }(r^*\phi )}}{r^*}\right) {\textbf{F}}_{\alpha \beta }\nonumber \\ \end{aligned}$$

We now contract with $\widetilde{K^s}{}$. Since $\Box _{{\widehat{m}}}(1/r^*)$ vanishes away from 0 and $\widetilde{K^s}{}(r^*)$ vanishes at 0,

4.16 $$\begin{aligned} {\mathfrak {R}}\left( r^{*}\phi \Box _{{\widehat{m}}}\left( \tfrac{1}{r^*}\right) \overline{\tfrac{D_\beta (r^*\phi )}{r^*}} \widetilde{K^s}{}^\beta w\right) = 0. \end{aligned}$$

To bound the last term on the right of (4.15), we split ${\textbf{F}}= {\textbf{F}}^0 + {\textbf{F}}^1$. Then, $w \lesssim {\tau _{-}^{}}w'$ in the support of ${\textbf{F}}^0$ so

4.17 $$\begin{aligned} \left\Vert \phi \tfrac{\overline{D_{{{\widehat{m}}}}^\alpha (r^*\phi )}}{r^*}{\textbf{F}}^0_{\alpha \beta }\widetilde{K^s}{}^\beta w \right\Vert _1\lesssim & {} \left\Vert {\tau _{+}^{s-1}}{\tau _{0}^{1/2+{\overline{\mu }}}}\phi (w')^{1/2}\right\Vert _2\nonumber \\{} & {} \left\Vert {\tau _{+}^{2-s}}{\tau _{0}^{1/2-{\overline{\mu }}}}\tfrac{D_{{{\widehat{m}}}}^\alpha (r^*\phi )}{r^*}{\textbf{F}}^0_{\alpha \beta }\widetilde{K^s}{}^\beta (w')^{1/2}\right\Vert _2.\nonumber \\ \end{aligned}$$

The first term on the right is easily bounded by ${\mathcal {E}}[\phi ]^{1/2}$. Applying (2.50) and expanding in the null frame gives

4.18 $$\begin{aligned} \left| \tfrac{D_{{{\widehat{m}}}}^\alpha (r^*\phi )}{r^*}{\textbf{F}}^0_{\alpha \beta }\widetilde{K^s}{}^\beta \right| \lesssim |q|\left( \left| {\tau _{+}^{2s-2}}\tfrac{D_{{{\widetilde{L}}}}(r^*\phi )}{r^*}\right| + \left| {\tau _{-}^{2s}}{\tau _{+}^{-2}}\tfrac{D_{{\underline{{\widetilde{L}}}}}(r^*\phi )}{r^*}\right| \right) . \end{aligned}$$

The estimate $\tfrac{1}{2} + {\overline{\mu }} < s$ gives

4.19 $$\begin{aligned} \left\Vert \phi \tfrac{\overline{D_{{{\widehat{m}}}}^\alpha (r^*\phi )}}{r^*}{\textbf{F}}^0_{\alpha \beta }\widetilde{K^s}{}^\beta w \right\Vert _1 \lesssim |q|S_0[\phi ](T). \end{aligned}$$

To handle the ${\textbf{F}}^1$ terms, we first take

4.20 $$\begin{aligned} \left\Vert \phi \tfrac{\overline{D_{{{\widehat{m}}}}^\alpha (r^*\phi )}}{r^*}{\textbf{F}}^1_{\alpha \beta }\widetilde{K^s}{}^\beta w \right\Vert _1\lesssim & {} \left\Vert {\tau _{+}^{s-3/2-{\overline{\mu }}}}\phi w^{1/2}\right\Vert _2\nonumber \\{} & {} \left\Vert {\tau _{+}^{3/2+{\overline{\mu }}-s}}\tfrac{D_{{{\widehat{m}}}}^\alpha (r^*\phi )}{r^*}{\textbf{F}}^1_{\alpha \beta }\widetilde{K^s}{}^\beta w^{1/2}\right\Vert _2. \end{aligned}$$

Then,4.21Combining this with (4.20) and (4.19), as well as $\tfrac{1}{2} + {{\overline{\mu }}} < s$, gives

4.22 $$\begin{aligned} \big \Vert \phi \tfrac{\overline{D_{{{\widehat{m}}}}^\alpha (r^*\phi )}}{r^*}{\textbf{F}}_{\alpha \beta }\widetilde{K^s}{}^\beta w \big \Vert _1 \lesssim (|q| + \Vert {\textbf{F}}^1\Vert ^{red}_{L^{\infty }[w]})S_0[\phi ](T). \end{aligned}$$

We recall the deformation tensor estimate (3.28)

4.23 $$\begin{aligned} \text {symm }\nabla _{{\widehat{m}}}^\alpha \widetilde{K^s}{}^\beta= & {} \tfrac{1}{2} \tfrac{{\underline{u}^{*{}}}^{2s} - {|u^{*}|^{}}^{2s}}{r^*}{{\widehat{m}}}^{\alpha \beta } + \tfrac{1}{4}(\tfrac{{\underline{u}^{*{}}}^{2s} - {|u^{*}|^{}}^{2s}}{r^*}) \nonumber \\{} & {} - 2s({\underline{u}^{*{}}}^{2s-1} + {u^{*{}}}{|u^{*}|^{}}^{2s-2}))({{\widetilde{L}}}^\alpha {\underline{{\widetilde{L}}}}^\beta + {{\widetilde{L}}}^\beta {\underline{{\widetilde{L}}}}^\alpha ), \end{aligned}$$

This allows us to establish the estimate in Minkowski space:

### Lemma 4.5

Define ${\mathcal {E}}_0^+(U,T) = E_0[\phi ](T) + S_0[\phi ](T) + C_0^+[\phi ](U,T)$. Given a function $\phi$ of sufficient regularity and decay, we have the bound

4.24 $$\begin{aligned} {\mathcal {E}}_0^+[\phi ](T)&\lesssim {\mathcal {E}}_0[\phi ](0) + \left( |q| + \Vert {\textbf{F}}^1 \Vert ^{red}_{L^{\infty }[w]}\right) {\mathcal {E}}_0[\phi ](T) \nonumber \\&\quad + \left| \int _{[0,T]\times \Sigma _t}\Box _{{\widehat{m}}}^{\mathbb {C}}\phi \tfrac{\overline{D_{\widetilde{K^s}{}}(r^*\phi )}}{r^*}w\right| ++ \left| \int _{C^U_{ext}}\Box _{{{\widehat{m}}}}^{\mathbb {C}}\phi \tfrac{\overline{D_{\widetilde{K^s}{}}(r^*\phi )}}{r^*}w\right| \end{aligned}$$

### Proof

This follows from an application of the divergence theorem over the regions $[0,T]\times {\mathbb {R}}^3$ and $C^U_{ext}$.

We have that

4.25 $$\begin{aligned} -\nabla _{{\widehat{m}}}^\alpha \left( {\widetilde{T}}[\phi ]_{\alpha \beta }\widetilde{K^s}{}^\beta {\widetilde{w}}\right)= & {} - (\nabla _{{\widehat{m}}}^{\alpha }\widetilde{K^s}{}^\beta ) {\widetilde{T}}_{\alpha \beta }\widetilde{w} \nonumber \\{} & {} - \widetilde{K^s}{}^\beta \nabla _{{\widehat{m}}}^\alpha \widetilde{T}_{\alpha \beta }\widetilde{w} - (\nabla _{{\widehat{m}}}\widetilde{w})^\alpha \widetilde{K^s}{}^\beta \widetilde{T}_{\alpha \beta } \end{aligned}$$

The boundary terms on regions like $[0,T]\times {\mathbb {R}}^3$ give $E_0$, expressed as (4.2b), and the additional boundary term on the $C^U_{ext}$ integral gives $C_0$.

Symmetrizing the first term on the right-hand side of (4.25), applying (4.23) and (4.15) along with positivity of ${\widetilde{T}}_{{{\widetilde{L}}}{\underline{{\widetilde{L}}}}}$ and the identities

$$\begin{aligned} \tfrac{\nabla _{{\widehat{m}}}^\alpha r^*}{r^{*}}\widetilde{K^s}{}^\beta {{\widehat{m}}}_{\alpha \beta } = \tfrac{1}{2}\tfrac{{\underline{u}^{*{}}}^{2s} - {|u^{*}|^{}}^{2s}}{r^*}, \quad \text {tr}_{{{\widehat{m}}}}\widetilde{T} = -\tfrac{1}{r^{*2}}D_\alpha (r^*\phi )D^\alpha (r^*\phi ). \end{aligned}$$

gives

4.26 $$\begin{aligned} -\nabla _{{\widehat{m}}}^\alpha \left( {\widetilde{T}}[\phi ]_{\alpha \beta }\widetilde{K^s}{}^\beta {\widetilde{w}}\right)\le & {} -\tfrac{1}{r^{*2}}{\mathfrak {R}}\left( \Box _{{\widehat{m}}}^{{\mathbb {C}}}(r^*\phi )\overline{D_{\widetilde{K^s}{}}(r^*\phi )}\right) \nonumber \\{} & {} - \tfrac{1}{r^{*2}}{\mathfrak {I}}\left( r^*\phi \overline{D_{{{\widehat{m}}}}^{\alpha }(r^*\phi )}\right) {\textbf{F}}_{\alpha \beta }\widetilde{K^s}{}^\beta \nonumber \\{} & {} - \nabla ^\alpha _{{\widehat{m}}}\widetilde{w} \widetilde{K^s}{}^\beta \widetilde{T}_{\alpha \beta }. \end{aligned}$$

The first term appears in the right-hand side of (4.24), and the second term can be bounded using (4.22). To bound $S_0$, we take the null decomposition

4.27 $$\begin{aligned} \nabla ^\alpha ({\widetilde{w}}) = {{\widetilde{L}}}^{\alpha }{{\widehat{m}}}^{{{\widetilde{L}}}{\underline{{\widetilde{L}}}}}{\underline{{\widetilde{L}}}}({\widetilde{w}}) + {\underline{{\widetilde{L}}}}^\alpha {{\widehat{m}}}^{{\underline{{\widetilde{L}}}}{{\widetilde{L}}}}{{\widetilde{L}}}({\widetilde{w}}) \end{aligned}$$

The first term is equivalent to ${{\widetilde{L}}}^{\alpha }w'$, and the second term is equivalent to ${\underline{{\widetilde{L}}}}^\alpha {\tau _{0}^{1+2{\overline{\mu }}}}w'$. Expanding out $\widetilde{T}$ in the null decomposition and integrating gives an integral equivalent to $-S_0$ on the right (after applying the Hardy estimate (10.7)), which we may move over to the left-hand side. $\square$

We now bound the perturbations on this energy estimate which arise from the metric. We first define the remainder momentum density tensor

4.28 $$\begin{aligned} P[\phi ]_{rem}^\beta= & {} {\mathfrak {R}}\Big (\tfrac{\overline{D_{\widetilde{K^s}{}}(r^*\phi )}}{r^*} (g^{\beta \gamma } - {{\widehat{m}}}^{\beta \gamma })D_\gamma \phi \nonumber \\{} & {} - \frac{1}{2} \widetilde{K^s}{}^\beta (g^{\gamma \delta } - {{\widehat{m}}}^{\gamma \delta })D_\gamma \phi \overline{D_\delta \phi }\Big ) \end{aligned}$$

and the conical remainder energy

4.29 $$\begin{aligned} C^U_{err}[\phi ](T)= \int _{C_{U,T}}|\partial _\beta ({u^{*{}}})P[\phi ]_{rem}^\beta w|\, dV_C, \end{aligned}$$

### Lemma 4.6

Given (2.24) and a sufficiently regular function $\phi$, the following inequality holds for all *U*:

4.30 $$\begin{aligned}&\left| \int _{[0,T] \times \Sigma _t}{\mathfrak {R}}\left( \left( \Box _g^{{\mathbb {C}}}\phi - \Box _{{\widehat{m}}}^{{\mathbb {C}}}\phi \right) \tfrac{\overline{D_{\widetilde{K^s}{}}(r^*\phi )}}{r^*} w\right) \, dx \, dt\right| \nonumber \\&\quad + \left| \int _{C^U_{ext}}{\mathfrak {R}}\left( \left( \Box _g^{{\mathbb {C}}}\phi - \Box _{{\widehat{m}}}^{{\mathbb {C}}}\phi \right) \tfrac{\overline{D_{\widetilde{K^s}{}}(r^*\phi )}}{r^*} w\right) \, dx \, dt\right| \nonumber \\&\quad \lesssim \varepsilon _g (E_0[\phi ]+S_0[\phi ])(1+|q|+\left\Vert {\textbf{F}}\right\Vert ^{red}_{L^\infty [w]}) + C^U_{err}[\phi ](T). \end{aligned}$$

### Proof

Defining the reduced derivative and wave operator

4.31 $$\begin{aligned} \widetilde{D}_\alpha = \partial _\alpha + iA_\alpha , \quad \widetilde{\Box }^{{\mathbb {C}}}_{{\widehat{m}}}\phi = {{\widehat{m}}}^{\alpha \beta }\widetilde{D}_\alpha \widetilde{D}_\beta \phi , \end{aligned}$$

we have

4.32 $$\begin{aligned} \Box ^{{\mathbb {C}}}_g\phi - \Box ^{{\mathbb {C}}}_{{\widehat{m}}}\phi = (\Box ^{{\mathbb {C}}}_g\phi - \widetilde{\Box }^{{\mathbb {C}}}_{{\widehat{m}}}\phi ) + (\widetilde{\Box }^{{\mathbb {C}}}_{{\widehat{m}}}\phi - \Box ^{{\mathbb {C}}}_{{\widehat{m}}}\phi ) \end{aligned}$$

We will use the harmonic coordinate condition to bound the first term on the right-hand side. The second term can be written in terms of the rapidly decaying Christoffel symbols of ${{\widehat{m}}}$ with respect to the background Minkowski metric. By the inequalities (2.24), (2.5) and (4.10a), and (4.10e), we have the pointwise bound

$$\begin{aligned} |{{\widehat{m}}}^{ab}\widetilde{D}_a\widetilde{D}_b\phi - \Box _{{{\widehat{m}}}}^{{\mathbb {C}}}\phi | \lesssim |\partial A^{\alpha }_ a||\partial \phi | \end{aligned}$$

and consequently

4.33 $$\begin{aligned} \Big \Vert \left( {{\widehat{m}}}^{ab}\widetilde{D}_a\widetilde{D}_b\phi - \Box _{{{\widehat{m}}}}^{{\mathbb {C}}}\phi \right) \tfrac{\overline{D_{\widetilde{K^s}{}}(r^*\phi )}}{r^*} w\Big \Vert _1&\lesssim \varepsilon _g \Big \Vert {\tau _{+}^{s-3/2}}{\tau _{-}^{s}}|\partial \phi |w^{1/2}\Big \Vert _2\nonumber \\&\quad \Big \Vert {\tau _{+}^{-s}}{\tau _{-}^{-1/2-{{\overline{\mu }}}}}\tfrac{\overline{D_{\widetilde{K^s}{}}(r^*\phi )}}{r^*} w^{1/2}\Big \Vert _2 \nonumber \\&\lesssim \varepsilon _g(E_0[\phi ]+S_0[\phi ]) \end{aligned}$$

Next we want to bound

4.34 $$\begin{aligned} \int _0^T{\int _{\Sigma _{t}}}(g^{\alpha \beta } - {{\widehat{m}}}^{\alpha \beta })\widetilde{D}_\alpha \widetilde{D}_\beta \phi \tfrac{\overline{D_{\widetilde{K^s}{}}(r^*\phi )}}{r^*}w \, dx \, dt \end{aligned}$$

in magnitude. Again, we consider $r > \frac{t}{2}$, as proving the results in the far interior are easier. By the divergence theorem,

4.35 $$\begin{aligned} \int _0^T{\int _{\Sigma _{t}}}\partial _\beta \left( P[\phi ]_{rem}^\beta {\widetilde{w}}\right) \, dx \, dt = {\int _{\Sigma _{T}}}P[\phi ]_{rem}^0 {\widetilde{w}}\, dx - {\int _{\Sigma _{0}}}P[\phi ]_{rem}^0 {\widetilde{w}} \, dx\nonumber \\ \end{aligned}$$

We seek to isolate (4.34) and bound all other terms using the energy. First we bound energies of the form

4.36 $$\begin{aligned} \Big |{\int _{\Sigma _{t}}}P[\phi ]_{rem}^0 {\widetilde{w}}\Big |\, dx\, dt\lesssim & {} \Big \Vert \tfrac{\overline{D_{\widetilde{K^s}{}}(r^*\phi )}}{r^*}{\tau _{+}^{-s}}w^{1/2}\Big \Vert _2 \Big \Vert \varepsilon _g {\tau _{+}^{s-1+\delta }} |\widetilde{D}\phi |w^{1/2}\Big \Vert _2\nonumber \\\lesssim & {} \varepsilon _g{{\mathcal {E}}}_0[\phi ](t). \end{aligned}$$

When the derivative falls on $\phi$ or $D\phi$, we get

4.37 $$\begin{aligned}{} & {} {\mathfrak {R}}\left( \tfrac{\overline{D_{\widetilde{K^s}{}}(r^*\phi )}}{r^*}(g-{{\widehat{m}}})^{\beta \gamma }\widetilde{D}_\beta \widetilde{D}_\gamma \phi \right) + \tfrac{\widetilde{K^s}{}(r^*)}{r^*}(g-{{\widehat{m}}})^{\beta \gamma }\widetilde{D}_\gamma \phi \overline{\widetilde{D}_\beta \phi }\nonumber \\{} & {} \quad + {\mathfrak {R}}\left( {\textbf{F}}_{\beta \widetilde{K^s}{}}\phi (g-{{\widehat{m}}})^{\beta \gamma }\overline{\widetilde{D}_\gamma \phi }\right) . \end{aligned}$$

The first term appears in (4.34), and the second and third terms in the corresponding energy integral can be bounded using (4.12), (2.23), and (a virtually identical argument to) (4.22), so in particular

4.38 $$\begin{aligned}{} & {} \Big \Vert \Big (\Big |\tfrac{\widetilde{K^s}{}(r^*)}{r^*}(g-{{\widehat{m}}})^{\beta \gamma }\widetilde{D}_\gamma \phi \overline{\widetilde{D}_\beta \phi }\Big |\nonumber \\{} & {} \quad + \Big |{\textbf{F}}_{\beta \widetilde{K^s}{}}\phi (g-{{\widehat{m}}})^{\beta \gamma }\overline{\widetilde{D}_\gamma \phi }\Big |\Big )w\Big \Vert _1 \lesssim \varepsilon _g{{\mathcal {E}}}_0[\phi ](t) \end{aligned}$$

When the derivative falls on metric terms of (4.28), a null decomposition gives

$$\begin{aligned} \left| (\partial _\beta (g-{{\widehat{m}}})^{\beta \gamma })D_\gamma \phi \right|&\lesssim \varepsilon _g\left( {\tau _{+}^{-1-\gamma +\delta }}{\tau _{-}^{\gamma -1}}|D\phi | + {\tau _{+}^{-1+\delta }}{\tau _{-}^{-1}}|{\overline{D}}\phi |\right) , \\ \left| \widetilde{K^s}{}((g-{{\widehat{m}}})^{\gamma \delta })D_\gamma \phi \overline{D_\delta \phi }\right|&\lesssim \varepsilon _g\left( {\tau _{+}^{2s-2-\gamma +\delta }}{\tau _{-}^{\gamma }}|D\phi |^2 + {\tau _{+}^{2s-2+\delta }}|D\phi ||{\overline{D}}\phi |\right) , \end{aligned}$$

and consequently, using (4.10e), as well as $2s-1-\gamma < 0$, $\gamma < s$,

$$\begin{aligned} \Big \Vert \tfrac{\overline{D_{\widetilde{K^s}{}}(r^*\phi )}}{r^*} (\partial _\beta (g-{{\widehat{m}}})^{\beta \gamma })D_\gamma \phi w\Big \Vert _1&\lesssim \varepsilon _g{{\mathcal {E}}}_0^{1/2}\left( \Big \Vert {\tau _{+}^{s-1-\gamma +\delta }}{\tau _{-}^{\gamma -1/2+{{\overline{\mu }}}}}|D\phi |w^{1/2}\Big \Vert _2\right. \\&\left. \quad + \Big \Vert {\tau _{+}^{s-1+\delta }}{\tau _{-}^{-1/2+{\overline{\mu }}}}|{\overline{D}}\phi |w^{1/2}\Big \Vert _2\right) , \\ \Big \Vert \widetilde{K^s}{}((g-{{\widehat{m}}})^{\gamma \delta })D_\gamma \phi \overline{D_\delta \phi }w\Big \Vert _1&\lesssim \varepsilon _g \Big ({\mathcal {E}}_0 + \Big \Vert {\tau _{-}^{-s}}{\tau _{+}^{s-1/2-{\overline{\mu }}}}|{\overline{D}}\phi | w^{1/2}\\&\quad \Big \Vert _2 \Big \Vert {\tau _{-}^{s}}{\tau _{+}^{-1/2-\delta }}|D\phi |w^{1/2}\Big \Vert _2\Big ). \end{aligned}$$

Therefore,

4.41 $$\begin{aligned}{} & {} \Big \Vert \tfrac{\overline{D_{\widetilde{K^s}{}}(r^*\phi )}}{r^*} (\partial _\beta (g-{{\widehat{m}}})^{\beta \gamma })D_\gamma \phi w\Big \Vert _1 \nonumber \\{} & {} \quad + \Big \Vert \widetilde{K^s}{}((g-{{\widehat{m}}})^{\gamma \delta })D_\gamma \phi \overline{D_\delta \phi }w\Big \Vert _1 \lesssim \varepsilon _g{\mathcal {E}}_0[\phi ]. \end{aligned}$$

When the derivative falls on $\tfrac{1}{r^*}$ or components of $\widetilde{K^s}{}$, we have the terms

4.42 $$\begin{aligned}{} & {} {\mathfrak {R}}\left( (g-{{\widehat{m}}})^{\beta \gamma }\partial _\beta \left( \tfrac{\widetilde{K^s}{}(r^*)}{r^*}\right) {\overline{\phi }}D_\gamma \phi \right. \nonumber \\{} & {} \left. \quad - \frac{1}{2}(\partial _\beta \widetilde{K^s}{}^\beta )(g-{{\widehat{m}}})^{\gamma \delta }D_\gamma \phi \overline{D_\delta \phi } + (\partial _\beta \widetilde{K^s}{}^\alpha )\overline{D_\alpha \phi }(g-{{\widehat{m}}})^{\beta \gamma }D_\gamma \phi \right) .\nonumber \\ \end{aligned}$$

In each a null decomposition along with (2.23) and (2.24) give

4.43a $$\begin{aligned} \left\Vert (g-{{\widehat{m}}})^{\beta \gamma }\partial _\beta \left( \tfrac{\widetilde{K^s}{}(r^*)}{r^*}\right) {\overline{\phi }}D_\gamma \phi w\right\Vert _1&\lesssim \varepsilon _g\left\Vert {\tau _{+}^{2s-3+\delta }}|\phi ||D\phi | w\right\Vert _1 \nonumber \\&\lesssim \varepsilon _g \left\Vert {\tau _{+}^{s-3/2-\delta }}|\phi | w^{1/2}\right\Vert _2\left\Vert {\tau _{+}^{s-3/2+2\delta }}|D\phi |w^{1/2}\right\Vert _2, \end{aligned}$$

4.43b $$\begin{aligned} \left\Vert (\partial _\beta \widetilde{K^s}{}^\beta )H^{\gamma \delta }D_\gamma \phi \overline{D_\delta \phi } w \right\Vert _1&\lesssim \varepsilon _g\left( \left\Vert {\tau _{+}^{2s-2+\delta }}|D\phi ||{\overline{D}}\phi |w\right\Vert _1\right. \nonumber \\&\left. \quad + \left\Vert {\tau _{+}^{2s-2-\gamma +\delta }}{\tau _{-}^{\gamma }}|D\phi |^2w\right\Vert _1\right) \end{aligned}$$

4.43c $$\begin{aligned} \Vert (\partial _\beta \widetilde{K^s}{}^\alpha )\overline{D_\alpha \phi }(g-{{\widehat{m}}})^{\beta \gamma }D_\gamma \phi w\Vert _1&\lesssim \varepsilon _g\left( \left\Vert {\tau _{+}^{2s-2+\delta }}|D\phi ||{\overline{D}}\phi |w\right\Vert _1\right. \nonumber \\&\left. \quad + \left\Vert {\tau _{+}^{2s-2-\gamma +\delta }}{\tau _{-}^{\gamma }}|D\phi |^2w\right\Vert _1\right) . \end{aligned}$$

Therefore,

4.44 $$\begin{aligned}{} & {} \big \Vert \big (\big |(g-{{\widehat{m}}})^{\beta \gamma }\partial _\beta \big (\tfrac{\widetilde{K^s}{}(r^*)}{r^*}\big ){\overline{\phi }}D_\gamma \phi \big | \!+\! |(\partial _\beta \widetilde{K^s}{}^\beta )(g-{{\widehat{m}}})^{\gamma \delta }D_\gamma \phi \overline{D_\delta \phi }| \!\nonumber \\{} & {} \quad + |(\partial _\beta \widetilde{K^s}{}^\gamma )\overline{D_\gamma \phi }(g-{{\widehat{m}}})^{\alpha \beta }D_\beta \phi \big )w\big \Vert _1 \lesssim \varepsilon _g{\mathcal {E}}_0[\phi ] \end{aligned}$$

When the derivative falls on ${\widetilde{w}}$, we use the pointwise estimates

4.45a $$\begin{aligned} |\widetilde{K^s}{}(\widetilde{w})(g-{{\widehat{m}}})^{\alpha \beta } D_\beta \phi \overline{D_\alpha \phi }|&\lesssim \varepsilon _g({\tau _{+}^{2s-1+\delta }}{\tau _{0}^{1+2{{\overline{\mu }}}}} |D\phi ||{\overline{D}}\phi | \nonumber \\&\quad + {\tau _{+}^{2s-1-\gamma +\delta }}{\tau _{0}^{1+2{{\overline{\mu }}}}}{\tau _{-}^{\gamma }} |D\phi |^2)w', \end{aligned}$$

4.45b $$\begin{aligned} \Big |\tfrac{D_{\widetilde{K^s}{}}(r^*\phi )}{r^*}(g-{{\widehat{m}}})^{\alpha \beta }\overline{D_\alpha \phi }\partial _\beta w\Big |&\lesssim \varepsilon _g\Big |{\tau _{+}^{-s}}\tfrac{D_{\widetilde{K^s}{}}(r^*\phi )}{r^*}(w')^{1/2}\nonumber \\&\quad \Big |({\tau _{+}^{s-1+\delta }}{\tau _{0}^{\gamma }}|D\phi |(w')^{1/2} + {\tau _{+}^{s-1+\delta }}|{\overline{D}}\phi |(w')^{1/2}) \end{aligned}$$

Therefore,

4.46 $$\begin{aligned}{} & {} \Big \Vert |\widetilde{K^s}{}(\widetilde{w})(g-{{\widehat{m}}})^{\alpha \beta } D_\beta \phi \overline{D_\alpha \phi }| \nonumber \\{} & {} \quad + \Big |\tfrac{D_{\widetilde{K^s}{}}(r^*\phi )}{r^*}(g-{{\widehat{m}}})^{\alpha \beta }\overline{D_\alpha \phi }\partial _\beta w\Big |\Big \Vert _1 \lesssim \varepsilon _g{\mathcal {E}}_0[\phi ]. \end{aligned}$$

Combining (4.35), (4.36), (4.41), (4.44), (4.45), (4.37), and (4.38) gives our result. $\square$

Finally, Lemmas 4.5 and 4.6 together give Theorem 4.1.

## $L^\infty$ Estimates for ${\textbf{F}}$

We now establish $L^\infty$ estimates on Lie derivatives of the charge-modified field ${\textbf{F}}^1$. These for the most part are identical to estimates in Minkowski space (cf. [18]), modulo our modified frame, as $d{\widetilde{x}}\sim dx$. Our primary tools are Lemma 10.5, which we combine with commutator estimates to convert from derivatives to Lie derivatives. First, we take an estimate which holds for bad components in the extended exterior.

### Lemma 5.1

We have the following uniform estimate on all components of ${\textbf{G}}$:

5.1 $$\begin{aligned} {\tau _{+}^{}}{\tau _{-}^{s+1/2}}|\chi {\textbf{G}}|w^{1/2}\lesssim E_2^{1/2}[{\textbf{G}}] \end{aligned}$$

### Proof

This follows from (10.13) with $\delta _+ = 0$ and $\delta _- = s$, noting

$$\begin{aligned} |{\tau _{-}^{}}{\partial _{r^*}}\phi | \lesssim |{{\widetilde{S}}}\phi | + |{{\widetilde{\Omega }}_{{0r}}}\phi | + |{\partial _{r^*}}\phi |. \end{aligned}$$

then expanding each of these with respect to the Lorentz fields. We have in particular the estimate

5.2 $$\begin{aligned} \left\Vert {\tau _{+}^{}}{\tau _{-}^{1/2+s}}\chi |{\textbf{G}}({{\widetilde{\partial }}_{{\alpha }}}, {{\widetilde{\partial }}_{{\beta }}})|w^{1/2}\right\Vert _{L^\infty _x} \lesssim \sum _{\begin{array}{c} |I| \le 2 \end{array}}\left\Vert {\tau _{-}^{s}}{\widetilde{X}}^I (\chi {\textbf{G}}({{\widetilde{\partial }}_{{\alpha }}}, {{\widetilde{\partial }}_{{\beta }}}))w^{1/2}\right\Vert _{L^2_x}.\nonumber \\ \end{aligned}$$

Then, noting ${\widetilde{X}}^I\chi \lesssim 1$, (2.10) combined with (2.14) gives our result. $\square$

### Lemma 5.2

For all components of ${\textbf{G}}$, we have the following estimate:

5.3 $$\begin{aligned} {\tau _{+}^{s + 3/2}}|(1-\chi ){\textbf{G}}|w^{1/2} \lesssim E_2^{1/2}[{\textbf{G}}]. \end{aligned}$$

The proof is similar, using (10.15) and noting that ${\tau _{-}^{}}\approx {\tau _{+}^{}}$ in the support of $(1-\chi )$.

### Lemma 5.3

For the nice components $\alpha , \rho , \sigma$ of ${\overline{{\textbf{G}}}}$ we have the estimate

5.4 $$\begin{aligned} {\tau _{+}^{s+1}}{\tau _{-}^{1/2}}|{\overline{{\textbf{G}}}}|w^{1/2}\lesssim E_2^{1/2}[{\textbf{G}}]. \end{aligned}$$

### Proof

By (5.2), it suffices to show this for $\chi {\overline{{\textbf{G}}}}$. We use Lemma 2.1. We show this for $\sigma$, as the proof for the other components is identical.

5.5 $$\begin{aligned} {\tau _{+}^{s+1}}{\tau _{-}^{1/2}}|\chi \sigma [{\textbf{G}}]|w^{1/2}\lesssim \sum _{\begin{array}{c} |I| \le 2 \end{array}}\left\Vert {\tau _{+}^{s}} {\widetilde{X}}^I(\chi \sigma [{\textbf{G}}]) w^{1/2}\right\Vert _{L^2_x}. \end{aligned}$$

Repeated application of Lemma 2.1 gives the desired estimate. $\square$

We now look at the $L^2(L^\infty )$ estimate for $\alpha$.

### Lemma 5.4

Given a form ${\textbf{G}}$ with sufficient decay, we have the bound

5.6 $$\begin{aligned} \left\Vert {\tau _{+}^{s+1}}{\tau _{-}^{1/2}}\alpha [{\textbf{G}}](w')^{1/2}\right\Vert _{L^2_t(L^\infty _x)} \lesssim S_2[{\textbf{G}}](T). \end{aligned}$$

### Proof

We as usual take our Sobolev estimate in the extended exterior

5.7 $$\begin{aligned} \left\Vert {\tau _{+}^{s+1}}{\tau _{-}^{1/2}}\chi \alpha [{\textbf{G}}](w')^{1/2}\right\Vert ^2_{L^\infty _x} \lesssim \sum _{\begin{array}{c} |I| \le 2 \end{array}}\left\Vert {\tau _{+}^{s}}{\widetilde{X}}^I(\chi \alpha [{\textbf{G}}])(w')^{1/2}\right\Vert ^2_{L^2_x}. \end{aligned}$$

We can use the usual expansion in terms of Lie derivatives, followed by the commutator estimate 2.1, and it follows that this is contained in $S_2[{\textbf{G}}](T)$. We combine this with the interior estimate

5.8 $$\begin{aligned} \left\Vert {\tau _{+}^{3/2+s}}|{\textbf{G}}_{\alpha \beta }|(w')^{1/2}\right\Vert _{L^\infty _x}^2\lesssim \sum _{|I| \le 2}\left\Vert {\tau _{+}^{s}}{\widetilde{X}}^I((1-\chi ){\textbf{G}}_{\alpha \beta })(w')^{1/2}\right\Vert _{L^2_x}^2 \end{aligned}$$

coming from (10.15). Integrating in time gives our result. $\square$

We can combine these estimates as follows:

### Lemma 5.5

For any two-form ${\textbf{G}}$ with zero charge and sufficient decay, the following estimate holds on [0, *T*]:

5.9 $$\begin{aligned}{} & {} {\tau _{+}^{}}{\tau _{-}^{s+1/2}}|{\underline{\alpha }}[{\textbf{G}}]| + {\tau _{+}^{s+1}}{\tau _{-}^{1/2}}(|\alpha [{\textbf{G}}]| + |\rho [{\textbf{G}}]|+|\sigma [{\textbf{G}}]|) \nonumber \\{} & {} \quad + \left\Vert {\tau _{+}^{s+1}}{\tau _{-}^{1/2}}\alpha [{\textbf{G}}](w')^{1/2}\right\Vert _{L^2_tL^\infty _x} \lesssim {\mathcal {E}}_2[{\textbf{G}}](T). \end{aligned}$$

We will use this to bound components of ${\textbf{F}}^1$ and its Lie derivatives.

Before we proceed, we mention one auxiliary estimate, which will give us more precise bounds for the component $\alpha$. We recall the conical energy

$$\begin{aligned} C_0[{\textbf{G}}](T) = \sup _{{u^{*{}}}}\int _{\{C({u^{*{}}})\} \cap \{t \in [0,T]\} }\left( \tfrac{1}{2} {\tau _{+}^{2s}}T(\nabla u^*, {{\widetilde{L}}}) + \tfrac{1}{2}{\tau _{-}^{2s}}T[{\textbf{G}}](\nabla u^*, {\underline{{\widetilde{L}}}})\right) w \, dVC({u^{*{}}}). \end{aligned}$$

Then, (3.21) combined with the null decomposition of *g* gives

5.10a $$\begin{aligned} \left| T[{\textbf{G}}](\nabla u^*, {{\widetilde{L}}})-|\alpha |^2\right|&\lesssim \varepsilon _g|{\alpha }|^2 + \varepsilon _g{\tau _{+}^{-1-\gamma +\delta }}{\tau _{-}^{\gamma }}|{\overline{{\textbf{G}}}}|^2\nonumber \\&\quad + \varepsilon _g^2{\tau _{-}^{2\gamma }}{\tau _{+}^{-2-2\gamma +2\delta }}|{\textbf{G}}|^2, \end{aligned}$$

5.10b $$\begin{aligned} \left| T[{\textbf{G}}](\nabla u^*, {\underline{{\widetilde{L}}}})-(|\rho |^2+|\sigma |^2)\right|&\lesssim \varepsilon _g|{\overline{{\textbf{G}}}}|^2 + \varepsilon _g{\tau _{-}^{\gamma }}{\tau _{+}^{-1-\gamma +\delta }}|{\textbf{G}}|^2. \end{aligned}$$

Therefore, for sufficiently small $\varepsilon _g$, and for *C* independent of $\varepsilon _g$, the inequality $2\,s + \delta < 1+\gamma$ gives:

5.11 $$\begin{aligned} \frac{1}{2}{\tau _{+}^{2s}}T[{\textbf{G}}](\nabla u^*, {{\widetilde{L}}}) + \frac{1}{2}{\tau _{-}^{2s}}T[{\textbf{G}}](\nabla u^*, {\underline{{\widetilde{L}}}})\ge & {} 1/2({\tau _{+}^{2s}}|\alpha |^2 + {\tau _{-}^{2s}}(|\rho |^2+|\sigma |^2)) \nonumber \\{} & {} - C\varepsilon _g{\tau _{-}^{2s+\gamma }}{\tau _{+}^{-1-\gamma +\delta }}|{\textbf{G}}|^2. \end{aligned}$$

We may bound the last term by

5.12 $$\begin{aligned} \varepsilon _g{\tau _{-}^{2s+\gamma }}{\tau _{+}^{-1-\gamma +\delta }}|{\textbf{G}}|^2 \lesssim \varepsilon _g {\tau _{-}^{\gamma -1}}{\tau _{+}^{-3-\gamma +\delta }}{\mathcal {E}}_2[{\textbf{G}}](T). \end{aligned}$$

It follows that

5.13 $$\begin{aligned} \sup _{{u^{*{}}}}\int _{\{C({u^{*{}}})\} \cap \{t \in [0,T]\} }\varepsilon _g{\tau _{-}^{2s+\gamma }}{\tau _{+}^{-1-\gamma +\delta }}|{\textbf{G}}|^2 \lesssim \varepsilon _g{\mathcal {E}}_2[{\textbf{G}}](T). \end{aligned}$$

The estimate (3.40) follows. In order to further bound nice components, we take the estimate

5.14 $$\begin{aligned}{} & {} \left\Vert {\tau _{+}^{3/2+s}}\alpha [{\textbf{G}}] w\right\Vert _{L^\infty (C_{{u^{*{}}}})} \nonumber \\{} & {} \quad \lesssim \sum _{\begin{array}{c} |I|, |J| \le 1\\ {\widetilde{X}}\in \{{\underline{u}^{*{}}}{{\widetilde{L}}}, {\mathbb {O}}\}, {\widetilde{Y}}\in {\mathbb {O}} \end{array}}\left\Vert {\tau _{+}^{2s}} \left( \alpha [{\mathcal {L}}_{{\widetilde{X}}}^I{\mathcal {L}}_{{\widetilde{Y}}}^J {\textbf{G}}] + {\tau _{+}^{2s}}{\tau _{0}^{}}\left( \rho [{\mathcal {L}}_{{\widetilde{X}}}^I{\mathcal {L}}_{{\widetilde{Y}}}^J {\textbf{G}}] + \sigma [{\mathcal {L}}_{{\widetilde{X}}}^I{\mathcal {L}}_{{\widetilde{Y}}}^J {\textbf{G}}]\right) \right) w\right\Vert _{L^2(C_{u^*})}.\nonumber \\ \end{aligned}$$

Applying Eq. (5.13) gives us the estimate

5.15 $$\begin{aligned} |{\tau _{+}^{3/2+s}}\alpha [{\textbf{G}}] w^{1/2}| \lesssim {\mathcal {E}}^{1/2}_4[{\textbf{G}}](T). \end{aligned}$$

We can combine these to get the following:

### Theorem 5.6

For any two-form ${\textbf{G}}$ with zero associated charge and sufficient decay, the following estimates hold:

5.16a $$\begin{aligned}&{\tau _{+}^{}}{\tau _{-}^{s+1/2}}|{\underline{\alpha }}[{\textbf{G}}]|w + {\tau _{+}^{s+1}}{\tau _{-}^{1/2}}(|\alpha [{\textbf{G}}]| + |\rho [{\textbf{G}}]|+|\sigma [{\textbf{G}}]|)w \nonumber \\&\quad + \left\Vert {\tau _{+}^{s+1}}{\tau _{-}^{1/2}}\alpha [{\textbf{G}}](w')^{1/2}\right\Vert _{L^2_t(L^\infty _x)} \lesssim {\mathcal {E}}^{1/2}_2[{\textbf{G}}](T), \end{aligned}$$

5.16b $$\begin{aligned}&|{\tau _{+}^{3/2+s}}\alpha [{\textbf{G}}] w^{1/2}| \lesssim {\mathcal {E}}^{1/2}_4[{\textbf{G}}](T). \end{aligned}$$

We have the $L^\infty$ estimates on derivatives:

5.17a $$\begin{aligned}&{\tau _{+}^{}}{\tau _{-}^{s+1/2}}|{\underline{\alpha }}[{\mathcal {L}}_{{\widetilde{X}}}^I{\textbf{G}}]| + {\tau _{+}^{s+1}}{\tau _{-}^{1/2}}(|\rho [{\mathcal {L}}_{{\widetilde{X}}}^I{\textbf{G}}]|+|\sigma [{\mathcal {L}}_{{\widetilde{X}}}^I{\textbf{G}}]|) \nonumber \\&\quad + |{\tau _{+}^{3/2+s}}\alpha [{\mathcal {L}}_{{\widetilde{X}}}^I{\textbf{G}}] w^{1/2}| \lesssim {\mathcal {E}}^{1/2}_{|I|+4}[{\textbf{G}}](T) \end{aligned}$$

5.17b $$\begin{aligned}&\left\Vert {\tau _{+}^{s+1}}{\tau _{-}^{1/2}}\alpha [{\mathcal {L}}_{{\widetilde{X}}}^I{\textbf{G}}](w')^{1/2}\right\Vert _{L^2_t(L^\infty _x)} \lesssim {\mathcal {E}}^{1/2}_{|I|+2}[{\textbf{G}}](T) \end{aligned}$$

## $L^\infty$ Estimates for $\varvec{\phi }$

We now establish analogous $L^\infty$ estimates on $\phi$. Before doing so, we show some auxiliary bounds on quantities relating to ${\textbf{F}}$, which will also be useful later. For an arbitrary two-form ${\textbf{G}}$, we can expand ${\widetilde{X}}$ in our null frame to get the pointwise bounds

6.1a $$\begin{aligned} |{\textbf{G}}({\widetilde{X}}, {{\widetilde{L}}})|&\lesssim {\tau _{+}^{}}|\alpha [{\textbf{G}}]| + {\tau _{-}^{}}|\rho [{\textbf{G}}]|, \end{aligned}$$

6.1b $$\begin{aligned} |{\textbf{G}}({\widetilde{X}}, {{\widetilde{S}}_{i}})|&\lesssim {\tau _{+}^{}}(|\alpha [{\textbf{G}}]|+|\sigma [{\textbf{G}}]|) + {\tau _{-}^{}}|{\underline{\alpha }}[{\textbf{G}}]|, \end{aligned}$$

6.1c $$\begin{aligned} |{\textbf{G}}({\widetilde{X}}, {\underline{{\widetilde{L}}}})|&\lesssim {\tau _{+}^{}}(|\rho [{\textbf{G}}]|+|{\underline{\alpha }}[{\textbf{G}}]|). \end{aligned}$$

The identity (2.43a) gives

6.2 $$\begin{aligned} |({\mathcal {L}}_{{\widetilde{X}}}^ID\phi )_a|\lesssim |\widetilde{D}_a D_{{\widetilde{X}}}^I\phi | + \sum _{|I_1|+|I_2|+1\le |I|}|D_{{\widetilde{X}}}^{I_1}\phi ||({\mathcal {L}}_{{\widetilde{X}}}^{I_2}{\textbf{F}})({\widetilde{\partial }}_a, {\widetilde{X}}_1)|. \end{aligned}$$

We now show $L^2$ and $L^\infty$ spacetime norms on components of ${\mathcal {L}}_{{\widetilde{X}}}^ID\phi$, as these crop up naturally in the current norm. We hope to be able to approximate them in the same way as $|DD_X^I\phi |$. We first split ${\textbf{F}}= {\textbf{F}}^0+{\textbf{F}}^1$. If $k-4$ or fewer derivatives fall on ${\textbf{F}}^1$, or if any number of derivatives fall on ${\textbf{F}}^0$, (2.50) and (5.16) give

$$\begin{aligned}&\sum _{|I|\le k}|\alpha [{\mathcal {L}}_{{\widetilde{X}}}^I{\textbf{F}}^0]| + \sum _{|J|\le k-4}|\alpha [{\mathcal {L}}_{{\widetilde{X}}}^J{\textbf{F}}^1]| \\&\quad \lesssim {{\mathcal {E}}}_k(T)^{1/2}{\tau _{+}^{-3/2-s}}{\left\langle {(r^*-t)_+}\right\rangle }^{s-1/2}\\&\sum _{|I|\le k}(|\rho [{\mathcal {L}}_{{\widetilde{X}}}^I{\textbf{F}}^0]| + |\sigma [{\mathcal {L}}_{{\widetilde{X}}}^I{\textbf{F}}^0]|) + \sum _{|J|\le k-4}(|\rho [{\mathcal {L}}_{{\widetilde{X}}}^J{\textbf{F}}^1]| + |\sigma [{\mathcal {L}}_{{\widetilde{X}}}^J{\textbf{F}}^1]|)\\&\quad \lesssim {{\mathcal {E}}}_k(T)^{1/2}{\tau _{+}^{-1-s}}{\tau _{-}^{-1/2}}{\left\langle {(r^*-t)_+}\right\rangle }^{s-1/2}\\&\sum _{|I|\le k}|{\underline{\alpha }}[{\mathcal {L}}_{{\widetilde{X}}}^I{\textbf{F}}^0]| + \sum _{|J|\le k-4}|{\underline{\alpha }}[{\mathcal {L}}_{{\widetilde{X}}}^I{\textbf{F}}^1]| \\&\quad \lesssim {{\mathcal {E}}}_k(T)^{1/2}{\tau _{+}^{-1}}{\tau _{-}^{-1/2-s}}{\left\langle {(r^*-t)_+}\right\rangle }^{s-1/2} \end{aligned}$$

This combined with (6.1) gives

6.4a $$\begin{aligned} \sum _{|I|\le k-1}|({\mathcal {L}}_{{\widetilde{X}}}^I{\textbf{F}}^0)_{{{\widetilde{X}}}_1{{\widetilde{L}}}}| + \sum _{|J|\le k-5}|({\mathcal {L}}_{{\widetilde{X}}}^J{\textbf{F}}^1)_{{{\widetilde{X}}}_1{{\widetilde{L}}}}|&\lesssim {{\mathcal {E}}}_k(T)^{1/2}{\tau _{+}^{-1/2-s}}{\left\langle {(r^*-t)_+}\right\rangle }^{s-1/2} \end{aligned}$$

6.4b $$\begin{aligned} \sum _{|I|\le k-1}|({\mathcal {L}}_{{\widetilde{X}}}^I{\textbf{F}}^0)_{{{\widetilde{X}}}_1{{\widetilde{S}}_{i}}}| + \sum _{|J|\le k-5}({\mathcal {L}}_{{\widetilde{X}}}^J{\textbf{F}}^1)_{{{\widetilde{X}}}_1{{\widetilde{S}}_{i}}}|&\lesssim {{\mathcal {E}}}_k(T)^{1/2}{\tau _{+}^{-s}}{\tau _{-}^{-1/2}}{\left\langle {(r^*-t)_+}\right\rangle }^{s-1/2} \end{aligned}$$

6.4c $$\begin{aligned} \sum _{|I|\le k-1}|({\mathcal {L}}_{{\widetilde{X}}}^I{\textbf{F}}^0)_{{{\widetilde{X}}}_1{\underline{{\widetilde{L}}}}}| + \sum _{|J|\le k-5}|({\mathcal {L}}_{{\widetilde{X}}}^J{\textbf{F}}^1)_{{{\widetilde{X}}}_1{\underline{{\widetilde{L}}}}}|&\lesssim {{\mathcal {E}}}_k(T)^{1/2}{\tau _{-}^{-1/2-s}}{\left\langle {(r^*-t)_+}\right\rangle }^{s-1/2} \end{aligned}$$

We now establish our estimates on $\phi$, which follow from Theorem 10.5, with consideration for commutators, and will be conducted in the $\{\widetilde{\partial }_a\}$ frame. We first have

6.5 $$\begin{aligned}{} & {} {\tau _{+}^{2}}{\tau _{-}^{2s+1}}|D\phi |^2w + {\tau _{+}^{2s+2}}{\tau _{-}^{}}\sum _{|I|\le 1}\left| \tfrac{D_{{\widetilde{X}}}\phi }{{\tau _{+}^{}}}\right| ^2w \nonumber \\{} & {} \quad \lesssim \sum _{|I|\le 2}\left\Vert {\tau _{-}^{s}}D_{{\widetilde{X}}}^I D\phi w^{1/2}\right\Vert ^2_{L^2_x} + \sum _{|I|\le 3}\left\Vert {\tau _{+}^{s-1}}D_{{\widetilde{X}}}^I\phi w^{1/2}\right\Vert ^2_{L^2_x}. \end{aligned}$$

Expanding ${\widetilde{X}}$ in our null decomposition allows us to bound the second norm on the right by $E_2[\phi ](T)$, so we only need to deal with the first term. We reduce this to the commutator, noting

$$\begin{aligned} \sum _{|I|\le 2 }\left\Vert {\tau _{-}^{s}}DD_{{\widetilde{X}}}^I\phi w^{1/2}\right\Vert _{L^2_x} \lesssim (E_2[\phi ](T))^{1/2}. \end{aligned}$$

We look at the case $|I| = 2$, as other cases are easier. First, we have the identity

6.6 $$\begin{aligned}{}[D_{{\widetilde{X}}_1}D_{{\widetilde{X}}_2}, {{\widetilde{D}}_{a}}]\phi= & {} D_{{\widetilde{X}}_1}(i{\textbf{F}}({\widetilde{X}}_2, {{\widetilde{\partial }}_{{a}}})\phi )+ i{\textbf{F}}({\widetilde{X}}_1, {{\widetilde{\partial }}_{{a}}})D_{{\widetilde{X}}_2}\phi \nonumber \\{} & {} + D_{[{\widetilde{X}}_1, {{\widetilde{\partial }}_{{a}}}]}D_{{\widetilde{X}}_2}\phi + D_{{\widetilde{X}}_1}D_{[{\widetilde{X}}_2, {\widetilde{\partial }}_a]}\phi . \end{aligned}$$

The third and fourth terms are bounded by the energy, and a reduction of order, respectively. We show the bound for the first term, as the second term follows from an easier argument. The identity (2.14) gives:

6.7 $$\begin{aligned} D_{{{\widetilde{X}}}_1}(i{\textbf{F}}({{\widetilde{X}}}_2, {{\widetilde{\partial }}_{{\alpha }}})\phi )= & {} i{\textbf{F}}({{\widetilde{X}}}_2, {{\widetilde{\partial }}_{{\alpha }}})D_{{{\widetilde{X}}}_1}\phi + i({\mathcal {L}}_{{{\widetilde{X}}}_1}{\textbf{F}})({{\widetilde{X}}}_2, {{\widetilde{\partial }}_{{\alpha }}})\phi \nonumber \\{} & {} + i{\textbf{F}}([{{\widetilde{X}}}_1, {{\widetilde{X}}}_2], {{\widetilde{\partial }}_{{\alpha }}})\phi + i{\textbf{F}}({{\widetilde{X}}}_2, [{{\widetilde{X}}}_1, {{\widetilde{\partial }}_{{\alpha }}}])\phi . \end{aligned}$$

Therefore,

6.8 $$\begin{aligned}{} & {} \sum \left\Vert {\tau _{-}^{s}}D_{{\widetilde{X}}_1}(i{\textbf{F}}({\widetilde{X}}_2, {{\widetilde{\partial }}_{{\alpha }}})\phi )w^{1/2}\right\Vert _{L^2_x}\nonumber \\{} & {} \quad \lesssim \sum _{|I|\le 1}|{\tau _{-}^{}}{\mathcal {L}}_{{\widetilde{X}}_1}{\textbf{F}}({\widetilde{X}}_2, {{\widetilde{\partial }}_{{a}}})|\sum _{|I|\le 1 }\Vert {\tau _{-}^{s-1}}D_{{\widetilde{Y}}}\phi w^{1/2}\Vert _{L^2_x}. \end{aligned}$$

An application of Lemma 10.8 with $p = 2\,s-2$, $q = 0$ gives us

6.9 $$\begin{aligned} \left\Vert {\tau _{-}^{s-1}}D_{{\widetilde{Y}}}\phi w^{1/2}\right\Vert _{L^2_x} \lesssim (E_1[\phi ](T))^{1/2}. \end{aligned}$$

Therefore,

6.10 $$\begin{aligned}{} & {} \sum _{|I|\le 2}\left\Vert {\tau _{-}^{s}}D_{{\widetilde{X}}}^I D\phi w^{1/2}\right\Vert ^2_{L^2_x} + \sum _{|I|\le 3}\left\Vert {\tau _{-}^{s}}{\tau _{+}^{-1}}D_{{\widetilde{X}}}^I\phi w^{1/2}\right\Vert ^2_{L^2_x} \nonumber \\{} & {} \quad \lesssim \left( 1+{\tau _{-}^{}}\sum _{|I|\le 1}| {\mathcal {L}}_{{\widetilde{X}}}{\textbf{F}}|\right) ^2E_2[\phi ](T). \end{aligned}$$

We have our first Lemma:

### Lemma 6.1

For a function $\phi$ defined on $[0,T] \times \Sigma _t$ with suitable regularity,

6.11 $$\begin{aligned}{} & {} {\tau _{+}^{}}{\tau _{-}^{s+1/2}}|D\phi |w^{1/2} + {\tau _{+}^{s+1}}{\tau _{-}^{1/2}}\sum _{|I|\le 1 }\left| \tfrac{D_{{\widetilde{X}}}\phi }{{\tau _{+}^{}}}\right| w^{1/2}\nonumber \\{} & {} \quad \lesssim \left( 1+{\tau _{-}^{}}\sum _{|I|\le 1}| {\mathcal {L}}_{{\widetilde{X}}}{\textbf{F}}|\right) (E_2[\phi ](T))^{1/2}. \end{aligned}$$

This estimate will be used to bound $D_{\underline{{\widetilde{L}}}}\phi$ for $r^* \ge t/2$, and all derivatives when $r^* \le t/2$ and will be a base for later $L^\infty$ estimates. We now take a look at our better derivatives. We will prove this for a general vector field *U*, noting that the estimate will not necessarily be finite unless $U\in \{{{\widetilde{L}}}, {{\widetilde{S}}_{1}}, {{\widetilde{S}}_{2}}\}$. Again, Theorem 10.5 gives

6.12 $$\begin{aligned} \left\Vert {\tau _{+}^{2+2s}}{\tau _{-}^{1}}|D_{U}\phi |^2w \right\Vert _{L^\infty _x} \lesssim \sum _{|I|\le 2}\left\Vert {\tau _{+}^{s}}D_{{\widetilde{X}}}^I D_{U}\phi w^{1/2}\right\Vert ^2_{L^2_x}. \end{aligned}$$

We recall the identities

6.13 $$\begin{aligned}{}[D_{{\widetilde{X}}_1}D_{{\widetilde{X}}_2}, D_{U}]\phi= & {} D_{{\widetilde{X}}_1}(i{\textbf{F}}({\widetilde{X}}_2, {U})\phi )+ D_{{\widetilde{X}}_1}D_{[{\widetilde{X}}_2, {U}]}\phi \nonumber \\{} & {} + i{\textbf{F}}({\widetilde{X}}_1, {U})D_{{\widetilde{X}}_2}\phi + D_{[{\widetilde{X}}_1, U]}D_{{\widetilde{X}}_2}\phi , \end{aligned}$$

for which a straightforward argument (using (2.10) and (2.11)) again allows us to reduce to bounding the first term, and

6.14 $$\begin{aligned} D_{{\widetilde{X}}_1}(i{\textbf{F}}({\widetilde{X}}_2, U)\phi )= & {} i{\textbf{F}}({\widetilde{X}}_2, {U})D_{{\widetilde{X}}_1}\phi + i({\mathcal {L}}_{{\widetilde{X}}_1}{\textbf{F}})({\widetilde{X}}_2, {U})\phi \nonumber \\{} & {} + i{\textbf{F}}([{\widetilde{X}}_1, {\widetilde{X}}_2], {U})\phi + i{\textbf{F}}({\widetilde{X}}_2, [{\widetilde{X}}_1, {U}])\phi . \end{aligned}$$

For $U\in \{{{\widetilde{L}}}, {{\widetilde{S}}_{1}}, {{\widetilde{S}}_{2}}\}$, the commutator identities (2.10) and (2.11) and a null decomposition give

6.15 $$\begin{aligned} |D_{{\widetilde{X}}_1}(i{\textbf{F}}({\widetilde{X}}_2, U)\phi )|\lesssim & {} \sum _{|I|\le 1 }\left( {\tau _{+}^{}}(|{\alpha }[{\mathcal {L}}_{{\widetilde{X}}}{\textbf{F}}]| + |\rho [{\mathcal {L}}_{{\widetilde{X}}}{\textbf{F}}]| \right. \nonumber \\{} & {} \left. + |\sigma [{\mathcal {L}}_{{\widetilde{X}}}{\textbf{F}}]|) + {\tau _{-}^{}}|{\underline{\alpha }}[{\mathcal {L}}_{{\widetilde{X}}}{\textbf{F}}]|\right) \sum _{|I|\le 1}|D_{{\widetilde{Y}}}\phi | \end{aligned}$$

Recalling the definition (4.9), we have the inequality

6.16 $$\begin{aligned}{} & {} {\tau _{+}^{s}}{\tau _{-}^{1-s}}\left( {\tau _{+}^{}}(|{\alpha }[{\mathcal {L}}_{{\widetilde{X}}}{\textbf{F}}]| + |\rho [{\mathcal {L}}_{{\widetilde{X}}}{\textbf{F}}]| + |\sigma [{\mathcal {L}}_{{\widetilde{X}}}{\textbf{F}}]| + {\tau _{-}^{}}|{\underline{\alpha }}[{\mathcal {L}}_{{\widetilde{X}}}{\textbf{F}}]|\right) \nonumber \\{} & {} \quad \lesssim |q|+\left\Vert {\mathcal {L}}_{{\widetilde{X}}}{\textbf{F}}\right\Vert ^{red}_{L^\infty [w]}. \end{aligned}$$

It follows that

6.17 $$\begin{aligned} \sum _{|I|\le 2 }\left\Vert {\tau _{+}^{s}}D_{{\widetilde{X}}_1}(i{\textbf{F}}({\widetilde{X}}_2, D_{U})\phi w^{1/2}\right\Vert ^2_{L^2_x}\lesssim & {} \left( 1+\sum _{|I|\le 1}\left\Vert {\mathcal {L}}_{{\widetilde{X}}}{\textbf{F}}\right\Vert ^{red}_{L^\infty [w]}\right) ^2\nonumber \\{} & {} \sum _{|I|\le 1 }\left\Vert {\tau _{-}^{s-1}}D_{{\widetilde{Y}}}\phi w^{1/2}\right\Vert _{L^2_x}^2.\nonumber \\ \end{aligned}$$

Combining this with Lemma 10.8 gives us our second estimate:

### Lemma 6.2

For a function $\phi$ defined on $[0,T] \times \Sigma _t$ with suitable regularity, and for $U\in \{{{\widetilde{L}}}, {{\widetilde{S}}_{1}}, {{\widetilde{S}}_{2}}\}$,

6.18 $$\begin{aligned} {\tau _{+}^{s+1}}{\tau _{-}^{1/2}}|D_{U}\phi |w^{1/2}\lesssim \left( 1+|q|+\sum _{|I|\le 1}\left\Vert {\mathcal {L}}_{{\widetilde{X}}}{\textbf{F}}\right\Vert ^{red}_{L^\infty [w]}\right) (E_2[\phi ](T))^{1/2}.\nonumber \\ \end{aligned}$$

We can now take a nicer estimate on $\phi$. Our time-slice Sobolev estimate, (10.16), gives us:

6.19 $$\begin{aligned} {\tau _{+}^{}}{\tau _{-}^{s-1/2}}|\phi |w^{1/2} \lesssim \sum _{|I|\le 1 }\left\Vert {\tau _{-}^{s-1}}D_{{\widetilde{X}}}^I\phi w^{1/2}\right\Vert _{L^2_x}. \end{aligned}$$

Lemma 10.8 gives the following estimate:

### Lemma 6.3

For a function $\phi$ defined on $[0,T] \times \Sigma _t$ with suitable regularity,

6.20 $$\begin{aligned} {\tau _{+}^{}}{\tau _{-}^{s-1/2}}|\phi |w^{1/2} \lesssim E_2^{1/2}[\phi ](T). \end{aligned}$$

We now take an $L^2(t)L^\infty (x)$ estimate for the nice component $r^{*-1}D_{{{\widetilde{L}}}}(r^*\phi )$ in the extended exterior region $r^* > t/2$. We can rewrite Theorem 10.5 and integrate to get

6.21 $$\begin{aligned}&\left\Vert {\tau _{+}^{s+1}}{\tau _{-}^{1/2}} \tfrac{D_{{{\widetilde{L}}}}(r^*\phi )}{r^*}(w')^{1/2}\right\Vert ^2_{L^2_t(L^\infty _x)}\nonumber \\&\lesssim \sum _{\begin{array}{c} |I|, |J| \le 1 \\ \widetilde{V}\in \{{\partial _{r^*}}, {{\widetilde{S}}}, {{\widetilde{\Omega }}_{{0r^*}}}, {{\widetilde{\Omega }}_{{ij}}}\}\widetilde{U} \in \{{{\widetilde{\Omega }}_{{ij}}}\} \end{array}}\left\Vert {\tau _{+}^{s}}D_{\widetilde{V}}^ID_{\widetilde{U}}^J\Big (\tfrac{D_{{{\widetilde{L}}}}(r^*\phi )}{r^*}\Big )(w')^{1/2}\right\Vert ^2_2, \end{aligned}$$

where we recall the bound

$$\begin{aligned} |{\tau _{-}^{}}{\partial _{r^*}}\phi | \lesssim |{\partial _{r^*}}\phi | + |{{\widetilde{\Omega }}_{{0r^*}}}\phi | + |{{\widetilde{S}}}\phi |. \end{aligned}$$

For a vector field $\widetilde{W}$, we have with the commutator relation

6.22 $$\begin{aligned} \left[ D_{\widetilde{W}}, \frac{1}{r^*}D_{{\widetilde{L}}}(r^*\cdot )\right] \phi = D_{[\widetilde{W}, {{\widetilde{L}}}]}\phi + i{\textbf{F}}(\widetilde{W}, {{\widetilde{L}}})\phi + \widetilde{W}\left( \tfrac{1}{r^*}\right) \phi \end{aligned}$$

from which it follows that, for $\widetilde{V}$ as in (6.21),

6.23 $$\begin{aligned} \left[ D_{{\widetilde{V}}}D_{{\widetilde{U}}}, \tfrac{1}{r^*}D_{{\widetilde{L}}}(r^*\cdot )\right] \phi&= D_{{\widetilde{V}}}\left( D_{[{\widetilde{U}}, {{\widetilde{L}}}]}\phi + i{\textbf{F}}({\widetilde{U}}, {{\widetilde{L}}})\phi + {\widetilde{U}}\left( \tfrac{1}{r^*}\right) \phi \right) \nonumber \\&\quad + D_{[{\widetilde{V}}, {{\widetilde{L}}}]}D_{{\widetilde{U}}}\phi + i({\textbf{F}}({\widetilde{V}}, {{\widetilde{L}}})D_{{\widetilde{U}}}\phi ) + {\widetilde{V}}\left( \tfrac{1}{r^*}\right) D_{{\widetilde{U}}}\phi . \end{aligned}$$

Recalling the identities (2.11), we may simplify

6.24 $$\begin{aligned} \left[ D_{\widetilde{V}}D_{{{\widetilde{\Omega }}_{{ij}}}}, \tfrac{1}{r^*}D_{{\widetilde{L}}}(r^*\cdot )\right] \phi= & {} iD_{\widetilde{V}}({\textbf{F}}({{\widetilde{\Omega }}_{{ij}}}, {{\widetilde{L}}})\phi ) + D_{[\widetilde{V}, {{\widetilde{L}}}]}D_{{{\widetilde{\Omega }}_{{ij}}}}\phi \nonumber \\{} & {} +i{\textbf{F}}(\widetilde{V}, {{\widetilde{L}}})D_{{{\widetilde{\Omega }}_{{ij}}}}\phi + \widetilde{V}\Big (\tfrac{1}{r^*}\Big )D_{{{\widetilde{\Omega }}_{{ij}}}}\phi . \end{aligned}$$

We bound everything in groups. First, setting $\widetilde{V} = {{\widetilde{\Omega }}_{{0r^*}}}$, expanding in terms of ${{\widetilde{\Omega }}_{{0i}}}$ (using the linearity of the Lie derivative), and taking (6.14), (2.10) and (2.11), gives

6.25 $$\begin{aligned} |D_{\widetilde{V}}({\textbf{F}}({{\widetilde{\Omega }}_{{ij}}}, {{\widetilde{L}}})\phi )|\lesssim \sum _{|I|+|J|\le 1}{\tau _{+}^{}}\alpha [{\mathcal {L}}_{{{\widetilde{X}}}}^I{\textbf{F}}]|D_{{\widetilde{Y}}}^J\phi | \end{aligned}$$

Additionally, a null decomposition gives

6.26 $$\begin{aligned} |{\textbf{F}}(\widetilde{V}, {{\widetilde{L}}})|\lesssim {\tau _{-}^{}}|\rho |. \end{aligned}$$

For all $\widetilde{V}$, we use (2.10) and (2.11), and the null decomposition (2.8), to get

6.27 $$\begin{aligned} D_{[\widetilde{V}, {{\widetilde{L}}}]}D_{{{\widetilde{\Omega }}_{{ij}}}}\phi = C_{\widetilde{V}} D_{{{\widetilde{L}}}}D_{{{\widetilde{\Omega }}_{{ij}}}}\phi , \end{aligned}$$

where $C_{\widetilde{V}} = -1$ for $\widetilde{V} = {{\widetilde{S}}}$ or ${{\widetilde{\Omega }}_{{0r^*}}}$ and 0 for all other fields. Consequently, in the domain of $\chi$ we have

6.28 $$\begin{aligned} \Big |\widetilde{V}\Big (\tfrac{1}{r^*}\Big ) - \tfrac{C_{\widetilde{V}}}{r^*}\Big |\lesssim & {} \tfrac{{\tau _{-}^{}}}{r^{*2}}, \quad \Big |D_{[\widetilde{V}, {{\widetilde{L}}}]}D_{{{\widetilde{\Omega }}_{{ij}}}}\phi \nonumber \\{} & {} + \widetilde{V}\Big (\tfrac{1}{r^*}\Big )D_{{{\widetilde{\Omega }}_{{ij}}}}\phi -\tfrac{C_{\widetilde{V}}{{\widetilde{L}}}(r^*\phi )}{r^*}\Big |\lesssim \tfrac{{\tau _{-}^{}}}{r^{*2}} \end{aligned}$$

Therefore, we have the pointwise bounds

6.29 $$\begin{aligned}&\sum _{\begin{array}{c} |I|, |J| \le 1 \\ \widetilde{V}\in \{{\partial _{r^*}}, {{\widetilde{S}}}, {{\widetilde{\Omega }}_{{0r^*}}}, {{\widetilde{\Omega }}_{{ij}}}\}\widetilde{U} \in \{{{\widetilde{\Omega }}_{{ij}}}\} \end{array}}\Big |{\tau _{+}^{s}}D_{\widetilde{V}}^ID_{\widetilde{U}}^J\Big (\tfrac{D_{{{\widetilde{L}}}}(r^*\phi )}{r^*}\Big )\Big |\nonumber \\&\quad \lesssim \sum _{|I| \le 2}\left| {\tau _{+}^{s}}\tfrac{D_{{\widetilde{L}}}(r^*D_{{\widetilde{X}}}^I\phi )}{r^*}\right| \nonumber \\&\quad + \sum _{|I| \le 1}{\tau _{+}^{s}}{\tau _{0}^{}}\left| \tfrac{D_{{\widetilde{X}}}^I\phi }{r^*}\right| \nonumber \\&\quad +\sum _{|I| + |J|\le 1} {\tau _{+}^{s+1}}|\alpha [{\mathcal {L}}_{{\widetilde{X}}}^I{\textbf{F}}]||D_{{\widetilde{Y}}}^J\phi |\nonumber \\&\quad + \sum _{|I| + |J|\le 1 } {\tau _{+}^{s}}{\tau _{-}^{}}|\rho [{\mathcal {L}}_{{\widetilde{X}}}^I{\textbf{F}}]||D_{{\widetilde{Y}}}^J\phi | . \end{aligned}$$

It suffices to show that all terms appearing on the right-hand side of (6.29) can be bounded by the energy when inserted into (6.21). The first two terms on the right-hand side appear almost immediately in $S_2[\phi ]$, using the estimate ${\tau _{0}^{}}\le {\tau _{0}^{1/2+{\overline{\mu }}}}$ for the second term.

In order to bound ${\tau _{+}^{}}|\alpha ({\mathcal {L}}_{{\widetilde{X}}}{\textbf{F}})||D_{{\widetilde{Y}}}\phi |$, the bounds (2.50) give us

6.30a $$\begin{aligned} \left\Vert {\tau _{+}^{s+1}}|\alpha ({\mathcal {L}}_{X}{{\textbf{F}}^1})||D_Y\phi |(w')^{1/2}\right\Vert _2&\lesssim |{\tau _{+}^{}}D_Y\phi |\left\Vert {\tau _{+}^{s}}\alpha [{\mathcal {L}}_{X}{\textbf{F}}^1](w')^{1/2}\right\Vert _2\nonumber \\&\lesssim {\mathcal {E}}_3[\phi ](T)^{1/2}{\mathcal {E}}_1[{\textbf{F}}^1](T)^{1/2}, \end{aligned}$$

6.30b $$\begin{aligned} \left\Vert {\tau _{+}^{s+1}}|\alpha [{\mathcal {L}}_{{\widetilde{X}}}{{\textbf{F}}^0}]||D_{{\widetilde{Y}}}\phi |(w')^{1/2}\right\Vert _2&\lesssim |q|\left\Vert {\tau _{0}^{}}{\tau _{+}^{s}}\Big |\tfrac{D_{{\widetilde{Y}}}\phi }{r^*}\Big |(w')^{1/2}\right\Vert _2 \lesssim |q|{\mathcal {E}}_2[\phi ](T)^{1/2} \end{aligned}$$

Similarly,

6.31a $$\begin{aligned} \left\Vert {\tau _{+}^{s}}{\tau _{-}^{}}|\rho ({\mathcal {L}}_{{\widetilde{X}}}{\textbf{F}}^1)||D_{{\widetilde{Y}}}\phi |(w')^{1/2}\right\Vert _2&\lesssim |{\tau _{+}^{}}D_{{\widetilde{Y}}}\phi |\left\Vert {\tau _{+}^{s}}{\tau _{0}^{}}\rho ({\mathcal {L}}_{{\widetilde{X}}}{\textbf{F}}^1)(w')^{1/2}\right\Vert _2\nonumber \\&\lesssim {\mathcal {E}}_3[\phi ](T)^{1/2}{\mathcal {E}}_1[{\textbf{F}}^1](T)^{1/2}, \end{aligned}$$

6.31b $$\begin{aligned} \left\Vert {\tau _{+}^{s}}{\tau _{-}^{}}|\rho ({\mathcal {L}}_{{\widetilde{X}}}{\textbf{F}}^0)||D_{{\widetilde{Y}}}\phi |(w')^{1/2}\right\Vert _2&\lesssim |q|\left\Vert {\tau _{0}^{}}{\tau _{+}^{s}}\left| \tfrac{D_{{\widetilde{Y}}}\phi }{r^*}\right| (w')^{1/2}\right\Vert _2\lesssim |q|{\mathcal {E}}_2[\phi ](T)^{1/2}. \end{aligned}$$

Therefore,

6.32 $$\begin{aligned}{} & {} \left\Vert {\tau _{+}^{s+1}}{\tau _{-}^{1/2}} \tfrac{D_{{{\widetilde{L}}}}(r^*\phi )}{r^*}(w')^{1/2}\right\Vert _{L^2(t)L^\infty (x)}\nonumber \\{} & {} \quad \lesssim (1+|q|+{\mathcal {E}}_1[{\textbf{F}}^1](T)^{1/2}){\mathcal {E}}_3[\phi ](T)^{1/2}. \end{aligned}$$

We consider the far interior, where derivatives satisfy the same bounds. By (10.18)

$$\begin{aligned} \left\Vert {\tau _{+}^{s+1-{{\overline{\mu }}}}}|D\phi |\right\Vert ^2_{L^2_t(L^\infty ( {\mathbb {R}}^3\cap \{r< t/2\}))} \lesssim \sum _{|I| \le 2}\left\Vert {\tau _{+}^{s-1/2-{{\overline{\mu }}}}}D_{{\widetilde{X}}}^ID\phi \right\Vert ^2_{L^2_t(L^\infty ( {\mathbb {R}}^3\cap \{r < 3t/4\}))}. \end{aligned}$$

We commute through and bound commutators as in the proof of Lemma 6.1 to get

6.33 $$\begin{aligned}{} & {} \left\Vert {\tau _{+}^{s+1-{{\overline{\mu }}}}}|D\phi |\right\Vert _{L^2_t(L^\infty ( {\mathbb {R}}^3\cap \{r < t/2\}))} \nonumber \\{} & {} \quad \lesssim \left( 1+\sum _{|I|\le 1}\left\Vert {\mathcal {L}}_{{\widetilde{X}}}{\textbf{F}}\right\Vert ^{red}_{L^\infty [w]}\right) ({\mathcal {E}}_2[\phi ](T))^{1/2}. \end{aligned}$$

We can combine these to get our main results:

### Lemma 6.4

For a suitably regular function $\phi$, we have the estimate

6.34 $$\begin{aligned}{} & {} \Big \Vert {\tau _{+}^{s+1}}{\tau _{-}^{1/2}} \left( \tfrac{D_{{\widetilde{L}}}(r^*\phi )}{r^*}\chi _{\{r^* \ge t/2\}} + {{\widetilde{L}}}(\phi )\chi _{\{r^* \le t/2\}}\right) (w')^{1/2}\Big \Vert _{L^2_t(L^\infty _x)} \nonumber \\{} & {} \quad \lesssim \left( 1+\sum _{|I|\le 1}\left\Vert {\mathcal {L}}_{{\widetilde{X}}}{\textbf{F}}\right\Vert ^{red}_{L^\infty [w]}\right) ^{1/2}({\mathcal {E}}_3[\phi ](T)). \end{aligned}$$

Finally, we turn our attention to the pure $L^\infty$ bound on the nice terms, for which we will use the following approach. Recalling Lemmas 4.5 and 4.6, we will first show the bound

6.35 $$\begin{aligned} C_0^+[\phi ](U,T) \lesssim C_0[\phi ](T) + \varepsilon _g\left( 1+\sum _{|I|\le 1}\left\Vert {\mathcal {L}}_{{\widetilde{X}}}{\textbf{F}}\right\Vert ^{red}_{L^\infty [w]}\right) ^2(E_2[\phi ](T)),\nonumber \\ \end{aligned}$$

for some uniform constant then apply (10.17), bounding the commutator terms. By the reverse triangle inequality $C_0^+[\phi ](U,T) \ge C_0[\phi ](T) - C_{err}^U[\phi ](T)$, it suffices to show

6.36 $$\begin{aligned} C^U_{err}[\phi ](T)\lesssim \varepsilon _gC_0^+[\phi ](U,T) + \varepsilon _g \left( 1+\sum _{|I|\le 1}\left\Vert {\mathcal {L}}_{{\widetilde{X}}}{\textbf{F}}\right\Vert ^{red}_{L^\infty [w]}\right) ^2(E_2[\phi ](T)).\nonumber \\ \end{aligned}$$

This follows first from the estimates

$$\begin{aligned} |(g^{\alpha \beta }-{{\widehat{m}}}^{\alpha \beta })\partial _\alpha {u^{*{}}}D_\beta \phi |&\lesssim \varepsilon _g({\tau _{+}^{-1+\delta }}|{\overline{D}}\phi | + {\tau _{0}^{\gamma }}{\tau _{+}^{-1+\delta }}|D\phi |),\\ |\widetilde{K^s}{}({u^{*{}}}) (g^{\gamma \delta } - {{\widehat{m}}}^{\gamma \delta })D_\gamma \phi \overline{D_\delta \phi }|&\lesssim \varepsilon {\tau _{+}^{-1+\delta }}{\tau _{-}^{2s-1}}({\tau _{0}^{\gamma }}|D\phi |^2 + |D\phi ||{\overline{D}}\phi |) \end{aligned}$$

Hölder’s inequality gives

6.37 $$\begin{aligned}{} & {} \int _{C_{U,T}}\Big |\tfrac{{\tau _{+}^{2s}}{{\widetilde{L}}}(r^*\phi )}{r^*} \Big | |(g^{\beta \gamma } - {{\widehat{m}}}^{\beta \gamma })D_\gamma \phi \partial _\beta ({u^{*{}}})| w\, dV_C\nonumber \\{} & {} \quad \lesssim \varepsilon _g (C_0^+)^{1/2}\left( \int _{C_{U,T}}{\tau _{+}^{2s}}|(g^{\beta \gamma } - {{\widehat{m}}}^{\beta \gamma })D_\gamma \phi \partial _\beta ({u^{*{}}})|^2 w\, dV_C\right) ^{1/2} \end{aligned}$$

Then, Lemmas 6.3 and 6.2 allow us to bound the right by

$$\begin{aligned} \left( 1+\sum _{|I|\le 1}\left\Vert {\mathcal {L}}_{{\widetilde{X}}}{\textbf{F}}\right\Vert ^{red}_{L^\infty [w]}\right) (E_2[\phi ](T))^{1/2}. \end{aligned}$$

We start with the conical estimate

6.38 $$\begin{aligned}{} & {} \left\Vert {\tau _{+}^{3/2+s}}\chi \tfrac{D_{{{\widetilde{L}}}}(r^*\phi )}{r^*}w^{1/2}\right\Vert ^2_{L^\infty (C_{U,T})}\nonumber \\{} & {} \quad \lesssim \sum _{\begin{array}{c} |I|, |J| \le 1 \\ \widetilde{V}\in \{{\partial _{r^*}}, {{\widetilde{S}}}, {{\widetilde{\Omega }}_{{0r^*}}}, {{\widetilde{\Omega }}_{{ij}}}\}\widetilde{U} \in \{{{\widetilde{\Omega }}_{{ij}}}\} \end{array}} \left\Vert {\tau _{+}^{s}}D_{\widetilde{V}}^ID_{\widetilde{U}}^J\left( \chi \tfrac{D_{{{\widetilde{L}}}}(r^*\phi )}{r^*}\right) w^{1/2}\right\Vert ^2_{L^2(C_{U,T})}.\nonumber \\ \end{aligned}$$

We can move $\chi$ outside the derivatives on the right-hand side with no issue, as when derivatives fall on it we get a uniformly bounded quantity. We again use the bound (6.29) and bound the right-hand side term-by-term. The first term on the right appears directly in the energy. For the second term, Lemma 6.3 gives

6.39 $$\begin{aligned} {\tau _{+}^{s}}{\tau _{0}^{}}\chi \left| \tfrac{D_X^I\phi }{r^*}\right| w^{1/2} \lesssim {\tau _{+}^{s-3}}{\tau _{-}^{3/2-s}}{\mathcal {E}}_3[\phi ]^{1/2}. \end{aligned}$$

and directly integrate the corresponding $L^2$ integral. The arguments for the third and fourth terms follow nearly exactly those for the bounds (6.30), (6.31), with the caveat that we use Lemma 6.3 to bound the $L^2$ norms containing $\phi$ by ${{\mathcal {E}}}_3[\phi ]$. Therefore, we can say

6.40 $$\begin{aligned} \left| {\tau _{+}^{3/2+s}}\left| \chi \tfrac{D_{{{\widetilde{L}}}}(r^*\phi )}{r^*}\right| w^{1/2}\right|\lesssim & {} \left( 1+\sum _{|I|\le 1}\left\Vert {\mathcal {L}}_{{\widetilde{X}}}{\textbf{F}}\right\Vert ^{red}_{L^\infty [w]}\right) ({\mathcal {E}}_3[\phi ](T) + C_0^+[\phi ](T))\nonumber \\\lesssim & {} \left( 1+\sum _{|I|\le 1}\left\Vert {\mathcal {L}}_{{\widetilde{X}}}{\textbf{F}}\right\Vert ^{red}_{L^\infty [w]}\right) ^2{\mathcal {E}}_4[\phi (T) \end{aligned}$$

### Proposition 6.5

For any function $\phi$ with sufficient decay, we have the bound

6.41 $$\begin{aligned} \left| {\tau _{+}^{3/2+s}}\left| \chi \tfrac{D_{{{\widetilde{L}}}}(r^*\phi )}{r^*}\right| w^{1/2}\right| \lesssim \left( 1+\sum _{|I|\le 1}\left\Vert {\mathcal {L}}_{{\widetilde{X}}}{\textbf{F}}\right\Vert ^{red}_{L^\infty [w]}\right) ^2{\mathcal {E}}_4^{1/2}[\phi ]. \end{aligned}$$

Combining this with Theorem (5.17) and the bounds (2.50), we have

### Theorem 6.6

Given a function $\phi$, and a two-form ${\textbf{F}}$ as in (1.14), we have the estimates

6.42a $$\begin{aligned}&\left( {\tau _{+}^{}}{\tau _{-}^{s-1/2}}|\phi | + {\tau _{+}^{}}{\tau _{-}^{1/2+s}}|D_{{\underline{{\widetilde{L}}}}}\phi | + {\tau _{+}^{s+1}}{\tau _{-}^{1/2}}(|{\overline{D}}\phi |)\right) w^{1/2} \nonumber \\&\quad \lesssim {\mathcal {E}}_2^{1/2}[\phi ](T)\Big (1+|q[{\textbf{F}}]| + {{\mathcal {E}}}_4^{1/2}[{\textbf{F}}^1]\Big ), \end{aligned}$$

6.42b $$\begin{aligned}&\Big \Vert {\tau _{+}^{s+1}}{\tau _{-}^{1/2}} \Big (\chi \tfrac{D_{{\widetilde{L}}}(r^*\phi )}{r^*} + (1-\chi )D_{{{\widetilde{L}}}}(\phi )\Big )(w')^{1/2}\Big \Vert _{L^2_t(L^\infty _x)}\nonumber \\&\quad \lesssim {\mathcal {E}}^{1/2}_3[\phi ](T)\Big (1+|q[{\textbf{F}}]| + {{\mathcal {E}}}_4^{1/2}[{\textbf{F}}^1]\Big ), \end{aligned}$$

6.42c $$\begin{aligned}&{\tau _{+}^{s+3/2}}\left| \chi \tfrac{D_{{{\widetilde{L}}}}(r^*\phi )}{r^*}\right| w^{1/2} \lesssim {\mathcal {E}}^{1/2}_4[\phi ](T)\Big (1+|q[{\textbf{F}}]| + {{\mathcal {E}}}_4^{1/2}[{\textbf{F}}^1]\Big )^2, \end{aligned}$$

6.42d $$\begin{aligned}&{\tau _{+}^{s}}{\tau _{-}^{1/2}}|\phi |w^{1/2}\lesssim {\mathcal {E}}^{1/2}_1[\phi ](T) \end{aligned}$$

### Remark 6.7

This is for a generic function $\phi$, so in particular we may find bounds on higher derivatives of $\varvec{\phi }$ without requiring higher derivatives of ${\textbf{F}}$. One may in particular improve the bounds on  along the light cone by writing the null frame as a linear combination of the rotation fields $\Omega _{ij}$, at the cost of requiring more derivatives.

## The Bootstrap Estimate

We are now able to begin the proof of Theorem (1.1) in earnest. We recall the energy norms

$$\begin{aligned} {\mathcal {E}}_0[{\textbf{G}}](T)&= E_0[{\textbf{G}}](T) + S_0[{\textbf{G}}](T)+C_0[{\textbf{G}}](T), \\ {\mathcal {E}}_0[\phi ](T)&= E_0[\phi ](T) + S_0[\phi ](T)+C_0[\phi ](T), \\ {\mathcal {E}}_k(T) ={\mathcal {E}}_k[{\textbf{F}}, \varvec{\phi }](T)&= \sum _{\begin{array}{c} |I| \le k,{\widetilde{X}}\in {{\mathbb {L}}} \end{array}} {\mathcal {E}}_0[{\mathcal {L}}_{{\widetilde{X}}}^I{\textbf{F}}^1](T) + {\mathcal {E}}_0[D_{{\widetilde{X}}}^I\varvec{\phi }] + |q[{\textbf{F}}]|^2. \end{aligned}$$

We fix a constant $k \ge 11$ and take the bootstrap assumption

7.1 $$\begin{aligned} {\mathcal {E}}_k(T) \le \varepsilon _b^2. \end{aligned}$$

For now, we assume $\varepsilon _b \le 1$, and $\varepsilon _1$ is as bounded in the previous sections. Then, Theorem 3.1 implies

7.2 $$\begin{aligned} {\mathcal {E}}_k[{\textbf{F}}^1](T) \lesssim {\mathcal {E}}_k[{\textbf{F}}^1](0) + \sum _{|I| \le k}\left\Vert {\textbf{J}}[{\mathcal {L}}_{{\widetilde{X}}}^I {\textbf{F}}^1]\right\Vert ^2_{L^2[w]}. \end{aligned}$$

Theorem 5.6 and (2.50) imply that for $|I_1| \le k-2, |I_2|\le k-4$, and for all *I*,

7.3a $$\begin{aligned} {\tau _{+}^{}}{\tau _{-}^{s+1/2}}|{\underline{\alpha }}[{\mathcal {L}}_{{\widetilde{X}}}^{I_1}{\textbf{F}}^1]|w^{1/2} + {\tau _{+}^{s+1}}{\tau _{-}^{1/2}}(|\rho [{\mathcal {L}}_{{\widetilde{X}}}^{I_1}{\textbf{F}}^1]|+|\sigma [{\mathcal {L}}_{{\widetilde{X}}}^{I_1}{\textbf{F}}^1]|)w^{1/2}&\lesssim {\mathcal {E}}^{1/2}_{k}(T) \end{aligned}$$

7.3b $$\begin{aligned} \Vert {\tau _{+}^{s+1}}{\tau _{-}^{1/2}}\alpha [{\mathcal {L}}_{{\widetilde{X}}}^{I_1}{\textbf{F}}^1](w')^{1/2}\Vert _{L^2(L^\infty )} + |{\tau _{+}^{3/2+s}}\alpha [{\mathcal {L}}_{{\widetilde{X}}}^{I_2}{\textbf{F}}^1] w^{1/2}|&\lesssim {\mathcal {E}}^{1/2}_{k}(T), \end{aligned}$$

7.3c $$\begin{aligned} {\tau _{+}^{3}}{\tau _{-}^{-1}}|\alpha [{\mathcal {L}}_{{\widetilde{X}}}^I{\textbf{F}}^0]| + {\tau _{+}^{2}}(|\rho [{\mathcal {L}}_{{\widetilde{X}}}^I{\textbf{F}}^0]| +|\sigma [{\mathcal {L}}_{{\widetilde{X}}}^I{\textbf{F}}^0]| +|{\underline{\alpha }}[{\mathcal {L}}_{{\widetilde{X}}}^I{\textbf{F}}^0]|)&\lesssim {\mathcal {E}}^{1/2}_{k}(T). \end{aligned}$$

It follows that

7.4 $$\begin{aligned} \left\Vert {{\textbf{F}}^1} \right\Vert ^{red}_{L^{\infty }[w]} \lesssim {\mathcal {E}}^{1/2}_{4}(T) \end{aligned}$$

so Theorem 4.1 implies

7.5 $$\begin{aligned} {\mathcal {E}}_k[\varvec{\phi }](T) \lesssim {\mathcal {E}}_k[\varvec{\phi }](0) + {\mathcal {E}}_k^{3/2}(T) + \sum _{|I|\le k}\left\Vert {\tau _{+}^{s}}{\tau _{-}^{1/2}}\Box _g^{{\mathbb {C}}}(D_{{\widetilde{X}}}^I\varvec{\phi })w_{{\overline{\mu }}}^{1/2}\right\Vert ^2_{L^2[w]}. \end{aligned}$$

For sufficiently small $\varepsilon _b$, we may say

7.6 $$\begin{aligned} C{\mathcal {E}}_k^{3/2}(T) \le \tfrac{1}{2} {\mathcal {E}}_k(T). \end{aligned}$$

Similarly, for $|I_1| \le k-2$, $|I_2|\le k-4$ and $|I_3|\le k-1$, Theorem (6.6) implies

7.7a $$\begin{aligned}&\left( {\tau _{+}^{}}{\tau _{-}^{s-1/2}}|D_{{\widetilde{X}}}^{I_1}\varvec{\phi }| + {\tau _{+}^{}}{\tau _{-}^{1/2+s}}|D_{{\underline{{\widetilde{L}}}}}D_{{\widetilde{X}}}^{I_1}\varvec{\phi }| + {\tau _{+}^{s+1}}{\tau _{-}^{1/2}}(|{\overline{D}}D_{{\widetilde{X}}}^{I_1}\varvec{\phi }|)\right) w^{1/2} \lesssim {\mathcal {E}}_k^{1/2}(T), \end{aligned}$$

7.7b $$\begin{aligned}&\Big \Vert {\tau _{+}^{s+1}}{\tau _{-}^{1/2}} \Big (\chi \tfrac{D_{{\widetilde{L}}}(r^*D_{{\widetilde{X}}}^{I_1}\varvec{\phi })}{r^*} + (1-\chi )D_{{{\widetilde{L}}}}(D_{{\widetilde{X}}}^{I_1}\varvec{\phi })\Big )(w')^{1/2}\Big \Vert _{L^2_t(L^\infty _x)} \lesssim {\mathcal {E}}_k^{1/2}(T), \end{aligned}$$

7.7c $$\begin{aligned}&{\tau _{+}^{s+3/2}}\left| \chi \tfrac{D_{{{\widetilde{L}}}}(r^*D_{{\widetilde{X}}}^{I_2}\varvec{\phi })}{r^*}\right| w^{1/2} \lesssim {\mathcal {E}}_k^{1/2}(T), \end{aligned}$$

7.7d $$\begin{aligned}&{\tau _{+}^{s}}{\tau _{-}^{1/2}}|D_{{\widetilde{X}}}^{I_3}\varvec{\phi }|w^{1/2}\lesssim {\mathcal {E}}_k^{1/2}(T) \end{aligned}$$

Consequently, adding (7.2) and (7.5) gives

### Theorem 7.1

Given $(\varvec{\phi },{\textbf{F}})$ which solves the MKG system (1.14) for time [0, *T*] and satisfies the bootstrap assumption (7.1), the following estimate holds for sufficiently small $\varepsilon _g, \varepsilon _b$:

7.8 $$\begin{aligned} {\mathcal {E}}_k(T) \lesssim {\mathcal {E}}_k(0) + \sum _{|I| \le k}\left\Vert {\textbf{J}}[{\mathcal {L}}_{{\widetilde{X}}}^I{\textbf{F}}^1]\right\Vert _{L^2[w]}^2 + \sum _{|I| \le k}\left\Vert (\Box _g^{{\mathbb {C}}}D_{{\widetilde{X}}}^I\varvec{\phi }){\tau _{+}^{s}}{\tau _{-}^{1/2}}w_{{\overline{\mu }}}^{1/2}\right\Vert _2^2.\nonumber \\ \end{aligned}$$

We restate Theorem 1.1 in a form which will be useful for the full system.

### Theorem 7.2

Take constants $s, s_0, \gamma , \mu , \delta , {{\overline{\mu }}}$ such that $\frac{1}{2}< s< 1< s_0 < 3/2$, $\mu < 1/2$, $\gamma >1/2$, $2\,s < 1+\gamma$, and $0< 4\delta , 4{\overline{\mu }} < \min (1-2\mu , s-\frac{1}{2}, 1-s, \gamma -\frac{1}{2}, 1-\gamma , 1+\gamma -2\,s)$. Additionally, let $k_0$ be an integer with $k_0 \ge 11$.

There exist constants $\varepsilon _0$, $\varepsilon _1> 0$ such that if the metric, in the decomposition (1.22), satisfies (1.23) and (1.25) for $\varepsilon _g < \varepsilon _1$, and

$$\begin{aligned} {\mathcal {E}}_k(0)\le \varepsilon ^2. \end{aligned}$$

for $\varepsilon < \varepsilon _0$, then there exists a constant *C* independent of *T* such that

7.9 $$\begin{aligned} {\mathcal {E}}_k(t)\le C\varepsilon ^2. \end{aligned}$$

Additionally, the bounds (7.3), (7.7) hold with right-hand sides replaced by $C\varepsilon$.

To prove Theorem 7.2, it suffices to prove the bound

7.10 $$\begin{aligned}{} & {} \sum _{|I| \le k}\left\Vert {\textbf{J}}[{\mathcal {L}}_{{\widetilde{X}}}^I{\textbf{F}}^1]\right\Vert _{L^2[w]}^2 + \sum _{|I| \le k}\left\Vert (\Box _g^{{\mathbb {C}}}D_{{\widetilde{X}}}^I\varvec{\phi }){\tau _{+}^{s}}{\tau _{-}^{1/2}}w_{{\overline{\mu }}}^{1/2}\right\Vert _2^2\nonumber \\{} & {} \quad \lesssim |q|^2+{\mathcal {E}}_k^2(T) + \varepsilon _g^2{\mathcal {E}}_k(T). \end{aligned}$$

We will put this off until the following sections, where it follows from Lemmas 8.1 and 9.1. To conclude the proof of Theorem 1.1, we combine Theorem 7.2 with Proposition 7.3.

### The Initial Data Bounds

Before we conclude this, we must show that the initial conditions of Theorem 7.1 are compatible with those for Theorem 1.1. To be precise, we need to show the following estimate:

#### Proposition 7.3

Under the constraints of Theorem (1.1), we have the bound

7.11 $$\begin{aligned} {\mathcal {E}}_k(0) \lesssim \left\Vert {\textbf{E}}_{0df}\right\Vert ^2_{H^{k, s_0}({\mathbb {R}}^3)} + \left\Vert {\textbf{B}}_0\right\Vert ^2_{H^{k, s_0}({\mathbb {R}}^3)} + \left\Vert D\varvec{\phi }_0\right\Vert ^2_{H^{k, s_0}({\mathbb {R}}^3)} + \Vert \,\dot{\!\varvec{\phi }_0}\Vert ^2_{H^{k, s_0}({\mathbb {R}}^3)}.\nonumber \\ \end{aligned}$$

This largely follows the proof in the Minkowski case, with some adaptations made to account for the metric. From the definition (2.45), Hölder’s inequality and the condition $s>\tfrac{1}{2}$,

7.12 $$\begin{aligned} |q|\le \left\Vert (1+r^*)^{s}{\textbf{J}}^0\right\Vert _{L^{6/5}_x}\Vert (1+r^*)^{-s}\Vert _{L^6_x} \le C\left\Vert (1+r^*)^{s}{\textbf{J}}^0\right\Vert _{L^{6/5}_x} \end{aligned}$$

We now restate a technical elliptic estimate, Lemma 10.1 (and its generalization (3.53))in [18]:

#### Lemma 7.4

Let $\psi$ be a smooth test function on ${\mathbb {R}}^3$, and define

7.13 $$\begin{aligned} q[\phi ] = \int _{{\mathbb {R}}^3}\psi \, dx. \end{aligned}$$

Then, for $$\tfrac{1}{2}< s_0 < \tfrac{3}{2}$$,

7.14 $$\begin{aligned} \int _{{\mathbb {R}}^3}r^{2s_0}\Big |\nabla \Big (\Delta ^{-1}\psi + \tfrac{q[\phi ]}{4\pi r}\Big )\Big |^2 \, dx\lesssim \Vert r^{s_0}\psi \Vert ^2_{L^{6/5}_x} \end{aligned}$$

Additionally,

7.15 $$\begin{aligned} \sum _{|I| \le k}\int _{{\mathbb {R}}^3}r^{2s_0+2|I|}\left| \nabla \nabla _x^I\left( \Delta ^{-1}\psi + \tfrac{q[\psi ]}{4\pi r}\right) \right| ^2\,dx \lesssim \sum _{|I| \le k}\left\Vert r^{s_0+|I|}\nabla _x^I \psi \right\Vert ^2_{L^{6/5}_x}.\nonumber \\ \end{aligned}$$

Applying these estimates to $\psi = -\sqrt{|\widetilde{g}|}{\textbf{J}}^0$ in the $\widetilde{x}$ coordinates gives

7.16a $$\begin{aligned} \left\Vert r^{*s_0} \left( {\textbf{E}}_{cf}^i[{\textbf{F}}] - {\textbf{F}}^0_{0i}\right) \right\Vert _{L^2_x}&\lesssim \left\Vert r^{*s_0} (\sqrt{|\widetilde{g}|}{\textbf{J}}^0) \right\Vert _{L^{6/5}_x}, \end{aligned}$$

7.16b $$\begin{aligned} \left\Vert r^{*s_0+|I|} \left( {\textbf{E}}_{cf}^i[\nabla _{\widetilde{x}}^I{\textbf{F}}] - \nabla _{\widetilde{x}}^I{\textbf{F}}^0_{0i}\right) \right\Vert _{L^2_x}&\lesssim \left\Vert r^{*s_0+|I|}\nabla _{\widetilde{x}}^I (\sqrt{|\widetilde{g}|}{\textbf{J}}^0) \right\Vert _{L^{6/5}_x}. \end{aligned}$$

Consequently,

7.17 $$\begin{aligned} {\int _{\Sigma _{0}}}(1+r^*)^{2s_0}|{\textbf{E}}[{\textbf{F}}^1]|^2\, dx\lesssim & {} {\int _{\Sigma _{0}}}(1+r^*)^{2s_0}|{\textbf{E}}_{df}|^2\, dx\nonumber \\{} & {} + \left\Vert (1+r^*)^{s_0}{\textbf{J}}^0\right\Vert ^2_{L^{6/5}(x)}, \end{aligned}$$

We bound ${\textbf{J}}^0$ with the following general estimate on ${\mathbb {R}}^3$ for suitably regular $f_1, f_2$:

7.18 $$\begin{aligned} \Vert \langle r^*\rangle ^{s_0}f_1 f_2\Vert _{L^{6/5}_x }\le & {} \Vert \langle r^*\rangle ^{s_0}f_1\Vert _{L^2_x }\Vert f_2\Vert _{L^3_x }\le \Vert \langle r^*\rangle ^{s_0}f_1\Vert _{L^2_x }\Vert \partial f_2\Vert _{L^{3/2}_x } \nonumber \\\le & {} \Vert \langle r^*\rangle ^{s_0}f_1\Vert _{L^2_x }\Vert \langle r^*\rangle ^{s_0}\partial f_2\Vert _{L^{2}_x }\Vert \langle r^*\rangle ^{-s_0}\Vert _{L^{6}_x } \end{aligned}$$

In order to apply this, we first expand with (2.42):

7.19 $$\begin{aligned} \big |{\mathcal {L}}_{X}^I{\widetilde{{\textbf{J}}}}^0\big | \lesssim \sum _{|I_1|+|I_2|+|I_3|\le |I|}\big |{{^{({{\widetilde{X}}^{I_1}})}{\Pi }}}\big |\big |{{\mathcal {L}}_{{\widetilde{X}}}^{{\mathbb {C}}{I_2}}}\varvec{\phi }\big |\big |{{\mathcal {L}}_{{\widetilde{X}}}^{{\mathbb {C}}{I_3}}}\widetilde{D}\varvec{\phi }\big | \end{aligned}$$

If $|I_1| \le k-6$, we bound $|{{^{({{\widetilde{X}}^{I_1}})}{\Pi }}}|$ uniformly by a constant and apply (7.18) with $f_1 = |{{\mathcal {L}}_{{\widetilde{X}}}^{{\mathbb {C}}{I_3}}}\widetilde{D}\varvec{\phi }|, f_2 = |{{\mathcal {L}}_{{\widetilde{X}}}^{{\mathbb {C}}{I_2}}}\varvec{\phi }|$, followed by (10.1). Otherwise, set $f_1=|{{^{({{\widetilde{X}}^{I_1}})}{\Pi }}}||{{\mathcal {L}}_{{\widetilde{X}}}^{{\mathbb {C}}{I_3}}}\widetilde{D}\varvec{\phi }|$, $f_2 =|{{\mathcal {L}}_{{\widetilde{X}}}^{{\mathbb {C}}{I_2}}}\varvec{\phi }|$ and use Lemma 6.1 to bound $|{{\mathcal {L}}_{{\widetilde{X}}}^{{\mathbb {C}}{I_3}}}\widetilde{D}\varvec{\phi }|$. This gives the result

7.20 $$\begin{aligned} \sum _{|I|\le k}\left\Vert r^{*s_0+|I|}\nabla _{\widetilde{x}}^I (\sqrt{|\widetilde{g}|}{\textbf{J}}^0) \right\Vert _{L^{6/5}_x}\lesssim (1+\varepsilon _g){\mathcal {E}}_k(0). \end{aligned}$$

Next we seek to bound

$$\begin{aligned} \Vert \langle r^*\rangle ^{s_0+|I|} \nabla _{\widetilde{x}}^I{\textbf{F}}^1\Vert _{L^2_x} \end{aligned}$$

For this we use the identities

$$\begin{aligned} d{\textbf{F}}= 0, \quad \nabla ^\beta {\textbf{F}}_{\alpha \beta } = {\textbf{J}}_\alpha . \end{aligned}$$

which will allow us to bound terms where the derivative falls on the magnetic and electric field components, respectively. For an initially split metric *g*, the harmonic coordinate condition implies

7.21 $$\begin{aligned} {\widetilde{\partial }}_0{\textbf{G}}_{i0}= {\widetilde{g}}_{00}({\textbf{J}}[{\textbf{G}}]_i- \widetilde{g}^{jk}{\widetilde{\partial }}_j{\textbf{G}}_{ik}-{\widetilde{g}}^{ab} {\widetilde{\Gamma }}^c_{a i}{\textbf{G}}_{cb}). \end{aligned}$$

We now show the initial bound for ${\textbf{F}}^1$. Given ${\mathcal {L}}_{\widetilde{X}}^I{\textbf{F}}^1$, we decompose, commute time derivatives to the right, and use the $L^\infty$ bounds

$$\begin{aligned} \sum _{1 \le |a|\le k-6}(1+r^*)^a{\widetilde{\partial }}^a \widetilde{g}^{ij}\lesssim 1, \quad \sum _{1 \le |a|\le k}(1+r^*)^a{\widetilde{\partial }}^a {\widetilde{g}}_{00}\lesssim 1 \end{aligned}$$

whenever applicable to get, for multiindices $a_i$,

7.22 $$\begin{aligned} \sum _{|I|\le k }|{\mathcal {L}}_{\widetilde{X}}^I{\textbf{F}}^1|&\lesssim \sum _{|a_1|+a_t\le k }(1+r^{*})^{s_0+|a_1|+a_t}|{\widetilde{\partial }}_{\widetilde{x}}^{a_1}{\widetilde{\partial }}_0^{a_t} {\textbf{F}}^1|\nonumber \\&\lesssim \sum _{|a_1|+|a_2|\lesssim k}(1+r^{*})^{s_0+|a_1|+|a_2|}|{\widetilde{\partial }}^{a_1}\widetilde{g}||{\widetilde{\partial }}_{\widetilde{x}}^{a_2}{\textbf{F}}^1|\nonumber \\&\quad + \sum _{|a_1|+|a_2|\lesssim k-1}(1+r^{*})^{s_0+|a_1|+|a_2|+1}|{\widetilde{\partial }}^{a_1}\widetilde{g}||{\widetilde{\partial }}^{a_2}{\textbf{J}}[{\textbf{F}}^1]|. \end{aligned}$$

In order to bound the first term on the right we take standard $L^2-L^\infty$ bounds, and to bound the second term we decompose ${\textbf{J}}[{\textbf{F}}^1] = {\textbf{J}}[{\textbf{F}}] - {\textbf{J}}[{\textbf{F}}^0]$, and note that ${\textbf{J}}^0$ rapidly decays away from the origin (cf. Sect. 8.2.2), so for small $\varepsilon _g$ we may write

7.23 $$\begin{aligned} {\sum _{\le k}}\Vert \langle r^*\rangle ^{s_0} {\mathcal {L}}_{\widetilde{X}}^I{\textbf{F}}^1(0, \cdot )\Vert _{L^2_x}\lesssim & {} {\sum _{\le k}}\Vert \langle r^*\rangle ^{s_0+|I|}{\widetilde{\partial }}_{\widetilde{x}}^I{\textbf{F}}^1(0, \cdot )\Vert _{L^2_x} \nonumber \\{} & {} + \sum _{|I| \le k-1}\left\Vert |\langle r^*\rangle ^{s_0+|I|+1}{\widetilde{\partial }}^I{\textbf{J}}(0, \cdot )\right\Vert _{L^2_x}. \end{aligned}$$

Next, we look at

$$\begin{aligned} \sum _{|I| \le k-1}\left\Vert |\langle r^*\rangle ^{s_0+|I|+1}{\widetilde{\partial }}^I{\textbf{J}}(0, \cdot )\right\Vert _{L^2_x} \end{aligned}$$

In order to do this, we write ${\textbf{J}}$ as ${\mathfrak {I}}(\varvec{\phi }\overline{\widetilde{D}\varvec{\phi }})$. It suffices to bound

$$\begin{aligned} \sum _{|a_1|+|a_2|\le k-1}\left\Vert |(1+r^*)^{s_0+|a_1|+|a_2|+1}|\widetilde{D}^{a_1}\varvec{\phi }||\widetilde{D}^{a_2}\widetilde{D}\varvec{\phi }|\right\Vert _2^2. \end{aligned}$$

In order to take care of this, we first take our time-slice Sobolev estimate

7.24 $$\begin{aligned} |\langle r^*\rangle ^{1+|a|}\widetilde{D}^a\varvec{\phi }| \lesssim \sum _{|a_1|\le 2}\langle r^*\rangle ^{s_0 + |a|+|a_1|-1}|\widetilde{D}_{\widetilde{x}}^{a_1}\widetilde{D}^a\varvec{\phi }|. \end{aligned}$$

which follows from the estimate $s_0> 1/2$. We finally bound

7.25 $$\begin{aligned} \sum _{|a|\le k}\Vert \langle r^*\rangle ^{s_0+|a|}\widetilde{D}\widetilde{D}^a\varvec{\phi }\Vert _{L^2_x}. \end{aligned}$$

To bound this, we commute time derivatives to the right and recall

7.26 $$\begin{aligned} D_t^2\varvec{\phi }=-\widetilde{g}_{00}\widetilde{g}^{ij}\widetilde{D}_i\widetilde{D}_j\varvec{\phi }. \end{aligned}$$

Iterating this and applying $L^\infty$ bounds whenever possible (and noting that we may always apply $L^\infty$ bounds on *g* when they appear, as metric components are paired with two spatial derivatives of $\varvec{\phi }$) gives

7.27 $$\begin{aligned} \sum _{|a|\le k}\Vert \langle r^*\rangle ^{s_0+|a|}{\widetilde{\partial }}{\widetilde{\partial }}^a\varvec{\phi }\Vert _{L^2_x}\lesssim & {} \sum _{|a|\le k}\Vert \langle r^*\rangle ^{s_0+|a|}|\widetilde{D}\widetilde{D}_{\widetilde{x}}^a\varvec{\phi }|\Vert _{L^2_x} \nonumber \\{} & {} + \sum _{|a|\le k-1}\Vert \langle r^*\rangle ^{s_0+|a|+1}|\widetilde{D}\widetilde{D}_{\widetilde{x}}^a\widetilde{D}_0\varvec{\phi }|\Vert _{L^2_x} \end{aligned}$$

We may combine everything to get the initial decay bounds

$$\begin{aligned} {\mathcal {E}}_k(0)^{1/2} \lesssim \left\Vert {\textbf{E}}_{0df} \right\Vert _{H^{k_0, s_0}({\mathbb {R}}^3)} + \left\Vert {\textbf{B}}_0 \right\Vert _{H^{k_0, s_0}({\mathbb {R}}^3)}+ \left\Vert D\varvec{\phi }_0 \right\Vert _{H^{k_0, s_0}({\mathbb {R}}^3)} + \left\Vert \,\,\dot{\!\!\varvec{\phi }}_0 \right\Vert _{H^{k_0, s_0}({\mathbb {R}}^3)} \end{aligned}$$

as long as the right-hand side is sufficiently small.

## Commutator Estimates for **F**

Before proving Lemma 8.1, we will prove some $L^2$ and $L^\infty$ estimates which will prove useful.

### Pointwise and Energy bounds

By Theorem 6.6,

$$\begin{aligned} \sum _{|I_1|\le k-4}|D_{{\widetilde{X}}}^{I_1}\varvec{\phi }| \lesssim {{\mathcal {E}}}_k(T)^{1/2}{\tau _{+}^{-1}}{\tau _{-}^{1/2-s}}w^{-1/2}. \end{aligned}$$

Combining this with (6.4) gives

8.1a $$\begin{aligned} \sum _{|I_1|+|I_2|\le k-4}|D_{{\widetilde{X}}}^{I_1}\varvec{\phi }||({\mathcal {L}}_{{\widetilde{X}}}^{I_2}{\textbf{F}})_{{{\widetilde{X}}}_1{{\widetilde{L}}}}|&\lesssim {{\mathcal {E}}}_k(T){\tau _{+}^{-3/2-s}}{\left\langle {(r^*-t)_-}\right\rangle }^{1/2-s}w^{-1/2} \end{aligned}$$

8.1b $$\begin{aligned} \sum _{|I_1|+|I_2|\le k-4}|D_{{\widetilde{X}}}^{I_1}\varvec{\phi }||({\mathcal {L}}_{{\widetilde{X}}}^{I_2}{\textbf{F}})_{{{\widetilde{X}}}_1{{\widetilde{S}}_{i}}}|&\lesssim {{\mathcal {E}}}_k(T){\tau _{+}^{-1-s}}{\tau _{-}^{-1/2}}{\left\langle {(r^*-t)_-}\right\rangle }^{1/2-s}w^{-1/2} \end{aligned}$$

8.1c $$\begin{aligned} \sum _{|I_1|+|I_2|\le k-4}|D_{{\widetilde{X}}}^{I_1}\varvec{\phi }||({\mathcal {L}}_{{\widetilde{X}}}^{I_2}{\textbf{F}})_{{{\widetilde{X}}}_1{\underline{{\widetilde{L}}}}}|&\lesssim {{\mathcal {E}}}_k(T){\tau _{+}^{-1}}{\tau _{-}^{-1/2-s}}{\left\langle {(r^*-t)_-}\right\rangle }^{1/2-s}w^{-1/2} \end{aligned}$$

The bound ${{\overline{\mu }}}< s-\frac{1}{2}$ implies $w_{{\overline{\mu }}}^{1/2}\le w^{1/2}{\left\langle {(r^*-t)_-}\right\rangle }^{s-1/2}$, so for $|I|\le k-4$

8.2 $$\begin{aligned}{} & {} \big ({\tau _{+}^{}}{\tau _{-}^{}}|({\mathcal {L}}_{{\widetilde{X}}}^{I}D\varvec{\phi })_{{\underline{{\widetilde{L}}}}})| +{\tau _{+}^{s+1}}{\tau _{-}^{1-s}}(|({\mathcal {L}}_{{\widetilde{X}}}^{I}D\varvec{\phi })_{{{\widetilde{L}}}}|\nonumber \\{} & {} \quad + |({\mathcal {L}}_{{\widetilde{X}}}^{I}D\varvec{\phi })_{{{\widetilde{S}}_{i}}}|)\big )\langle (t-r^*)_+\rangle ^{s-1/2} w^{1/2}\lesssim {{\mathcal {E}}}_k^{1/2}(T). \end{aligned}$$

Additionally, we have the slightly better norm

8.3 $$\begin{aligned} {\tau _{+}^{s+3/2}}{\tau _{-}^{1/2-s}}\Big |\tfrac{({\mathcal {L}}_{{\widetilde{X}}}^I D(r^*\varvec{\phi }))_{{{\widetilde{L}}}}}{r^*}\Big |\langle (t-r^*)_+\rangle ^{s-1/2} w^{1/2}\lesssim {{\mathcal {E}}}_k^{1/2}(T). \end{aligned}$$

In order to show the $L^2$ bound, we first decompose ${\textbf{F}}= {\textbf{F}}^1+{\textbf{F}}^0$. Using (6.1) on (2.50) and applying that to the $D_{{\widetilde{X}}}^I\varvec{\phi }$ term in $S_0[D_{{\widetilde{X}}}^I\varvec{\phi }]$ gives

8.4a $$\begin{aligned} \sum _{|I_1|+|I_2| +1 \le k}{\left\Vert {{\tau _{+}^{s+1}}{\tau _{0}^{1/2+{{{\overline{\mu }}}}}}{\tau _{-}^{-1}}D_{{\widetilde{X}}}^{I_1}\varvec{\phi }({\mathcal {L}}_{{\widetilde{X}}}^{I_2}{\textbf{F}}^0)_{{\widetilde{X}}{{\widetilde{L}}}}(w')^{1/2}}\right\Vert }_2&\lesssim {{\mathcal {E}}}_k(T), \end{aligned}$$

8.4b $$\begin{aligned} \sum _{|I_1|+|I_2|+1 \le k}{\left\Vert {{\tau _{+}^{s}}{\tau _{0}^{1/2+{{{\overline{\mu }}}}}}D_{{\widetilde{X}}}^{I_1}\varvec{\phi }({\mathcal {L}}_{{\widetilde{X}}}^{I_2}{\textbf{F}}^0)_{{\widetilde{X}}{{\widetilde{S}}_{i}}}(w')^{1/2}}\right\Vert }_2&\lesssim {{\mathcal {E}}}_k(T), \end{aligned}$$

8.4c $$\begin{aligned} \sum _{|I_1|+|I_2| +1\le k}{\left\Vert {{\tau _{+}^{s}}{\tau _{0}^{1/2+{{{\overline{\mu }}}}}}D_{{\widetilde{X}}}^{I_1}\varvec{\phi }({\mathcal {L}}_{{\widetilde{X}}}^{I_2}{\textbf{F}}^0)_{{\widetilde{X}}{\underline{{\widetilde{L}}}}}(w')^{1/2}}\right\Vert }_2&\lesssim {{\mathcal {E}}}_k(T). \end{aligned}$$

We now prove similar bounds for ${\textbf{F}}^1$. First,

8.5a $$\begin{aligned}&\sum _{|I_1|+|I_2| +1\le k}\big \Vert {\tau _{+}^{2s}}{\tau _{-}^{1/2}}|\alpha [{\mathcal {L}}_{{\widetilde{X}}}^{I_2}{\textbf{F}}^1]||D_{{\widetilde{X}}}^{I_1}\varvec{\phi }|w^{1/2}(w')^{1/2}\big \Vert _2 \lesssim {\mathcal {E}}_k(T), \end{aligned}$$

8.5b $$\begin{aligned}&\sum _{|I_1|+|I_2| +1\le k}\big \Vert {\tau _{+}^{2s}}{\tau _{0}^{1/2+{{\overline{\mu }}}}}{\tau _{-}^{1/2}}(|\sigma [{\mathcal {L}}_{{\widetilde{X}}}^{I_2}{\textbf{F}}^1]|+|\rho [{\mathcal {L}}_{{\widetilde{X}}}^{I_2}{\textbf{F}}^1]|)|D_{{\widetilde{X}}}^{I_1}\varvec{\phi }| w^{1/2}(w')^{1/2}\big \Vert _2 \lesssim {\mathcal {E}}_k(T), \end{aligned}$$

8.5c $$\begin{aligned}&\sum _{|I_1|+|I_2| +1\le k}\big \Vert {\tau _{+}^{s}}{\tau _{0}^{1/2+{{\overline{\mu }}}}}{\tau _{-}^{s+1/2}}|{\underline{\alpha }}[{\mathcal {L}}_{{\widetilde{X}}}^{I_2}{\textbf{F}}^1]||D_{{\widetilde{X}}}^{I_1}\varvec{\phi }|w^{1/2}(w')^{1/2}\big \Vert _2 \lesssim {\mathcal {E}}_k(T), \end{aligned}$$

8.5d $$\begin{aligned}&\sum _{|I_1|+|I_2| +2\le k}\big \Vert {\tau _{+}^{}}{\tau _{0}^{1/2+{{\overline{\mu }}}}}{\tau _{-}^{2s-1/2}}|{\underline{\alpha }}[{\mathcal {L}}_{{\widetilde{X}}}^{I_2}{\textbf{F}}^1]||D_{{\widetilde{X}}}^{I_1}\varvec{\phi }|w^{1/2}(w')^{1/2}\big \Vert _2 \lesssim {\mathcal {E}}_k(T), \end{aligned}$$

To prove (8.5a), we use the $L^2(L^\infty )$ bound on $\alpha$ in Theorem 5.6 and the spatial $L^2$ norm on $\varvec{\phi }$, or the $L^\infty$ bound on $\varvec{\phi }$ and the spacetime norm on $\alpha$. To bound (8.5b), we combine the $L^\infty (L^\infty )$ bound with an $L^2(L^2)$ bound. To bound (8.5c) and (8.5d), we take $L^\infty$ estimates on $\varvec{\phi }$ terms using Lemmas 6.1 and 6.3, respectively, and bound ${\underline{\alpha }}$ in $L^2(L^2)$. Then, the decomposition (6.1) gives

8.6a $$\begin{aligned} \sum _{\begin{array}{c} |I_1|+|I_2|+1 \le k \end{array}}{\left\Vert {{\tau _{+}^{2s-1}}{\tau _{-}^{1/2}}D_{{\widetilde{X}}}^{I_1}\varvec{\phi }({\mathcal {L}}_{{\widetilde{X}}}^{I_2}{\textbf{F}}^1)_{{{\widetilde{X}}}{{\widetilde{L}}}}w^{1/2}(w')^{1/2}}\right\Vert }_2&\lesssim {{\mathcal {E}}}_k(T), \end{aligned}$$

8.6b $$\begin{aligned} \sum _{\begin{array}{c} |I_1|+|I_2|+1 \le k \end{array}}{\left\Vert {{\tau _{+}^{2s-1}}{\tau _{0}^{1/2+{{{\overline{\mu }}}}}}{\tau _{-}^{1/2}}D_{{\widetilde{X}}}^{I_1}\varvec{\phi }({\mathcal {L}}_{{\widetilde{X}}}^{I_2}{\textbf{F}}^1)_{{{\widetilde{X}}}{{\widetilde{S}}_{i}}}w^{1/2}(w')^{1/2}}\right\Vert }_2&\lesssim {{\mathcal {E}}}_k(T), \end{aligned}$$

8.6c $$\begin{aligned} \sum _{\begin{array}{c} |I_1|+|I_2|+1 \le k \end{array}}{\left\Vert {{\tau _{+}^{s-1}}{\tau _{0}^{1/2+{{{\overline{\mu }}}}}}{\tau _{-}^{s+1/2}}D_{{\widetilde{X}}}^{I_1}\varvec{\phi }({\mathcal {L}}_{{\widetilde{X}}}^{I_2}{\textbf{F}}^1)_{{{\widetilde{X}}}{\underline{{\widetilde{L}}}}}w^{1/2}(w')^{1/2}}\right\Vert }_2&\lesssim {{\mathcal {E}}}_k(T), \end{aligned}$$

8.6d $$\begin{aligned} \sum _{\begin{array}{c} |I_1|+|I_2|+2 \le k \end{array}}{\left\Vert {{\tau _{-}^{2s-1/2}}{\tau _{0}^{1/2+{{{\overline{\mu }}}}}}D_{{\widetilde{X}}}^{I_1}\varvec{\phi }({\mathcal {L}}_{{\widetilde{X}}}^{I_2}{\textbf{F}}^1)_{{{\widetilde{X}}}{\underline{{\widetilde{L}}}}}w^{1/2}(w')^{1/2}}\right\Vert }_2&\lesssim {{\mathcal {E}}}_k(T). \end{aligned}$$

Combining (8.4) and (8.6), and recalling the spacetime norms of derivatives of $\varvec{\phi }$ appearing in ${{\mathcal {E}}}_k(T)$ gives us the estimates

8.7a $$\begin{aligned} \sum _{|I|\le k}{\left\Vert {{\tau _{+}^{s}}{\tau _{0}^{1-s}}\left| \tfrac{({{\mathcal {L}}_{{\widetilde{X}}}^{{\mathbb {C}}{I}}}(r^*\varvec{\phi }))_{{{\widetilde{L}}}}}{r^*}\right| (w')^{1/2}}\right\Vert }_2&\lesssim {{\mathcal {E}}}_k(T)^{1/2}, \end{aligned}$$

8.7b $$\begin{aligned} \sum _{|I|\le k}{\left\Vert {{\tau _{+}^{s}}{\tau _{0}^{3/2-s+{{{\overline{\mu }}}}}}|({{\mathcal {L}}_{{\widetilde{X}}}^{{\mathbb {C}}{I}}} D\varvec{\phi })_{{{\widetilde{S}}_{i}}}|(w')^{1/2}}\right\Vert }_2&\lesssim {{\mathcal {E}}}_k(T)^{1/2}, \end{aligned}$$

8.7c $$\begin{aligned} \sum _{|I|\le k}{\left\Vert {{\tau _{-}^{s}}{\tau _{0}^{3/2-s+{{{\overline{\mu }}}}}}|({{\mathcal {L}}_{{\widetilde{X}}}^{{\mathbb {C}}{I}}} D\varvec{\phi })_{{\underline{{\widetilde{L}}}}}|(w')^{1/2}}\right\Vert }_2&\lesssim {{\mathcal {E}}}_k(T)^{1/2}, \end{aligned}$$

8.7d $$\begin{aligned} \sum _{|I|\le k-1}{\left\Vert {{\tau _{-}^{s}}{\tau _{0}^{1/2+{{{\overline{\mu }}}}}}|({{\mathcal {L}}_{{\widetilde{X}}}^{{\mathbb {C}}{I}}} D\varvec{\phi })_{{\underline{{\widetilde{L}}}}}|(w')^{1/2}}\right\Vert }_2&\lesssim {{\mathcal {E}}}_k(T)^{1/2}. \end{aligned}$$

### Bounding the Current Norm

We restate the current norm (3.6) with raised indices

8.8 $$\begin{aligned} \left\Vert {\textbf{J}}\right\Vert _{L^2[w]}= & {} \left\Vert {\tau _{+}^{s}}{\tau _{0}^{-1/2-{{\overline{\mu }}}}}{\tau _{-}^{1/2}} {\textbf{J}}^{{\underline{{\widetilde{L}}}}} w^{1/2}_{{\overline{\mu }}}\right\Vert _2 + \left\Vert {\tau _{+}^{s}}{\tau _{-}^{1/2}}|{\textbf{J}}^{{{\widetilde{S}}_{i}}}|w_{{\overline{\mu }}}^{1/2}\right\Vert _2\nonumber \\{} & {} + \left\Vert {\tau _{0}^{s-1/2-{{\overline{\mu }}}}}{\tau _{-}^{s+1/2}}|{\textbf{J}}^{{{\widetilde{L}}}}| w_{{\overline{\mu }}}^{1/2}\right\Vert _2. \end{aligned}$$

If we write ${\textbf{J}}= {\textbf{J}}[{\textbf{G}}]$, where ${\textbf{G}}$ is a two-form, and ${\textbf{G}}^0$ and ${\textbf{G}}^1$ are the charged and charge-free part of ${\textbf{G}}$, respectively, then we define the decomposition

$$\begin{aligned} {\textbf{J}}^0[{\textbf{G}}] = {\textbf{J}}[{{\textbf{G}}^0}],\quad {\textbf{J}}^1[{\textbf{G}}] = {\textbf{J}}[{{\textbf{G}}^1}]. \end{aligned}$$

#### Lemma 8.1

If $({\textbf{F}}, \varvec{\phi })$ solve the system (1.14) on an interval $[0,T]\times {\mathbb {R}}^3$ and satisfy the bootstrap assumption (7.1), then

8.9 $$\begin{aligned} \sum _{\begin{array}{c} |I| \le k \end{array}}\left\Vert {\textbf{J}}[{\mathcal {L}}_{{\widetilde{X}}}^I {\textbf{F}}^1] \right\Vert _{L^2[w]} \lesssim |q|+{\mathcal {E}}_k(T) + \varepsilon _g{\mathcal {E}}^{1/2}_k(T). \end{aligned}$$

#### Proof

It suffices to show (8.9) holds if ${\textbf{F}}^1$ is replaced by ${\textbf{F}}$ or ${\textbf{F}}^0$. We recall the identity (2.39), which we iterate and use (2.29) to get

8.10 $$\begin{aligned}{} & {} |{\textbf{J}}[{\mathcal {L}}_{{\widetilde{X}}}^I {\textbf{F}}^1]^{\alpha } - ({\mathcal {L}}_{{\widetilde{X}}}^I{\textbf{J}}^1)^\alpha | \nonumber \\{} & {} \quad \lesssim \sum _{|I_1|+|I_2|+1 = |I|}\Big |{\mathcal {L}}_{{\widetilde{X}}}^{I_1}\Big ((\nabla _\beta (g^{\gamma \delta }({\mathcal {L}}_{{\widetilde{X}}}g)_{\gamma \delta })({\mathcal {L}}_{{\widetilde{X}}}^{I_2}({\textbf{F}}^{1\sharp }))^{\alpha \beta }\Big )\Big |, \end{aligned}$$

On the right-hand side of Eq. (8.10) we can replace $({\mathcal {L}}_{{\widetilde{X}}}g)_{\gamma \delta }$ with ${{^{({X})}{\widetilde{\pi }}}}_{\gamma \delta }$, as the difference is the derivative of a constant.

#### The estimate on ${\textbf{J}}$

We can expand the first term out as

8.11 $$\begin{aligned} ({\mathcal {L}}_{{\widetilde{X}}}^I{\textbf{J}})^\alpha = {\mathcal {L}}_{{\widetilde{X}}}^I\left( g^{\alpha \beta }{\mathfrak {I}}\left( \varvec{\phi }\overline{D_\beta \varvec{\phi }}\right) \right) . \end{aligned}$$

When derivatives fall on *g*, at each step we write $({\mathcal {L}}_{{\widetilde{X}}}g)^{\alpha \beta } =( \widetilde{{\mathcal {L}}}_{{\widetilde{X}}}g)^{\alpha \beta } - c_{{\widetilde{X}}}g^{\alpha \beta }$. The identities (1.1) and (2.43a) imply

8.12 $$\begin{aligned} {\mathcal {L}}_{{\widetilde{X}}}(\phi \overline{D\psi })_{\alpha } = D_{{\widetilde{X}}}\phi \overline{D_\alpha \psi } + \phi (\overline{{{\mathcal {L}}_{{\widetilde{X}}}^{{\mathbb {C}}{ }}}D\phi })_{\alpha }. \end{aligned}$$

Iterating and symmetrizing this gives us, for any vector $\widetilde{U}$,

8.13 $$\begin{aligned} \left| {\mathfrak {I}}({\mathcal {L}}_{{\widetilde{X}}}^I(\varvec{\phi }\overline{D\varvec{\phi }}))_{\widetilde{U}}\right|&\lesssim \sum _{\begin{array}{c} |I_1|+|I_2|\le |I| \end{array}}\left| {\mathfrak {I}}\left( D_{{\widetilde{X}}}^{I_1}\varvec{\phi }\overline{D_{\widetilde{U}} D_{{\widetilde{X}}}^{I_2}\varvec{\phi }}+ D_{{\widetilde{X}}}^{I_2}\varvec{\phi }\overline{D_{\widetilde{U}} D_{{\widetilde{X}}}^{I_1}\varvec{\phi }}\right) \right| \nonumber \\&\quad + \sum _{\begin{array}{c} |I_1|+|I_2|+|I_3|+1 \le |I| \end{array}}\left| D_{{\widetilde{X}}}^{I_1}\varvec{\phi }\overline{D_{{\widetilde{X}}}^{I_2}\varvec{\phi }}|({\mathcal {L}}_{{\widetilde{X}}}^{I_3}{\textbf{F}})_{{\widetilde{X}}{\widetilde{U}}}\right| . \end{aligned}$$

We note the identity

8.14 $$\begin{aligned}{} & {} {\mathfrak {I}}\left( D_{{\widetilde{X}}}^{I_1}\varvec{\phi }\overline{D_U D_{{\widetilde{X}}}^{I_2}\varvec{\phi }}+ D_{{\widetilde{X}}}^{I_2}\varvec{\phi }\overline{D_U D_{{\widetilde{X}}}^{I_1}\varvec{\phi }}\right) \nonumber \\{} & {} \quad = {\mathfrak {I}}\left( D_{{\widetilde{X}}}^{I_1}\varvec{\phi }\tfrac{\overline{D_U (r^*D_{{\widetilde{X}}}^{I_2}\varvec{\phi })}}{r^*}+ D_{{\widetilde{X}}}^{I_2}\varvec{\phi }\tfrac{\overline{D_U (r^*D_{{\widetilde{X}}}^{I_1}\varvec{\phi })}}{r^*}\right) , \end{aligned}$$

which follows from (1.10) on the $D_U$ derivative on the right-hand side and noting

$$\begin{aligned} {\mathfrak {I}}\left( D_{{\widetilde{X}}}^{I_1}\varvec{\phi }\overline{D_{{\widetilde{X}}}^{I_2}\varvec{\phi }}+ D_{{\widetilde{X}}}^{I_2}\varvec{\phi }\overline{D_{{\widetilde{X}}}^{I_1}\varvec{\phi }}\right) = 0 \end{aligned}$$

We are ready to show energy and decay bounds, with the latter necessary to control energy terms coming from the metric. We combine (6.42) and (8.1) to get, for $|I|\le k-4$,

8.15a $$\begin{aligned} |{\mathcal {L}}_{{\widetilde{X}}}^I{\mathfrak {I}}\left( \varvec{\phi }\overline{D\varvec{\phi }}\right) _{{{\widetilde{L}}}}|&\lesssim {\mathcal {E}}_k(T){\tau _{+}^{-5/2-s}}{\tau _{-}^{1/2-s}}w^{-1}, \end{aligned}$$

8.15b $$\begin{aligned} |{\mathcal {L}}_{{\widetilde{X}}}^I{\mathfrak {I}}\left( \varvec{\phi }\overline{D\varvec{\phi }}\right) _{{{\widetilde{S}}_{i}}}|&\lesssim {\mathcal {E}}_k(T){\tau _{+}^{-2-s}}{\tau _{-}^{-s}}w^{-1}, \end{aligned}$$

8.15c $$\begin{aligned} |{\mathcal {L}}_{{\widetilde{X}}}^I{\mathfrak {I}}\left( \varvec{\phi }\overline{D\varvec{\phi }}\right) _{{\underline{{\widetilde{L}}}}}|&\lesssim {\mathcal {E}}_k(T){\tau _{+}^{-2}}{\tau _{-}^{-2s}}w^{-1}. \end{aligned}$$

We recall the decomposition of Lie derivatives of the inverse metric, ${\mathcal {L}}_{{\widetilde{X}}}g^{-1} = \widetilde{{\mathcal {L}}}_{X}g^{-1} - c_Xg^{-1}$, as well as the term ${{^{({X^I})}{\widetilde{\Pi }}}} = \widetilde{{\mathcal {L}}}_{X}^Ig^{-1}$. We can then take the pointwise estimate

8.16 $$\begin{aligned} (|{\mathcal {L}}_{{\widetilde{X}}}^I{\textbf{J}})^\alpha |\lesssim & {} \sum _{|I'|\le |I|}|g^{\alpha \beta }({\mathcal {L}}_{{\widetilde{X}}}^{I'}{\mathfrak {I}}(\varvec{\phi }\overline{D\varvec{\phi }}))_\beta |\nonumber \\{} & {} + \sum _{|I_1|+|I_2|\le |I|}|{{^{({I_1})}{\widetilde{\Pi }}}}^{\alpha \beta }({\mathcal {L}}_{{\widetilde{X}}}^{I_2}{\mathfrak {I}}(\varvec{\phi }\overline{D\varvec{\phi }}))_\beta |. \end{aligned}$$

To bound the second term, we first take

8.17 $$\begin{aligned}{} & {} \sum _{\begin{array}{c} |I_1| + |I_2| \le |I| \\ |I_2|\le k-6 \end{array}}\left\Vert |{{^{({I_1})}{\widetilde{\Pi }}}}^{\alpha \beta }||{\mathcal {L}}_{{\widetilde{X}}}^{I_2}{\mathfrak {I}}(\varvec{\phi }\overline{D\varvec{\phi }})_\beta |{\tau _{+}^{s+1/2+{{\overline{\mu }}}}}{\tau _{-}^{-{{\overline{\mu }}}}}w_{{\overline{\mu }}}^{1/2}\right\Vert _2\nonumber \\{} & {} \quad \lesssim {\mathcal {E}}_k(T)\sum _{|I|\le k}\left\Vert {\tau _{+}^{s+1/2+{{\overline{\mu }}}-2}}{\tau _{-}^{-2s}}{{^{({{\widetilde{X}}^I})}{\widetilde{\Pi }}}}\right\Vert _2. \end{aligned}$$

Using $s+1/2+{{\overline{\mu }}}-2 < -1/2-\frac{\delta }{2}$, $-2\,s < -1$, we can easily bound this by $C{\mathcal {E}}_k(T)\varepsilon _g$ using (2.25a).

When we can take $L^\infty$ estimates on the metric, we can lower indices easily, as all error terms correspond with metric terms with decay faster than ${\tau _{0}^{2s}}$, the difference in weights between the highest and lowest weights in the norm (8.8). We first consider the inequality

$$\begin{aligned} w_{{\overline{\mu }}}^{1/2}\lesssim {\tau _{-}^{1/2+2{{\overline{\mu }}}}}(w')^{1/2}, \end{aligned}$$

which we combine with (8.8) to get

$$\begin{aligned} \left\Vert {\textbf{J}}\right\Vert _{L^2[w]}\lesssim & {} \left\Vert {\tau _{+}^{s+1/2+{{\overline{\mu }}}}}{\tau _{-}^{1/2+{{\overline{\mu }}}}}|{\textbf{J}}_{{\widetilde{L}}}|(w')^{1/2}\right\Vert _2\nonumber \\{} & {} + \left\Vert {\tau _{0}^{s-1/2-{{\overline{\mu }}}}}{\tau _{-}^{s+1+2{{\overline{\mu }}}}}|{\textbf{J}}_{{\underline{{\widetilde{L}}}}}|(w')^{1/2}\right\Vert _2 + \left\Vert {\tau _{+}^{s}}{\tau _{-}^{1+2{{\overline{\mu }}}}}|{\textbf{J}}_{{{\widetilde{S}}_{i}}}|(w')^{1/2}\right\Vert _2. \end{aligned}$$

We then combine the identity (8.13) with the $L^2$ norm (8.7) and the ${\mathcal {L}}_{{\widetilde{X}}}^I\varvec{\phi }$ term in the $L^2$ norm $S_0[D_{{\widetilde{X}}}^I\varvec{\phi }](T)$ as well as the $L^\infty$ norms (8.1) and (6.42) to get our desired estimate. Note that for each component estimate we use the inequalities $w^{-1/2}\lesssim 1, \quad 1/2+2\mu -s < 0,\quad {\tau _{-}^{\delta }}\le {\tau _{+}^{\delta }}, \quad {\tau _{-}^{s-1/2}}\le {\tau _{+}^{s-1/2}}$. We take the ${{\widetilde{S}}_{i}}$ components as an example; other cases follow similarly. We first take, for $|I|\le k$,

8.18 $$\begin{aligned}&\sum _{|I|\le k}\left\Vert {\tau _{+}^{s}}{\tau _{-}^{1+2{{\overline{\mu }}}}}|({\mathcal {L}}_{{\widetilde{X}}}^I{\textbf{J}})_{{{\widetilde{S}}_{i}}}|(w')^{1/2}\right\Vert _2 \nonumber \\&\quad \lesssim \sum _{\begin{array}{c} |I_2|\le k\\ |I_1|\le k-5 \end{array}}{\left\Vert {{\tau _{+}^{}}{\tau _{-}^{s-1/2}}D_{{\widetilde{X}}}^{I_1}\varvec{\phi }}\right\Vert }_\infty {\left\Vert {{\tau _{+}^{s-1}}{\tau _{-}^{3/2-s+2{{\overline{\mu }}}}}({\mathcal {L}}_{{\widetilde{X}}}^{I_2}D\varvec{\phi })_{{{\widetilde{S}}_{i}}}(w')^{1/2}}\right\Vert }_2 \nonumber \\&\qquad +\sum _{\begin{array}{c} |I_1|\le k\\ |I_2|\le k-5 \end{array}}{\left\Vert {{\tau _{+}^{-1}} {\tau _{-}^{1/2+2{{\overline{\mu }}}}}D_{{\widetilde{X}}}^{I_1}\varvec{\phi }(w')^{1/2}}\right\Vert }_2{\left\Vert {{\tau _{+}^{1+s}}{\tau _{-}^{1/2}}({\mathcal {L}}_{{\widetilde{X}}}^{I_2}D\varvec{\phi })_{{{\widetilde{S}}_{i}}}}\right\Vert }_\infty \nonumber \\&\quad \lesssim {{\mathcal {E}}}_k(T), \end{aligned}$$

using the inequalities

$$\begin{aligned} {\tau _{+}^{s-1}}{\tau _{-}^{3/2-s+2{{\overline{\mu }}}}} = {\tau _{+}^{s}}{\tau _{0}^{}}{\tau _{-}^{1/2+2{{\overline{\mu }}}-s}}\le {\tau _{+}^{s}}{\tau _{0}^{1/2+{{\overline{\mu }}}}} \end{aligned}$$

and

$$\begin{aligned} {\tau _{+}^{-1}}{\tau _{-}^{1/2+2{{\overline{\mu }}}}} = {\tau _{+}^{s-1}}{\tau _{0}^{1/2+{{\overline{\mu }}}}}{\tau _{-}^{{\overline{\mu }}}}{\tau _{+}^{1/2+{{\overline{\mu }}}-s}}\le {\tau _{+}^{s-1}}{\tau _{0}^{1/2+{{\overline{\mu }}}}}. \end{aligned}$$

Repeating this for each component gives the estimate

8.19 $$\begin{aligned} \sum _{|I|\le k}\big \Vert {\mathcal {L}}_{{\widetilde{X}}}^I{\textbf{J}}\big \Vert _{L^2[w]} \lesssim {\mathcal {E}}_k(T). \end{aligned}$$

#### The estimate on ${\textbf{J}}^0$

We now look at ${\textbf{J}}^{0a}$. It is easiest to calculate this with respect to the modified frame $\{{\widetilde{\partial }}_a\}$. Defining $\widetilde{g}$ and $\widetilde{\Gamma }$ to be the metric coefficients and Christoffel symbols of *g* with respect to this frame, the identities

$$\begin{aligned} \widetilde{\Gamma }^b_{bc} = \tfrac{1}{2}{\widetilde{\partial }}_c\ln |{\widetilde{g}}|, \quad \widetilde{\Gamma }^\alpha _{\beta \gamma }{{\textbf{F}}^0}^{\beta \gamma }=0. \end{aligned}$$

imply

8.20 $$\begin{aligned} {\textbf{J}}^{0a} = \nabla _b {{\textbf{F}}^0}^{ab} = \tfrac{1}{2}{\widetilde{\partial }}_b{(\ln |\widetilde{g}|)}{{\textbf{F}}^0}^{ab} + {\widetilde{\partial }}_b{{\textbf{F}}^0}^{ab} \end{aligned}$$

We define

8.21 $$\begin{aligned} {{\widehat{{\textbf{F}}}}^{ab}} = {{\widehat{m}}}^{ac}{{\widehat{m}}}^{bd}{\textbf{F}}^0_{cd}, \quad {\widehat{{\textbf{J}}}}^a = {\widetilde{\partial }}_b{{\widehat{{\textbf{F}}}}^{ab}} \end{aligned}$$

Direct computation gives

8.22 $$\begin{aligned} {\widehat{{\textbf{J}}}}^a = \Big (\tfrac{q{{\overline{\chi }}}'}{4\pi r^{*2}}\Big ){{\widetilde{L}}}^a. \end{aligned}$$

Iterating (2.11), noting that ${\widehat{{\textbf{J}}}}$ is compactly supported in $t-r^*$, and taking the null decomposition gives the estimate

8.23 $$\begin{aligned} {\tau _{+}^{2}}|({\mathcal {L}}_{{\widetilde{X}}}^I{\widehat{{\textbf{J}}}})^{\underline{{\widetilde{L}}}}| + {\tau _{+}^{}}(|({\mathcal {L}}_{{\widetilde{X}}}^I{\widehat{{\textbf{J}}}})^{{{\widetilde{S}}_{1}}}| + |({\mathcal {L}}_{{\widetilde{X}}}^I{\widehat{{\textbf{J}}}})^{{{\widetilde{S}}_{2}}}|) + |({\mathcal {L}}_{{\widetilde{X}}}^I{\widehat{{\textbf{J}}}})^{\underline{{\widetilde{L}}}}| \lesssim |q|{\tau _{+}^{-2}}. \end{aligned}$$

It follows from direct integration (again noting that ${\widehat{{\textbf{J}}}}$ is supported close to the light cone) that

8.24 $$\begin{aligned} \big \Vert ({\mathcal {L}}_{{\widetilde{X}}}^I{\widehat{{\textbf{J}}}})\big \Vert _{L^2[w]}\lesssim |q|. \end{aligned}$$

We note generally that, for all tensorial quantities $\textbf{K}$ which satisfy

8.25 $$\begin{aligned} |{\textbf{K}}^a|\lesssim {\tau _{+}^{-3+\delta }}{\tau _{-}^{-1}} \end{aligned}$$

in all components, direct integration gives the bound

8.26 $$\begin{aligned} \big \Vert {\textbf{K}}\big \Vert _{L^2[w]} \le C. \end{aligned}$$

We may use this to bound all terms in

8.27 $$\begin{aligned} {\textbf{J}}_{rem}^a = {\widetilde{\partial }}_b{\textbf{F}}^{0ab} - {\widehat{{\textbf{J}}}}^a \end{aligned}$$

where the most derivatives fall on ${\textbf{F}}^0$ or $m_0 - {{\widehat{m}}}$. Therefore, a straightforward calculation using (2.24) and (2.23) gives

$$\begin{aligned} \sum _{|I|\le k}\big \Vert {\mathcal {L}}_{X}^I({\textbf{J}}_{rem})^a\big \Vert _{L^2[w]}\lesssim & {} \varepsilon _g|q| + |q|\Vert {\tau _{+}^{s+1/2+{{\overline{\mu }}}}}{\tau _{-}^{-{{\overline{\mu }}}+ \gamma }}{\tau _{+}^{-2}}\\{} & {} (|{\widetilde{\partial }}{\mathcal {L}}_{X}^I H_0| + {\tau _{-}^{-1}}{\tau _{+}^{-1+\delta }}|H_0|)\Vert _2 \lesssim \varepsilon _g|q|. \end{aligned}$$

The bound on ${\widetilde{\partial }}_b{(\ln |\widetilde{g}|)}{{\textbf{F}}^0}^{ab}$ follows similarly (using (2.22) and fast decay of derivatives of the coefficient transformation matrix). For sufficiently small $\varepsilon _1$, we therefore have

8.28 $$\begin{aligned} \sum _{|I|\le k}\big \Vert {\mathcal {L}}_{{\widetilde{X}}}^I{\textbf{J}}^0\big \Vert _{L^2[w]}\lesssim |q| \end{aligned}$$

#### Bounding the Commutator Terms

To conclude the proof of Theorem 8.1, it suffices to bound the right-hand side of (8.10). We recall the identity (2.34), and the consequent pointwise bound

8.29 $$\begin{aligned} {\mathcal {L}}_{{\widetilde{X}}}^I\nabla _\beta (g^{\gamma \delta }(\widetilde{{\mathcal {L}}}_{{{\widetilde{X}}}}g)_{\gamma \delta })\lesssim & {} \sum _{|J| \le |I|+1}\nabla _\beta (g^{\gamma \delta }(\widetilde{{\mathcal {L}}}_{{{\widetilde{X}}}}^J g)_{\gamma \delta }) \nonumber \\{} & {} + \sum _{|J| + |K| \le |I|+1}\nabla _\beta ((\widetilde{{\mathcal {L}}}_{{{\widetilde{X}}}}^Jg)^{\gamma \delta }(\widetilde{{\mathcal {L}}}_{{{\widetilde{X}}}}^Kg)_{\gamma \delta }). \end{aligned}$$

Therefore, for $$|I|+1 \le k - 6$$,

8.30a $$\begin{aligned} |{{\widetilde{L}}}^\alpha {\mathcal {L}}_{{\widetilde{X}}}^I\nabla _\alpha (g^{\gamma \delta }(\widetilde{{\mathcal {L}}}_{X}g)_{\gamma \delta })|&\lesssim \varepsilon _g{\tau _{+}^{-2+\delta }}, \end{aligned}$$

8.30b $$\begin{aligned} |{{\widetilde{S}}_{i}}^{\alpha }{\mathcal {L}}_{{\widetilde{X}}}^I\nabla _\alpha (g^{\gamma \delta }(\widetilde{{\mathcal {L}}}_{X}g)_{\gamma \delta })|&\lesssim \varepsilon _g{\tau _{+}^{-2+\delta }}, \end{aligned}$$

8.30c $$\begin{aligned} |{\underline{{\widetilde{L}}}}^{\alpha }{\mathcal {L}}_{{\widetilde{X}}}^I\nabla _\alpha (g^{\gamma \delta }(\widetilde{{\mathcal {L}}}_{X}g)_{\gamma \delta })|&\lesssim \varepsilon _g{\tau _{-}^{-1}}{\tau _{+}^{-1+\delta }}. \end{aligned}$$

If $|I|\le k$, a repeated application of (2.34) combined with the estimates (2.25) and (2.24) imply

8.31a $$\begin{aligned} \left\Vert {\tau _{+}^{-\delta }}{{\widetilde{L}}}^\alpha {\mathcal {L}}_{{\widetilde{X}}}^I\nabla _\alpha (g^{\gamma \delta }(\widetilde{{\mathcal {L}}}_{X}g)_{\gamma \delta })(w_g')^{1/2}\right\Vert _2 \lesssim \varepsilon _g, \end{aligned}$$

8.31b $$\begin{aligned} \left\Vert {\tau _{+}^{-\delta }}{{\widetilde{S}}_{i}}^\alpha {\mathcal {L}}_{{\widetilde{X}}}^I\nabla _\alpha (g^{\gamma \delta }(\widetilde{{\mathcal {L}}}_{X}g)_{\gamma \delta })(w_g')^{1/2}\right\Vert _2 \lesssim \varepsilon _g, \end{aligned}$$

8.31c $$\begin{aligned} \left\Vert {\tau _{+}^{-1/2-\delta }}{\underline{{\widetilde{L}}}}^\alpha {\mathcal {L}}_{{\widetilde{X}}}^I\nabla _\alpha (g^{\gamma \delta }(\widetilde{{\mathcal {L}}}_{X}g)_{\gamma \delta })\right\Vert _2 \lesssim \varepsilon _g. \end{aligned}$$

In all cases, if Lie derivatives fall on $g^{\gamma \delta }$ we have terms which are quadratic and easy to bound using (2.25) and (2.24). Otherwise, for (8.31a) and (8.31b) this follows from the spacetime bound (2.25b) combined with the estimates ${\tau _{-}^{-1/2-\mu }}w < (w_g')^{1/2}$ and a dyadic decomposition in $1+t$ and for (8.31c) it follows from bounding the square of the $L^2$ norm by $\varepsilon _g(1+t)^{-1+\delta }$ and integrating.

Now we look at the case when Lie derivatives fall on ${\textbf{F}}^{1\sharp }$. Taking a null decomposition and combining the bounds (2.24) and (5.17), as well as ${\tau _{-}^{-{{\overline{\mu }}}}}w_{{\overline{\mu }}}\lesssim w$, gives the following:

8.32a $$\begin{aligned} \sum _{|J| \le k-6}\big |({\mathcal {L}}_{{\widetilde{X}}}^{J}{\textbf{F}}^{1\sharp })^{{{\widetilde{L}}}{\underline{{\widetilde{L}}}}}\big |&\lesssim {\tau _{+}^{-s-1}}{\tau _{-}^{-1/2+{{\overline{\mu }}}}}w_{{\overline{\mu }}}^{-1/2}{\mathcal {E}}_k[{\textbf{F}}](T)^{1/2}, \end{aligned}$$

8.32b $$\begin{aligned} \sum _{|J| \le k-6}\big |({\mathcal {L}}_{{\widetilde{X}}}^{J}{\textbf{F}}^{1\sharp })^{{{\widetilde{L}}}{{\widetilde{S}}_{}}}\big |&\lesssim {\tau _{+}^{-1}}{\tau _{-}^{-1/2-s+{{\overline{\mu }}}}}w_{{\overline{\mu }}}^{-1/2}{\mathcal {E}}_k[{\textbf{F}}](T)^{1/2}, \end{aligned}$$

8.32c $$\begin{aligned} \sum _{|J| \le k-6}\big |({\mathcal {L}}_{{\widetilde{X}}}^{J}{\textbf{F}}^{1\sharp })^{{\underline{{\widetilde{L}}}}{{\widetilde{S}}_{}}}\big |&\lesssim {\tau _{+}^{-3/2-s}}{\tau _{-}^{{{\overline{\mu }}}}}w_{{\overline{\mu }}}^{-1/2}{\mathcal {E}}_k[{\textbf{F}}](T)^{1/2}, \end{aligned}$$

8.32d $$\begin{aligned} \sum _{|J| \le k-6}\big |({\mathcal {L}}_{{\widetilde{X}}}^{J}{\textbf{F}}^{1\sharp })^{{{\widetilde{S}}_{1}}{{\widetilde{S}}_{2}}}\big |&\lesssim {\tau _{+}^{-s-1}}{\tau _{-}^{1/2+{{\overline{\mu }}}}}w_{{\overline{\mu }}}^{-1/2}{\mathcal {E}}_k[{\textbf{F}}](T)^{1/2}, \end{aligned}$$

We also have the $L^2$ norms

8.33a $$\begin{aligned} \sum _{|J| \le k}\left\Vert {\tau _{+}^{s}}{\tau _{0}^{1/2+{{\overline{\mu }}}}}{\tau _{-}^{-1/2-2{{\overline{\mu }}}}}({\mathcal {L}}_{{\widetilde{X}}}^{J}{\textbf{F}}^{1\sharp })^{{{\widetilde{L}}}{\underline{{\widetilde{L}}}}}w_{{\overline{\mu }}}^{1/2}\right\Vert _2&\lesssim {\mathcal {E}}_k^{1/2}, \end{aligned}$$

8.33b $$\begin{aligned} \sum _{|J| \le k}\left\Vert {\tau _{-}^{s-1/2-2{{\overline{\mu }}}}}{\tau _{0}^{1/2+{{\overline{\mu }}}}}({\mathcal {L}}_{{\widetilde{X}}}^{J}{\textbf{F}}^{1\sharp })^{{{\widetilde{L}}}{{\widetilde{S}}_{i}}}w_{{\overline{\mu }}}^{1/2}\right\Vert _2&\lesssim {\mathcal {E}}_k^{1/2}, \end{aligned}$$

8.33c $$\begin{aligned} \sum _{|J| \le k}\left\Vert {\tau _{+}^{s}}{\tau _{0}^{1/2+{{\overline{\mu }}}}}{\tau _{-}^{-1/2-2{{\overline{\mu }}}}}({\mathcal {L}}_{{\widetilde{X}}}^{J}{\textbf{F}}^{1\sharp })^{{{\widetilde{S}}_{1}}{{\widetilde{S}}_{2}}}w_{{\overline{\mu }}}^{1/2}\right\Vert _2&\lesssim {\mathcal {E}}_k^{1/2}, \end{aligned}$$

8.33d $$\begin{aligned} \sum _{|J| \le k}\left\Vert {\tau _{+}^{s}}{\tau _{-}^{-1/2-2{{\overline{\mu }}}}}({\mathcal {L}}_{{\widetilde{X}}}^{J}{\textbf{F}}^{1\sharp })^{{\underline{{\widetilde{L}}}}{{\widetilde{S}}_{i}}}w_{{\overline{\mu }}}^{1/2}\right\Vert _2&\lesssim {\mathcal {E}}_k^{1/2}. \end{aligned}$$

In order to obtain these bounds, we first decompose $g^{\alpha \beta } = m_0^{\alpha \beta }+H_1^{\alpha \beta }$. Then, if more derivatives fall on ${\textbf{F}}^{1\sharp }$, we use the estimates (2.24) and (2.23) and a null decomposition, noting the intermediate bound

8.34 $$\begin{aligned} |{\textbf{G}}^{{\underline{{\widetilde{L}}}}{{\widetilde{S}}_{i}}}|\lesssim |{\alpha }[{\textbf{G}}]| + \varepsilon _g{\tau _{+}^{-1+\delta }}(|\rho [{\textbf{G}}]|+|\sigma [{\textbf{G}}]|) + \varepsilon _g{\tau _{0}^{\gamma }}{\tau _{+}^{-1+\delta }}|{\underline{\alpha }}[{\textbf{G}}]|. \end{aligned}$$

When more derivatives fall on $H_1$, then (8.33a)–(8.33c) follow from (5.17) and (2.25a), with no null decomposition necessary (using the bounds $s-\tfrac{1}{2}-{{\overline{\mu }}} - 1 < -\tfrac{1}{2}-\delta$, squaring, and integrating in time). For the estimate (8.33d), we use a null decomposition as well as the bound (2.25c) to bound terms containing $|{\underline{\alpha }}||H_0^{{\underline{{\widetilde{L}}}}{\underline{{\widetilde{L}}}}}|$.

In order to bound the right-hand side of (8.10), we take a null decomposition and apply the estimates (8.30), (8.31), (8.32), and (8.33). Combining this with (8.19) and (8.28) completes the proof of Theorem 8.1. $\square$

## Commutator Estimates for $\varvec{\phi }$

Here we will bound the remaining term in Theorem 7.1.

### Lemma 9.1

For a pair $(\varvec{\phi }, {\textbf{F}})$ satisfying (1.14), as well as the restrictions of Theorem 1.1 and the bootstrap assumption (7.1), then for sufficiently small $\varepsilon _b$, and $\varepsilon _g \le 1$, the estimate

9.1 $$\begin{aligned} \sum _{\begin{array}{c} |I| \le k \end{array}}\left\Vert \left( \Box ^{{\mathbb {C}}}_gD_{{\widetilde{X}}}^I\varvec{\phi }\right) {\tau _{+}^{s}}{\tau _{-}^{1/2}}w_{{\overline{\mu }}}^{1/2}\right\Vert _2 \lesssim {\mathcal {E}}_k(T) + \varepsilon _g{\mathcal {E}}_k(T)^{1/2}. \end{aligned}$$

For ease of notation, we say

$$\begin{aligned}{} & {} {\sum _{\le k}}\,T[{\widetilde{X}}^I, {\widetilde{X}}^{I_1},\ldots ,{\widetilde{X}}^{I_m},\,{\widetilde{Y}}_1,\ldots ,{\widetilde{Y}}_n] \\{} & {} \quad = \sum _{|I|+|I_1|+\ldots +|I_m|+n\le k}\,T[{\widetilde{X}}^{I},{\widetilde{X}}^{I_1},\ldots {\widetilde{X}}^{I_m},\,{\widetilde{Y}}_1,\ldots ,{\widetilde{Y}}_n], \end{aligned}$$

where all ${\widetilde{X}}, {\widetilde{Y}}\in \{{\widetilde{\partial }}_\alpha , {{\widetilde{\Omega }}_{{\alpha \beta }}}, {{\widetilde{S}}}\}$, and $I, \{I_k\}$ are multiindices of vector fields in this set. Since $({\textbf{F}}, \varvec{\phi })$ solves (1.14), it suffices to prove

9.2 $$\begin{aligned} \left\Vert {\tau _{+}^{s}}{\tau _{-}^{1/2}}\left( \left[ \Box ^{{\mathbb {C}}}_g, D_{{\widetilde{X}}}^I\right] \varvec{\phi }\right) w_{{\overline{\mu }}}^{1/2}\right\Vert _2 \lesssim {\mathcal {E}}_k(T) + \varepsilon _g{\mathcal {E}}_k(T)^{1/2}. \end{aligned}$$

We may iterate the identity (2.44) to obtain

9.3 $$\begin{aligned} \left| \left[ \Box _g^{\mathbb {C}}, D_{{\widetilde{X}}}^I\right] \varvec{\phi }\right| \lesssim A_{|I|}+B_{|I|}+C_{|I|}, \end{aligned}$$

where

9.4a $$\begin{aligned} A_j&:= \sum _{|I_1| + |I_2| \le j-1}\left| D_{{\widetilde{X}}}^{I_1}\left( D^\alpha {D_{{\widetilde{X}}}^{I_2}}\varvec{\phi }\nabla _\alpha (\nabla \cdot {\widetilde{Y}})\right) \right| \end{aligned}$$

9.4b $$\begin{aligned} B_j&:= \sum _{|I_1| + |I_2| \le j-1} \left| D_{{\widetilde{X}}}^{I_1}\left( D_\alpha \left( {{^{({{\widetilde{Y}}})}{\Pi }}}^{\alpha \beta }D_\beta {D_{{\widetilde{X}}}^{I_2}}\varvec{\phi }\right) \right) \right| \end{aligned}$$

9.4c $$\begin{aligned} C_j&:= \sum _{|I_1| + |I_2| \le j-1}\left| D_{{\widetilde{X}}}^{I_1}\left( i(\nabla ^\alpha F_{{\widetilde{Y}}\alpha }{D_{{\widetilde{X}}}^{I_2}}\varvec{\phi }+ 2F_{{\widetilde{Y}}\alpha }D^\alpha {D_{{\widetilde{X}}}^{I_2}}\varvec{\phi })\right) \right| \end{aligned}$$

We briefly explain how we bound each term in our weighted $L^2$ norm, as this will be the focus of the remainder of this section. First, $A_{|I|}$ is quadratic. In order to bound $B_{|I|}$, we subtract off a lower-order term (which we can bound by $A_{|I-1|}, B_{|I-1|}, C_{|I-1|}$), then bound the remainder using the improved decay estimates coming from the modified coordinates. Finally, to bound $C_{|I|}$, we must use a special structure of the MKG system which shows up also in the Minkowski case. The result follows from induction, using $A_0 = B_0 = C_0 = 0$.

### Bounding $A_{|I|}$

In order to bound $A_{|I|}$, we iterate (2.43b) to get:

9.5 $$\begin{aligned} {\sum _{\le j}}\left| {\mathcal {L}}_{{{\widetilde{X}}}_1}^{I_1}\left( D^\alpha {D_{{{\widetilde{X}}}_2}^{I_2}}\varvec{\phi }\nabla _\alpha (\nabla \cdot {{\widetilde{Y}}})\right) \right| \lesssim A^1_{j} + A^2_{j} + A^3_{j}, \end{aligned}$$

where

9.6a $$\begin{aligned} A^1_{j}&= {\sum _{\le j}}\left| g^{\alpha \beta }D_{\alpha }D_{{{\widetilde{X}}}_1}^{I_1}\varvec{\phi }\nabla _\beta {\mathcal {L}}_{{{\widetilde{X}}}_2}^{I_2}(g^{\gamma \delta }(\widetilde{{\mathcal {L}}}_{{{\widetilde{Y}}}}g)_{\gamma \delta }) \right| \end{aligned}$$

9.6b $$\begin{aligned} A^2_{j}&= {\sum _{\le j}}\left| {{^{({{{\widetilde{X}}}_1^{I_1}})}{\widetilde{\Pi }}}}^{\alpha \beta }D_\alpha D_{{{\widetilde{X}}}_2}^{I_2}\varvec{\phi }\nabla _{\beta }{\mathcal {L}}_{{{\widetilde{X}}}_3}^{I_3}(g^{\gamma \delta }(\widetilde{{\mathcal {L}}}_{{{\widetilde{Y}}}}g)_{\gamma \delta }) \right| \end{aligned}$$

9.6c $$\begin{aligned} A^3_{j}&={\sum _{\le j}}\left| {{^{({{{\widetilde{X}}}_1^{I_1}})}{\Pi }}}^{\alpha \beta } ({\mathcal {L}}_{{{\widetilde{X}}}_2}^{I_2}{\textbf{F}})_{{{\widetilde{Y}}}_1\alpha } D_{{{\widetilde{X}}}_3}^{I_3}\varvec{\phi }\nabla _{\beta }{\mathcal {L}}_{{{\widetilde{X}}}_4}^{I_4}(g^{\gamma \delta }(\widetilde{{\mathcal {L}}}_{{{\widetilde{Y}}}_2}g)_{\gamma \delta })\right| . \end{aligned}$$

$A_j^1$ consists of terms where the derivative commutes through $D^\alpha$, and $A_j^2$ and $A_j^3$ consist of the commutator terms. To bound $A^1_j$, we take the null decomposition

$$\begin{aligned} {\sum _{\le j}}\left| g^{\alpha \beta }D_{\alpha }D_{{{\widetilde{X}}}}^{I_1}\varvec{\phi }\nabla _\beta {\mathcal {L}}_{{{\widetilde{X}}}}^{I_2}(g^{\gamma \delta }(\widetilde{{\mathcal {L}}}_{Y}g)_{\gamma \delta }) \right|\lesssim & {} {\sum _{\le j}}|DD_{{{\widetilde{X}}}}^{I_1}\varvec{\phi }||{{\overline{\partial }}} \widetilde{{\mathcal {L}}}_{{{\widetilde{X}}}}^{I_2}g| \\{} & {} + |{\overline{D}}D_{{{\widetilde{X}}}}^{I_1}\varvec{\phi }||\partial \widetilde{{\mathcal {L}}}_{{{\widetilde{X}}}}^{I_2}g| + |g^{{\underline{{\widetilde{L}}}}{\underline{{\widetilde{L}}}}}||DD_{{{\widetilde{X}}}}^{I_1}\varvec{\phi }||\partial \widetilde{{\mathcal {L}}}_{{{\widetilde{X}}}}^{I_2}g|, \end{aligned}$$

We can take our first weighted estimate. If $|I_1| \le k-6$, we use (6.42) and (2.28) to get

9.7 $$\begin{aligned}{} & {} {\sum _{\begin{array}{c} \le k \\ {|I_1| \le k-6} \end{array}}}\!\!\!\! \left\Vert {\tau _{+}^{s}}{\tau _{-}^{1/2}}g^{\alpha \beta }D_{\alpha }D_{{{\widetilde{X}}}_1}^{I_1}\phi \nabla _\beta {\mathcal {L}}_{{{\widetilde{X}}}_2}^{I_2}(g^{\gamma \delta }(\widetilde{{\mathcal {L}}}_{Y}g)_{\gamma \delta }) w_{{\overline{\mu }}}^{1/2}\right\Vert _2 \nonumber \\{} & {} \quad \lesssim {\mathcal {E}}_k^{1/2}{\sum _{\le k}}\left\Vert ({\tau _{+}^{s-1}}{\tau _{-}^{-s}}|{{\overline{\partial }}} {\mathcal {L}}_{{{\widetilde{X}}}_2}^{I_2}g| + {\tau _{+}^{-1}}|\partial {\mathcal {L}}_{{{\widetilde{X}}}_2}^{I_2}g|)(\tfrac{w_{{\overline{\mu }}}}{w})^{1/2}\right\Vert _2, \end{aligned}$$

which is bounded by $\varepsilon _g{\mathcal {E}}_k^{1/2}$. If $|I_2| \le k-6$, this can be bounded by

9.8 $$\begin{aligned} \varepsilon _g {\sum _{\le k}}\left\Vert ({\tau _{+}^{s-1+\delta }}{\tau _{-}^{-1/2}}|{\overline{D}}D_{{{\widetilde{X}}}}^{I_1}\varvec{\phi }| + {\tau _{+}^{s-3/2-\gamma /2}}{\tau _{-}^{1/2}}|{D}D_{{{\widetilde{X}}}}^{I_1}\varvec{\phi }|)w_{{\overline{\mu }}}^{1/2}\right\Vert _2 \end{aligned}$$

Then, ${\tau _{+}^{-1+\delta }}<{\tau _{+}^{-1/2-2\delta -{{\overline{\mu }}}}}$, ${\tau _{+}^{s-3/2-\gamma /2}} < {\tau _{+}^{-1/2-2\delta -{{\overline{\mu }}}}}$, so by (4.10a), (4.10c), this is finite, so

9.9 $$\begin{aligned} {\sum _{\le k}}\left\Vert {\tau _{+}^{s}}{\tau _{-}^{1/2}}A_1^jw_{{\overline{\mu }}}^{1/2}\right\Vert _2 \lesssim \varepsilon _g{\mathcal {E}}_k^{1/2} \end{aligned}$$

We consider $A^2_j$. Proposition 2.4 allows us to replace ${{^{({{\widetilde{X}}^I})}{\widetilde{\Pi }}}}$ with $\widetilde{{\mathcal {L}}}_{{\widetilde{X}}}^I H_1$ and reduce to the case $|I_1| > k-6$. We can take our worst $L^\infty$ estimates on the $D\varvec{\phi }$ and $\partial \nabla \cdot Y$ terms. The remaining quantities in the second term are therefore bounded by

9.10 $$\begin{aligned} {\sum _{\le k}}{\mathcal {E}}_k^{1/2}\varepsilon _g\left\Vert {\tau _{+}^{s-2+\delta }}{\tau _{-}^{-1-s}}|\widetilde{{\mathcal {L}}}_{{\widetilde{X}}}^{I_1}H_1|w_{{\overline{\mu }}}^{1/2}\right\Vert _2, \end{aligned}$$

which is easily bounded by ${\mathcal {E}}_k^{1/2}\varepsilon _g$. Therefore,

9.11 $$\begin{aligned} {\sum _{\le k}}\left\Vert {\tau _{+}^{s}}{\tau _{-}^{1/2}}A_2^jw_{{\overline{\mu }}}^{1/2}\right\Vert _2 \lesssim \varepsilon _g{\mathcal {E}}_k^{1/2} \end{aligned}$$

We now look at $A_j^3$. If $k-6$ vector fields or fewer appear in

$$\begin{aligned} ({\mathcal {L}}_{{{\widetilde{X}}}_2}^{I_2}{\textbf{F}})_{{\widetilde{Y}}_1\alpha } D_{{{\widetilde{X}}}_3}^{I_3}\varvec{\phi }\end{aligned}$$

For these terms, we split ${{^{({{{\widetilde{X}}}_1^{I_1}})}{\Pi }}}$ into *g* and ${{^{({{{\widetilde{X}}}_1^{I_1}})}{\widetilde{\Pi }}}}$. and bound the resultant terms the same way as (9.7) and (9.10), respectively.

Otherwise, we use a null decomposition combined with the estimates (8.7) and (2.24).

9.12 $$\begin{aligned} {\sum _{\le k}}\left\Vert {\tau _{+}^{s}}{\tau _{-}^{1/2}}A_3^jw_{{\overline{\mu }}}^{1/2}\right\Vert _2 \lesssim \varepsilon _g{\mathcal {E}}_k^{1/2} \end{aligned}$$

We can state our first subresult:

9.13 $$\begin{aligned} {\sum _{\le k}}\left\Vert {\tau _{+}^{s}}{\tau _{-}^{1/2}}{\mathcal {L}}_{{{\widetilde{X}}}_1}^{I_1}\left( -D^\alpha {D_{{{\widetilde{X}}}_2}^{I_2}}\varvec{\phi }\nabla _\alpha (\nabla \cdot Y)\right) w_{{\overline{\mu }}}^{1/2}\right\Vert _2 \lesssim \varepsilon _g{\mathcal {E}}_k^{1/2} \end{aligned}$$

### Bounding $B_{|I|}$

**The Second Term.** We now look at (9.4b), which we may reduce to bounding

9.14 $$\begin{aligned} {\sum _{\le k}}\left\Vert {\tau _{+}^{s}}{\tau _{-}^{1/2}}{{\mathcal {L}}_{{\widetilde{X}}}^{{\mathbb {C}}{I_1}}}\left( D_\alpha \left( {{^{({{\widetilde{Y}}})}{\widetilde{\Pi }}}}^{\alpha \beta }D_\beta D_{{\widetilde{X}}}^{I_2}\varvec{\phi }\right) \right) w_{{\overline{\mu }}}^{1/2}\right\Vert _2, \end{aligned}$$

as the difference features a scalar multiple of the metric, and thus we may bound it using $A_{|I|-1}, B_{|I|-1}, C_{|I|-1}$. We will commute ${{\mathcal {L}}_{{\widetilde{X}}}^{{\mathbb {C}}{I_1}}}$ through $D_\alpha$, noting the commutator

9.15 $$\begin{aligned}{} & {} \left| [{{\mathcal {L}}_{{\widetilde{X}}}^{{\mathbb {C}}{I_1}}}, D_\alpha ]\left( {{^{({Y})}{\widetilde{\Pi }}}}^{\alpha \beta }D_\beta D_{{\widetilde{X}}}^{I_2}\varvec{\phi }\right) \right| \nonumber \\{} & {} \quad \lesssim \sum _{|I_3| + 1 + |I_4| = |I_1|} \left| {{\mathcal {L}}_{{\widetilde{X}}}^{{\mathbb {C}}{I_3}}}[{{\mathcal {L}}_{{\widetilde{Y}}_1}^{{\mathbb {C}}{ }}}, D_\alpha ]{{\mathcal {L}}_{{\widetilde{X}}}^{{\mathbb {C}}{I_4}}}\left( {{^{({X_2})}{\widetilde{\Pi }}}}^{\alpha \beta }D_\beta D_{{\widetilde{X}}}^{I_2}\varvec{\phi }\right) \right| . \end{aligned}$$

We first bound

$$\begin{aligned} {\sum _{\begin{array}{c} \le k \\ {|I_1|\ge 1} \end{array}}}\left\Vert {\tau _{+}^{s}}{\tau _{-}^{1/2}}D_\alpha \left( {{^{({X^{I_1}})}{\widetilde{\Pi }}}}^{\alpha \beta } {{\mathcal {L}}_{{\widetilde{X}}}^{{\mathbb {C}}{I_2}}}(DD_{{\widetilde{X}}}^{I_3}\varvec{\phi })_\beta \right) w_{{\overline{\mu }}}^{1/2}\right\Vert _2. \end{aligned}$$

We will prove this for $r^* > (t+1)/2$, as the interior case is easier. Taking the null decomposition in $\beta$ and applying the product rule gives

9.16a $$\begin{aligned}&{\sum _{\le k}}\Vert {\tau _{+}^{s}}{\tau _{-}^{1/2}}D_\alpha \left( {{^{({X^{I_1}})}{\widetilde{\Pi }}}}^{\alpha \beta } {{\mathcal {L}}_{{\widetilde{X}}}^{{\mathbb {C}}{I_2}}}(DD_{{\widetilde{X}}}^{I_3}\varvec{\phi })_\beta \right) w_{{\overline{\mu }}}^{1/2}\Vert _2 \nonumber \\&\quad \lesssim {\sum _{\le k}}\Vert {\tau _{+}^{s}}{\tau _{-}^{1/2}}\nabla _\alpha \left( {{^{({{\widetilde{X}}^{I_1}})}{\widetilde{\Pi }}}}^{\alpha {\underline{{\widetilde{L}}}}}\right) {{\mathcal {L}}_{{\widetilde{X}}}^{{\mathbb {C}}{I_2}}}(DD_{{\widetilde{X}}}^{I_3}\varvec{\phi })_{\underline{{\widetilde{L}}}}w_{{\overline{\mu }}}^{1/2}\Vert _2 + \end{aligned}$$

9.16b $$\begin{aligned}&\quad + {\sum _{\le k}}\Vert {\tau _{+}^{s}}{\tau _{-}^{1/2}}\nabla _\alpha \left( {{^{({{\widetilde{X}}^{I_1}})}{\widetilde{\Pi }}}}^{\alpha {{\mathcal {T}}}}\right) {{\mathcal {L}}_{{\widetilde{X}}}^{{\mathbb {C}}{I_2}}}(DD_{{\widetilde{X}}}^{I_3}\varvec{\phi })_{{\mathcal {T}}}w_{{\overline{\mu }}}^{1/2}\Vert _2 + \end{aligned}$$

9.16c $$\begin{aligned}&\quad + {\sum _{\le k}}\Vert {\tau _{+}^{s}}{\tau _{-}^{1/2}}{{^{({{\widetilde{X}}^{I_1}})}{\widetilde{\Pi }}}}^{\alpha {\underline{{\widetilde{L}}}}} D_\alpha \left( {{\mathcal {L}}_{{\widetilde{X}}}^{{\mathbb {C}}{I_2}}}(DD_{{\widetilde{X}}}^{I_3}\varvec{\phi })_{\underline{{\widetilde{L}}}}\right) w_{{\overline{\mu }}}^{1/2}\Vert _2 + \end{aligned}$$

9.16d $$\begin{aligned}&\quad + {\sum _{\le k}}\Vert {\tau _{+}^{s}}{\tau _{-}^{1/2}}{{^{({{\widetilde{X}}^{I_1}})}{\widetilde{\Pi }}}}^{\alpha {{\mathcal {T}}}} D_\alpha \left( {{\mathcal {L}}_{{\widetilde{X}}}^{{\mathbb {C}}{I_2}}}(DD_{{\widetilde{X}}}^{I_3}\varvec{\phi })_{{\mathcal {T}}}\right) w_{{\overline{\mu }}}^{1/2}\Vert _2 \end{aligned}$$

We first bound (9.16a) and (9.16b). First, by (8.1), and recalling (2.36),

9.17a $$\begin{aligned}&{\sum _{\begin{array}{c} \le k \\ {|I_2|+|I_3|\le k-6} \end{array}}}\left\Vert {\tau _{+}^{s}}{\tau _{-}^{1/2}} \nabla _\alpha (\widetilde{{\mathcal {L}}}_{{\widetilde{X}}}^{I_1}H_1)^{\alpha {{\mathcal {T}}}}{{\mathcal {L}}_{{\widetilde{X}}}^{{\mathbb {C}}{I_2}}}(DD_{{\widetilde{X}}}^{I_3}\varvec{\phi })_{{\mathcal {T}}}w_{{\overline{\mu }}}^{1/2}\right\Vert _2 \nonumber \\&\quad \lesssim {\mathcal {E}}_k(T)^{1/2}{\sum _{\le k}}\left\Vert {\tau _{+}^{-1}}{\tau _{-}^{{\overline{\mu }}}}\nabla _\alpha (\widetilde{{\mathcal {L}}}_{{\widetilde{X}}}^{I_1}H_1)^{\alpha {{\mathcal {T}}}}\right\Vert _2, \end{aligned}$$

9.17b $$\begin{aligned}&{\sum _{\begin{array}{c} \le k \\ {|I_2|+|I_3|\le k-6} \end{array}}}\left\Vert {\tau _{+}^{s}}{\tau _{-}^{1/2}} \nabla _\alpha (\widetilde{{\mathcal {L}}}_{{\widetilde{X}}}^{I_1}H_1)^{\alpha {\underline{{\widetilde{L}}}}}{{\mathcal {L}}_{{\widetilde{X}}}^{{\mathbb {C}}{I_2}}}(DD_{{\widetilde{X}}}^{I_3}\varvec{\phi })_{\underline{{\widetilde{L}}}}w_{{\overline{\mu }}}^{1/2}\right\Vert _2 \nonumber \\&\quad \lesssim {\mathcal {E}}_k(T)^{1/2}{\sum _{\le k}}\left\Vert {\tau _{+}^{s-1}}{\tau _{-}^{{\overline{\mu }}-s}}\nabla _\alpha (\widetilde{{\mathcal {L}}}_{{\widetilde{X}}}^{I_1}H_1)^{\alpha {\underline{{\widetilde{L}}}}}\right\Vert _2. \end{aligned}$$

All of these are bounded by $\varepsilon _g{\mathcal {E}}_k(T)^{1/2}$. Next, (2.24) gives

$$\begin{aligned}&\sum _{\begin{array}{c} \le k \\ 1\le |I_1| \le k-6 \end{array}}\left\Vert {\tau _{+}^{s}}{\tau _{-}^{1/2+{{\overline{\mu }}}}} \nabla _\alpha (\widetilde{{\mathcal {L}}}_{{\widetilde{X}}}^{I_1}H_1)^{\alpha {{\mathcal {T}}}}{{\mathcal {L}}_{{\widetilde{X}}}^{{\mathbb {C}}{I_2}}}(DD_{{\widetilde{X}}}^{I_3}\varvec{\phi })_{{\mathcal {T}}}w^{1/2}\right\Vert _2 \\&\quad \lesssim \varepsilon _g{\sum _{\le k}}\left\Vert {\tau _{+}^{s-1+\delta }}{\tau _{-}^{-1/2+{{\overline{\mu }}}}}{{\mathcal {L}}_{{\widetilde{X}}}^{{\mathbb {C}}{I_2}}}(DD_{{\widetilde{X}}}^{I_3}\varvec{\phi })_{{\mathcal {T}}}w^{1/2}\right\Vert _2, \\&\sum _{\begin{array}{c} \le k \\ 1\le |I_1| \le k-6 \end{array}}\left\Vert {\tau _{+}^{s}}{\tau _{-}^{1/2+{{\overline{\mu }}}}} \nabla _\alpha (\widetilde{{\mathcal {L}}}_{{\widetilde{X}}}^{I_1}H_1)^{\alpha {\underline{{\widetilde{L}}}}}{{\mathcal {L}}_{{\widetilde{X}}}^{{\mathbb {C}}{I_2}}}(DD_{{\widetilde{X}}}^{I_3}\varvec{\phi })_{\underline{{\widetilde{L}}}}w^{1/2}\right\Vert _2 \\&\quad \lesssim \varepsilon _g{\sum _{\le k}}\left\Vert {\tau _{0}^{s}}{\tau _{-}^{s-3/2+2{{\overline{\mu }}}}}{{\mathcal {L}}_{{\widetilde{X}}}^{{\mathbb {C}}{I_2}}}(DD_{{\widetilde{X}}}^{I_3}\varvec{\phi })_{\underline{{\widetilde{L}}}}w^{1/2}\right\Vert _2. \end{aligned}$$

Both of these are bounded by $\varepsilon _g{\mathcal {E}}_k(T)^{1/2}$, using (8.7) (including (8.7d)). A similar argument using (2.36) allows us to replace $(\widetilde{{\mathcal {L}}}_{{\widetilde{X}}}^{I_1}H_1)$ with ${{^{({{\widetilde{X}}^{I_1}})}{\widetilde{\Pi }}}}$.

Now we bound (9.16c) and (9.16d). First, we consider the case where the derivative falls on ${\textbf{F}}$. We recall the decompositions

$$\begin{aligned} |{{\widetilde{L}}}(\psi )| + \sum _i|{{\widetilde{S}}_{i}}(\psi )| \lesssim {\tau _{+}^{-1}}\sum _{|I|=1}|{\widetilde{X}}^I\psi |,\quad |{\underline{{\widetilde{L}}}}(\psi )| \lesssim {\tau _{-}^{-1}}\sum _{|I|=1}|{\widetilde{X}}^I\psi |. \end{aligned}$$

We combine this with Lemma 2.1 to get

9.18a $$\begin{aligned} \sum _{|I|\le k-2}|{{\mathcal {U}}}(({\mathcal {L}}_{{\widetilde{X}}}^I {\textbf{F}})_{{\widetilde{X}}{{\mathcal {U}}}})|&\lesssim \sum _{|J|\le k-1}{\tau _{0}^{-1}}|{\mathcal {L}}_{{\widetilde{X}}}^J{\textbf{F}}| \end{aligned}$$

9.18b $$\begin{aligned} \sum _{|I|\le k-2}|{{\mathcal {T}}}(({\mathcal {L}}_{{\widetilde{X}}}^I {\textbf{F}})_{{\widetilde{X}}{{\mathcal {U}}}})|&\lesssim \sum _{|J|\le k-1}|{\mathcal {L}}_{{\widetilde{X}}}^J{\textbf{F}}| \end{aligned}$$

9.18c $$\begin{aligned} \sum _{|I|\le k-2}{{\mathcal {U}}}(({\mathcal {L}}_{{\widetilde{X}}}^I {\textbf{F}})_{{\widetilde{X}}{{\mathcal {T}}}})&\lesssim \sum _{|J|\le k-1}|{\mathcal {L}}_{{\widetilde{X}}}^J{\textbf{F}}| + {\tau _{0}^{-1}}(|\alpha [{\mathcal {L}}_{{\widetilde{X}}}^J{\textbf{F}}]| + |\rho [{\mathcal {L}}_{{\widetilde{X}}}^J{\textbf{F}}]|+|\sigma [{\mathcal {L}}_{{\widetilde{X}}}^J{\textbf{F}}]|) \end{aligned}$$

When the derivative falls on derivatives of $\varvec{\phi }$, we use the estimates

9.19a $$\begin{aligned} \sum _{|I|\le k-1}|D_{{{\widetilde{L}}}} D_{{\mathcal {T}}}D_{{\widetilde{X}}}^I\varvec{\phi }|&\lesssim \sum _{|J|\le k}\left| {\tau _{+}^{-1}}D_{{{\widetilde{L}}}} D_{{\widetilde{X}}}^J\varvec{\phi }\right| + \left| {\tau _{+}^{-2}}D_{{\widetilde{X}}}^J\varvec{\phi }\right| \end{aligned}$$

9.19b $$\begin{aligned} \sum _{|I|\le k-1}|D_{{{\widetilde{S}}_{j}}}D_{{\mathcal {T}}}D_{{\widetilde{X}}}^I\varvec{\phi }|&\lesssim \sum _{|J|\le k}\left| {\tau _{+}^{-1}}D_{{{\widetilde{S}}_{j}}}D_{{\widetilde{X}}}^J\varvec{\phi }\right| + \left| {\tau _{+}^{-2}}D_{{\widetilde{X}}}^J{\varvec{\phi }}\right| \end{aligned}$$

9.19c $$\begin{aligned} \sum _{|I|\le k-1}|D_{{\underline{{\widetilde{L}}}}}D_{{\mathcal {T}}}D_{{\widetilde{X}}}^I\varvec{\phi }|&\lesssim \sum _{|J|\le k}\left| {\tau _{+}^{-1}}D_{{\underline{{\widetilde{L}}}}}D_{{\widetilde{X}}}^J\varvec{\phi }\right| + \left| {\tau _{+}^{-2}}D_{{\widetilde{X}}}^J\varvec{\phi }\right| \end{aligned}$$

9.19d $$\begin{aligned} \sum _{|I|\le k-1}|D_{{\underline{{\widetilde{L}}}}}D_{{\underline{{\widetilde{L}}}}}D_{{\widetilde{X}}}^I\varvec{\phi }|&\lesssim \sum _{|J|\le k}\left| {\tau _{-}^{-1}}D_{{\underline{{\widetilde{L}}}}}D_{{\widetilde{X}}}^J\varvec{\phi }\right| \end{aligned}$$

For all other derivatives we use the identity

$$\begin{aligned} D_{U}D_{{\underline{{\widetilde{L}}}}}\psi = D_{{\underline{{\widetilde{L}}}}}D_{U}\psi + D_{[U, {\underline{{\widetilde{L}}}}]}\psi + i{\textbf{F}}_{U{\underline{{\widetilde{L}}}}}\psi . \end{aligned}$$

For $U\in {{\mathcal {T}}}$, $[U, {\underline{{\widetilde{L}}}}]$ is either 0 or $\frac{1}{r^*}{{\widetilde{S}}_{j}}$. Combining these gives

9.20a $$\begin{aligned} \sum _{\begin{array}{c} |I|\le k-1 \\ T\in {{\mathcal {T}}} \end{array}}|D_TD_{\underline{{\widetilde{L}}}}D_{{\widetilde{X}}}^I\varvec{\phi }|\lesssim & {} \sum _{|J|\le k}{\tau _{+}^{-1}}|DD_{{\widetilde{X}}}^J\varvec{\phi }|\nonumber \\{} & {} +{\tau _{+}^{-2}}|D_{{\widetilde{X}}}^J\varvec{\phi }| + \sum _{|I|\le k-1 }|{\textbf{F}}||D_{{\widetilde{X}}}^{I}\varvec{\phi }|. \end{aligned}$$

We now put everything together. Again, we may replace ${{^{({{\widetilde{X}}^J})}{\widetilde{\Pi }}}}$ with $\widetilde{{\mathcal {L}}}_{{\widetilde{X}}}^J H$ and bound the difference using the estimates (2.36). In the cases where we can bound $|\widetilde{{\mathcal {L}}}_{{\widetilde{X}}}^J H|$ in $L^\infty$ our estimates follow from (2.24) and (8.7) (with the worse weights of ). Otherwise the bounds (8.1) and (6.42) imply

9.21 $$\begin{aligned}&{\sum _{\begin{array}{c} \le k \\ {|I_2|+|I_3|\le k-6} \end{array}}}\left\Vert {\tau _{+}^{s}}{\tau _{-}^{1/2}}\left( (\widetilde{{\mathcal {L}}}_{{\widetilde{X}}}^IH_1)^{\alpha U} D_\alpha {{\mathcal {L}}_{{\widetilde{X}}}^{{\mathbb {C}}{I_2}}}(DD_{{\widetilde{X}}}^{I_3}\phi )_U\right) w_{{\overline{\mu }}}^{1/2}\right\Vert _2 \nonumber \\&\quad \lesssim \sum _{|I|\le k}\left\Vert {\tau _{+}^{s-2}}{\tau _{-}^{-s}}|(\widetilde{{\mathcal {L}}}_{{\widetilde{X}}}^IH_1)|\right\Vert _2 + \sum _{|I|\le k}\left\Vert {\tau _{+}^{s-1}}{\tau _{-}^{-s-1}}|(\widetilde{{\mathcal {L}}}_{{\widetilde{X}}}^IH_1)^{{\underline{{\widetilde{L}}}}{\underline{{\widetilde{L}}}}}|\right\Vert _2. \end{aligned}$$

These can be bounded by (2.25a) and (2.25b) without issue. Therefore,

9.22 $$\begin{aligned} {\sum _{\begin{array}{c} \le k \\ {|I_1|\ge 1} \end{array}}}\left\Vert {\tau _{+}^{s}}{\tau _{-}^{1/2}}D_\alpha \left( {{^{({X^{I_1}})}{\widetilde{\Pi }}}}^{\alpha \beta } {{\mathcal {L}}_{{\widetilde{X}}}^{{\mathbb {C}}{I_2}}}(DD_{{\widetilde{X}}}^{I_3}\varvec{\phi })_\beta \right) w_{{\overline{\mu }}}^{1/2}\right\Vert _2 \lesssim \varepsilon _g{{\mathcal {E}}}_k^{1/2}. \end{aligned}$$

Finally, we bound the right-hand side of (9.15). We have four types of terms we need to bound here:

9.23a $$\begin{aligned}&\sum _{|I_3| + 1 + |I_4| = |I_1|} \Big \Vert {\tau _{+}^{s}}{\tau _{-}^{1/2}}{{\mathcal {L}}_{{\widetilde{X}}}^{{\mathbb {C}}{I_3}}}[{{\mathcal {L}}_{{\widetilde{Y}}_1}^{{\mathbb {C}}{ }}}, D_\alpha ]{{\mathcal {L}}_{{\widetilde{X}}}^{{\mathbb {C}}{I_4}}}\Big ({{^{({{{\widetilde{X}}}_2})}{\widetilde{\Pi }}}}^{\alpha \beta }D_\beta D_{{\widetilde{X}}}^{I_2}\varvec{\phi }\Big )w_{{\overline{\mu }}}^{1/2}\Big \Vert _2 \nonumber \\&\quad \lesssim \big \Vert {\tau _{+}^{s}}{\tau _{-}^{1/2}}({\mathcal {L}}_{{{\widetilde{X}}}}^{I_1}{\textbf{F}})_{{\widetilde{Y}}_1\alpha }({\mathcal {L}}_{{{\widetilde{X}}}}^{I_2}{\textbf{F}})_{{\widetilde{Y}}_2\beta }{{^{({{{\widetilde{X}}}^{I_3}})}{\widetilde{\Pi }}}}^{\alpha \beta }D_{{{\widetilde{X}}}}^{I_4}\varvec{\phi }w_{{{\overline{\mu }}}}^{1/2}\big \Vert _2 \end{aligned}$$

9.23b $$\begin{aligned}&\quad +\big \Vert {\tau _{+}^{s}}{\tau _{-}^{1/2}}({\mathcal {L}}_{{{\widetilde{X}}}}^{I_1}{\textbf{F}})_{{\widetilde{Y}}_1\alpha }{{^{({{{\widetilde{X}}}^{I_3}})}{\widetilde{\Pi }}}}^{\alpha \beta }D_\beta D_{{{\widetilde{X}}}}^{I_4}\varvec{\phi }w_{{{\overline{\mu }}}}^{1/2}\big \Vert _2 \end{aligned}$$

9.23c $$\begin{aligned}&\quad +\big \Vert {\tau _{+}^{s}}{\tau _{-}^{1/2}}\nabla _\alpha ({{\widetilde{X}}}^I(\nabla \cdot {\widetilde{Y}}) )({\mathcal {L}}_{{{\widetilde{X}}}}^{I_2}{\textbf{F}})_{{\widetilde{Y}}_2\beta }{{^{({{{\widetilde{X}}}^{I_3}})}{\widetilde{\Pi }}}}^{\alpha \beta }D_{{{\widetilde{X}}}}^{I_4}\varvec{\phi }w_{{{\overline{\mu }}}}^{1/2}\big \Vert _2 \end{aligned}$$

9.23d $$\begin{aligned}&\quad +\big \Vert {\tau _{+}^{s}}{\tau _{-}^{1/2}}\nabla _\alpha ({{\widetilde{X}}}^I(\nabla \cdot {\widetilde{Y}})){{^{({{{\widetilde{X}}}^{I_3}})}{\widetilde{\Pi }}}}^{\alpha \beta }D_\beta D_{{{\widetilde{X}}}}^{I_4}\varvec{\phi }w_{{{\overline{\mu }}}}^{1/2}\big \Vert _2 \end{aligned}$$

We first deal with (9.23a). We prove this with ${{^{({{\widetilde{X}}^{I_3}})}{\widetilde{\Pi }}}}$ replaced by $\widetilde{{\mathcal {L}}}_{{\widetilde{X}}^{I_3}}$ and use (2.36) to bound the remainder. Additionally, we decompose ${\textbf{F}}= {\textbf{F}}^0 + {\textbf{F}}^1$. Then, if ${\textbf{F}}^0$ appears twice and if $|I_3| \le k-6$, a null decomposition and (2.24) give

$$\begin{aligned}{} & {} \sum _{\begin{array}{c} \le k \\ |I_3|\le k-6 \end{array}}\big \Vert {\tau _{+}^{s}}{\tau _{-}^{1/2}}({\mathcal {L}}_{{{\widetilde{X}}}}^{I_1}{\textbf{F}})_{{\widetilde{Y}}_1\alpha }({\mathcal {L}}_{{{\widetilde{X}}}}^{I_2}{\textbf{F}})_{{\widetilde{Y}}_2\beta }{{^{({{{\widetilde{X}}}^{I_3}})}{\widetilde{\Pi }}}}^{\alpha \beta }D_{{{\widetilde{X}}}}^{I_4}\varvec{\phi }w_{{{\overline{\mu }}}}^{1/2}\big \Vert _2 \\{} & {} \quad \lesssim {\sum _{\le k}}\varepsilon _g|q|^2\Vert {\tau _{+}^{s-1}}{\tau _{0}^{\gamma -\delta }}{\tau _{-}^{-3/2+\delta }}D_{{\widetilde{X}}}^{I_4}w^{1/2}\Vert _2\lesssim \varepsilon _g{{\mathcal {E}}}_k^{3/2}. \end{aligned}$$

Otherwise, the bound is similar to (9.21). For all other terms, we apply Lemma 6.3, noting $|I_4|\le k-2$. First, for $I_1, I_2 \le k-6$ the bound ${{\overline{\mu }}}< \tfrac{1}{2} - s$ along with $L^\infty$ estimates gives

9.24 $$\begin{aligned}{} & {} \big \Vert {\tau _{+}^{s}}{\tau _{-}^{1/2}}({\mathcal {L}}_{{{\widetilde{X}}}}^{I_1}{\textbf{F}})_{{\widetilde{Y}}_1\alpha }({\mathcal {L}}_{{{\widetilde{X}}}}^{I_2}{\textbf{F}})_{{\widetilde{Y}}_2\beta }{{^{({{{\widetilde{X}}}^{I_3}})}{\widetilde{\Pi }}}}^{\alpha \beta }D_{{{\widetilde{X}}}}^{I_4}\varvec{\phi }w_{{{\overline{\mu }}}}^{1/2}\big \Vert _2\nonumber \\{} & {} \quad \lesssim |q|^2(\Vert {\tau _{+}^{s-1}}{\tau _{-}^{-1-s}}|\widetilde{{\mathcal {L}}}_{{\widetilde{X}}}^{I_3}H_1|^{{\underline{{\widetilde{L}}}}{\underline{{\widetilde{L}}}}}\Vert _2 + \Vert {\tau _{+}^{-1}}{\tau _{-}^{-1}}|\widetilde{{\mathcal {L}}}_{{\widetilde{X}}}^{I_3}H_1|\Vert _2). \end{aligned}$$

We may bound these using (2.25b) and (2.25c). Otherwise, we take a null decomposition and use our $L^\infty$ bounds to reduce this to an $L^2$ norm on ${\textbf{F}}^1$.

Bounds on the other terms are much simpler. We can bound (9.23b) in the same way as (9.16c) and (9.16d). In order to bound (9.23c) and (9.23d), we apply (9.12) and (9.11), respectively.

Therefore,

9.25 $$\begin{aligned} \sum _{|I_3| + 1 + |I_4| = |I_1|} \Big \Vert {\tau _{+}^{s}}{\tau _{-}^{1/2}}{{\mathcal {L}}_{{\widetilde{X}}}^{{\mathbb {C}}{I_3}}}[{{\mathcal {L}}_{{\widetilde{Y}}_1}^{{\mathbb {C}}{ }}}, D_\alpha ]{{\mathcal {L}}_{{\widetilde{X}}}^{{\mathbb {C}}{I_4}}}\Big ({{^{({{{\widetilde{X}}}_2})}{\widetilde{\Pi }}}}^{\alpha \beta }D_\beta D_{{\widetilde{X}}}^{I_2}\varvec{\phi }\Big )w_{{\overline{\mu }}}^{1/2}\Big \Vert _2\lesssim \varepsilon _g{{\mathcal {E}}}_k^{1/2}.\nonumber \\ \end{aligned}$$

### Bounding $C_{|I|}$

**The Final Term:** We now attempt to bound (9.4c). The primary concern here is that, even in the Minkowski spacetime, we have terms like $({\mathcal {L}}_{X_1}^{I_1}{\textbf{F}})_{Y{\underline{{\widetilde{L}}}}}D_{{\widetilde{L}}}D_{X_2}^{I_2}\varvec{\phi }$. This does not decay well, so we must utilize additional cancellation noted in [26]. The commutator terms take the form:

9.26 $$\begin{aligned} {\sum _{\le k}}-i {{\mathcal {L}}_{{\widetilde{X}}}^{{\mathbb {C}}{I_1}}}\left( \nabla _\beta (g^{\alpha \beta }( {\textbf{F}}_{{\widetilde{Y}}_1\alpha })) D_{{\widetilde{X}}}^{I_2}\varvec{\phi }+ 2{\textbf{F}}_{{\widetilde{Y}}_1\alpha }g^{\alpha \beta }D_\beta ( D_{{\widetilde{X}}}^{I_2}\varvec{\phi })\right) . \end{aligned}$$

We can bound these terms using the pointwise estimate

9.27 $$\begin{aligned}&{\sum _{\le k}}\left| {{\mathcal {L}}_{{\widetilde{X}}}^{{\mathbb {C}}{I_1}}}\left( \nabla _\beta (g^{\alpha \beta }( {\textbf{F}}_{{\widetilde{Y}}_1\alpha })) D_{{\widetilde{X}}}^{I_2}\varvec{\phi }+ 2{\textbf{F}}_{{\widetilde{Y}}_1\alpha }D_\beta (g^{\alpha \beta } D_{{\widetilde{X}}}^{I_2}\varvec{\phi })\right) \right| \nonumber \\&\quad \lesssim {\sum _{\le k}}\left| [{\mathcal {L}}_{{\widetilde{X}}}^{I_1}, \nabla _\beta ](g^{\alpha \beta }{\textbf{F}}_{{\widetilde{Y}}_1\alpha })D_{{\widetilde{X}}}^{I_2}\varvec{\phi }\right| + {\sum _{\le k}}\left| ({\mathcal {L}}_{{\widetilde{X}}}^{I_1}(i_{{\widetilde{Y}}_1} {\textbf{F}}))^\beta [{{\mathcal {L}}_{{\widetilde{X}}}^{{\mathbb {C}}{I_2}}}, D_\beta ]D_{{\widetilde{X}}}^{I_3}\varvec{\phi }\right| \nonumber \\&\quad +{\sum _{\le k}}\left| \nabla _\beta \left( {\mathcal {L}}_{{\widetilde{X}}}^{I_1}(g^{-1}i_{{\widetilde{Y}}_1}{\textbf{F}})\right) ^\beta D_{{\widetilde{X}}}^{I_2}\varvec{\phi }+ 2({\mathcal {L}}_{{\widetilde{X}}}^{I_1}(g^{-1}i_{{\widetilde{Y}}_1}{\textbf{F}}))^\beta D_\beta D_{{\widetilde{X}}}^{I_2}\varvec{\phi }\right| , \nonumber \\&\quad \lesssim C^1_{k} + C^2_k + C^3_k. \end{aligned}$$

We look at the three terms on the right. The identity (2.43f) allows us to bound $C_k^1$ using (9.12).

To bound $C_k^2$ we first use (2.43a) and (9.23a) to reduce the problem to bounding

9.28 $$\begin{aligned} {\sum _{\le k}}\big \Vert {\tau _{+}^{s}}{\tau _{-}^{1/2}}g^{\alpha \beta }{\mathcal {L}}_{{\widetilde{X}}}^{I_1}{\textbf{F}}_{{\widetilde{Y}}_1\alpha }{\mathcal {L}}_{{\widetilde{X}}}^{I_2}{\textbf{F}}_{{\widetilde{Y}}_2\beta }D_{{\widetilde{X}}}^{I_3}\varvec{\phi }w_{{\overline{\mu }}}^{1/2}\big \Vert _2. \end{aligned}$$

As usual, we split ${\textbf{F}}= {\textbf{F}}^0 + {\textbf{F}}^1$. A null decomposition gives the bounds

9.29a $$\begin{aligned} \big |{\tau _{+}^{s}}{\tau _{-}^{1/2}}(g-{{\widehat{m}}})^{\alpha \beta }{\mathcal {L}}_{{\widetilde{X}}}^{I_1}{\textbf{F}}^0_{{\widetilde{Y}}_1\alpha }{\mathcal {L}}_{{\widetilde{X}}}^{I_2}{\textbf{F}}^0_{{\widetilde{Y}}_2\beta }\big |&\lesssim \varepsilon _g|q|^2{\tau _{+}^{s-1}}{\tau _{0}^{\gamma -\delta }}{\tau _{-}^{-3/2+\delta }}, \end{aligned}$$

9.29b $$\begin{aligned} \big |{\tau _{+}^{s}}{\tau _{-}^{1/2}}{{\widehat{m}}}^{\alpha \beta }{\mathcal {L}}_{{\widetilde{X}}}^{I_1}{\textbf{F}}^0_{{\widetilde{Y}}_1\alpha }{\mathcal {L}}_{{\widetilde{X}}}^{I_2}{\textbf{F}}^0_{{\widetilde{Y}}_2\beta }\big |&\lesssim \varepsilon _g|q|^2{\tau _{+}^{s-1}}{\tau _{0}^{}}{\tau _{-}^{-1/2}}. \end{aligned}$$

In the support of ${\textbf{F}}^0$, we have the bound ${\tau _{-}^{-1/2}}w_{{\overline{\mu }}}\lesssim w'$, which, combined with the inequality and (7.1) gives

9.30 $$\begin{aligned} {\sum _{\le k}}\big \Vert {\tau _{+}^{s}}{\tau _{-}^{1/2}}g^{\alpha \beta }{\mathcal {L}}_{{\widetilde{X}}}^{I_1}{\textbf{F}}^0_{{\widetilde{Y}}_1\alpha }{\mathcal {L}}_{{\widetilde{X}}}^{I_2}{\textbf{F}}^0_{{\widetilde{Y}}_2\beta }D_{{\widetilde{X}}}^{I_3}\varvec{\phi }w_{{\overline{\mu }}}^{1/2}\big \Vert _2\lesssim \varepsilon _g{{\mathcal {E}}}_k^{1/2}. \end{aligned}$$

For terms containing ${\textbf{F}}^1$, we note that $|I_1|+|I_2|+|I_3|\le k-2$. It therefore suffices to bound

9.31a $$\begin{aligned}&{\sum _{\le k}}\big \Vert {\tau _{+}^{s-1}}{\tau _{-}^{1-s}}g^{\alpha \beta }|{\mathcal {L}}_{{\widetilde{X}}}^{I_1}{\textbf{F}}^1_{{\widetilde{Y}}_1\alpha }||{\mathcal {L}}_{{\widetilde{X}}}^{I_2}{\textbf{F}}^0_{{\widetilde{Y}}_2\beta }|\big \Vert _2, \end{aligned}$$

9.31b $$\begin{aligned}&{\sum _{\le k}}\big \Vert {\tau _{+}^{s-1}}{\tau _{-}^{1-s+{{\overline{\mu }}}}}g^{\alpha \beta }|{\mathcal {L}}_{{\widetilde{X}}}^{I_1}{\textbf{F}}^1_{{\widetilde{Y}}_1\alpha }||{\mathcal {L}}_{{\widetilde{X}}}^{I_2}{\textbf{F}}^1_{{\widetilde{Y}}_2\beta }|\langle \big \Vert _2. \end{aligned}$$

For each of these, we take a null decomposition and (6.4). To bound (9.31a) we apply the bounds (2.24) and bound the ${\textbf{F}}$ terms using (2.50). In order to bound (9.31b) we follow the same process, bounding $|{\mathcal {L}}_{{\widetilde{X}}}^{I_1}{\textbf{F}}^1_{{\widetilde{Y}}_1{\underline{{\widetilde{L}}}}}|$ and $|{\mathcal {L}}_{{\widetilde{X}}}^{I_2}{\textbf{F}}^1_{{\widetilde{Y}}_2{\underline{{\widetilde{L}}}}}|$ in $L^\infty$ whenever one of them appears. Combining these results gives

9.32 $$\begin{aligned} \Vert {\tau _{+}^{s}}{\tau _{-}^{1/2}}C_k^2 w_{{\overline{\mu }}}^{1/2}\Vert _2 \lesssim {{\mathcal {E}}}_k^{3/2}. \end{aligned}$$

We focus on $C_k^3$, which we may reduce to the region $r^*>(t+1)/2$. We first write

9.33 $$\begin{aligned} D_\alpha D_{{\widetilde{X}}}^{I_3}\varvec{\phi }= \tfrac{1}{r^*}D_\alpha (r^*D_{{\widetilde{X}}}^{I_3}\varvec{\phi }) - \tfrac{\partial _\alpha (r^*)}{r^*}D_{{\widetilde{X}}}^{I_3}\varvec{\phi }. \end{aligned}$$

Therefore, we can write

9.34 $$\begin{aligned} C^3_k= & {} \left( \nabla _\alpha \left( {\mathcal {L}}_{{{\widetilde{X}}}}^{I_1}(g^{-1}i_{{{\widetilde{Y}}}_1}{\textbf{F}})\right) ^\alpha \ - 2\tfrac{\partial _\alpha (r^*)}{r^*}\left( {\mathcal {L}}_{{{\widetilde{X}}}}^{I_1}(g^{-1}i_{{{\widetilde{Y}}}_1}{\textbf{F}})\right) ^\alpha \right) D_{{\widetilde{X}}}^{I_2}\varvec{\phi }\nonumber \\{} & {} + 2\left( {\mathcal {L}}_{{{\widetilde{X}}}}^{I_1}(g^{-1}i_{{{\widetilde{Y}}}_1}{\textbf{F}})\right) ^\alpha \Big (\tfrac{D_\alpha (r^*D_{{\widetilde{X}}}^{I_2}\varvec{\phi })}{r^*}\Big ). \end{aligned}$$

We first bound

9.35 $$\begin{aligned} \left\Vert {\tau _{+}^{s}}{\tau _{-}^{1/2}}\left( {\mathcal {L}}_{{\widetilde{X}}}^{I_1}(g^{-1}i_{Y_1}{\textbf{F}})\right) ^\alpha \tfrac{D_\alpha \left( r^*D_{{\widetilde{X}}}^{I_2}\varvec{\phi }\right) }{r^*}w_{{\overline{\mu }}}^{1/2}\right\Vert _2. \end{aligned}$$

When the derivative falls on $g^{-1}$ we expand using $\widetilde{{\mathcal {L}}}_{{\widetilde{X}}}^{I}$. Terms with the modified deformation tensor ${{^{({{\widetilde{X}}^{I_1}})}{\widetilde{\Pi }}}}$ may be bounded in a similar fashion to (9.23a), so it remains to bound

$$\begin{aligned} \left\Vert {\tau _{+}^{s}}{\tau _{-}^{1/2}}g^{\alpha \beta }({\mathcal {L}}_{{\widetilde{X}}}^{I_1}{\textbf{F}})_{{\widetilde{Y}}_1\alpha } \tfrac{D_\beta (r^*D_{{\widetilde{X}}}^{I_2}\varvec{\phi })}{r^*}w_{{\overline{\mu }}}^{1/2}\right\Vert _2 \end{aligned}$$

We may replace $g^{\alpha \beta }$ with ${{\widehat{m}}}^{\alpha \beta }$, noting that the norm corresponding to the difference may be bounded in the same way as (9.23a). Likewise, the terms containing ${\textbf{F}}^0$ are easily bounded. We bound the remainder by expanding $({\mathcal {L}}_{{\widetilde{X}}}^{I_1}{\textbf{F}}^1)_{{\widetilde{Y}}_1\alpha }$ in its null decomposition, and using the $L^2(L^\infty )$ bounds (5.17) and (6.42) to bound $\alpha$ and $D_{\underline{{\widetilde{L}}}}\psi$ terms whenever applicable, bounding the remainder using $E_k$ energy norms. For all other components, we use the $L^\infty (L^\infty )$ bounds (5.17), (6.42), and (2.50), and pair them with $S_k$ energy norms. Consequently,

9.36 $$\begin{aligned} \left\Vert {\tau _{+}^{s}}{\tau _{-}^{1/2}}\left( {\mathcal {L}}_{{\widetilde{X}}}^{I_1}(g^{-1}i_{Y_1}{\textbf{F}})\right) ^\alpha \tfrac{D_\alpha \left( r^*D_{{\widetilde{X}}}^{I_2}\varvec{\phi }\right) }{r^*}w_{{\overline{\mu }}}^{1/2}\right\Vert _2 \lesssim {\mathcal {E}}_k(T). \end{aligned}$$

We now seek to bound

9.37 $$\begin{aligned}{} & {} \Big \Vert {\tau _{+}^{s}}{\tau _{-}^{1/2}}\Big (\nabla _\alpha \left( {\mathcal {L}}_{{{\widetilde{X}}}}^{I_1}(g^{-1}i_{{{\widetilde{Y}}}_1}{\textbf{F}})\right) ^\alpha \ \nonumber \\{} & {} \quad - 2\tfrac{\partial _\alpha (r^*)}{r^*}\left( {\mathcal {L}}_{{{\widetilde{X}}}}^{I_1}(g^{-1}i_{{{\widetilde{Y}}}_1}{\textbf{F}})\right) ^\alpha \Big )D_{{\widetilde{X}}}^{I_2}\varvec{\phi }w_{{\overline{\mu }}}^{1/2}\Big \Vert _2 \end{aligned}$$

Again, when the Lie derivative falls on the metric, we decompose using the reduced Lie derivative $\widetilde{{\mathcal {L}}}_{}$. A null decomposition similar to the one used to bound (9.16), combined with the inequality $r^{*-1}\lesssim {\tau _{+}^{-1}}$ in our region of concern, gives

9.38 $$\begin{aligned}{} & {} {\sum _{\le k}}\Big \Vert {\tau _{+}^{s}}{\tau _{-}^{1/2}}\nabla _\alpha \Big ({{^{({{\widetilde{X}}^{I_1}})}{\widetilde{\Pi }}}}^{\alpha \beta }({\mathcal {L}}_{{{\widetilde{X}}}}^{I_2}{\textbf{F}})_\beta \Big )\nonumber \\{} & {} \quad - 2\tfrac{\partial _\alpha (r^*)}{r^*}({{^{({{\widetilde{X}}^{I_1}})}{\widetilde{\Pi }}}}^{\alpha \beta })({\mathcal {L}}_{{{\widetilde{X}}}}^{I_2}{\textbf{F}})_\beta D_{{\widetilde{X}}}^{I_2}\varvec{\phi }w_{{\overline{\mu }}}^{1/2}\Big \Vert _2 \lesssim {\mathcal {E}}(T) \end{aligned}$$

To deal with the remainder, it suffices to bound

9.39 $$\begin{aligned} {\sum _{\le k}}\left\Vert {\tau _{+}^{s}}{\tau _{-}^{1/2}}g^{\alpha \beta }\left( \nabla _\alpha ({\mathcal {L}}_{{\widetilde{X}}}^{I_1}{\textbf{F}})_{{\widetilde{Y}}_1\beta }) - \tfrac{2\partial _\alpha (r^*)}{r^*}({\mathcal {L}}_{{\widetilde{X}}}^{I_1}{\textbf{F}})_{{\widetilde{Y}}_1\beta })\right) D_{{\widetilde{X}}}^{I_2}\varvec{\phi }w_{{\overline{\mu }}}^{1/2}\right\Vert _2\nonumber \\ \end{aligned}$$

We decompose

9.40 $$\begin{aligned} \nabla ^\alpha ({\mathcal {L}}_{{\widetilde{X}}}^{I_1}{\textbf{F}})_{{\widetilde{Y}}_1\alpha } = {\textbf{J}}[{\mathcal {L}}_{{\widetilde{X}}}^{I_1}{\textbf{F}}]_{{\widetilde{Y}}_1}D_{{\widetilde{X}}}^{I_2}\varvec{\phi }+ (\nabla ^\alpha {\widetilde{Y}}_1^\beta )({\mathcal {L}}_{{\widetilde{X}}}^{I_1}{\textbf{F}})_{\beta \alpha }D_{{\widetilde{X}}}^{I_2}\varvec{\phi }. \end{aligned}$$

If at least one Lie derivative falls on ${\textbf{F}}$ in the first term, we may bound

9.41 $$\begin{aligned}{} & {} {\sum _{\begin{array}{c} \le k \\ {|I_1|\le k-2} \end{array}}}\left\Vert {\tau _{+}^{s}}{\tau _{-}^{1/2}}J[{\mathcal {L}}_{{\widetilde{X}}}^{I_1}{\textbf{F}}]_{{\widetilde{Y}}_1}D_{{\widetilde{X}}}^{I_2}\varvec{\phi }w_{{\overline{\mu }}}^{1/2}\right\Vert _2 \nonumber \\{} & {} \quad \lesssim {\mathcal {E}}_k^{1/2}{\sum _{\le k}}\left\Vert {\tau _{+}^{s-1}}{\tau _{-}^{1-s}}{\textbf{J}}[{\mathcal {L}}_{{\widetilde{X}}}^{I_1}{\textbf{F}}]_{{\widetilde{Y}}_1} w_{{\overline{\mu }}}^{1/2}\right\Vert _2. \end{aligned}$$

Decomposing $Y_1$ in terms of null vectors and using the relation $1-s < 1/2$ allows us to bound this quantity by

$$\begin{aligned} \left\Vert {\textbf{J}}[{\mathcal {L}}_{{\widetilde{X}}}^{I_1}{\textbf{F}}]\right\Vert _{L^2[w]}. \end{aligned}$$

In particular, we commute the Lie derivative through, bound the estimate as in (8.13), and take the null decomposition. If no Lie derivatives fall on ${\textbf{F}}$, we bound the corresponding norm straightforwardly using the $L^\infty$ norm

$$\begin{aligned} |{\textbf{J}}_{{\widetilde{Y}}_1}| \lesssim {\mathcal {E}}_k{\tau _{+}^{-1-s}}{\tau _{-}^{-s}} \end{aligned}$$

Finally, we consider

$$\begin{aligned} \left( (\nabla ^\alpha {\widetilde{Y}}_1^\beta ) - \tfrac{2\nabla ^\alpha (r^*)}{r^*}{\widetilde{Y}}_1^\beta \right) ({\mathcal {L}}_{X_1}^{I_1}{\textbf{F}})_{\beta \alpha }D_{X_2}^{I_2}\varvec{\phi }. \end{aligned}$$

We lower the indices and consider the null decomposition. To bound the corresponding term in (9.39) it suffices to show the bound

$$\begin{aligned} \Big ((\nabla _\alpha Y_{1\beta }) - \tfrac{2\nabla _\alpha (r^*)}{r^*}Y_{1\beta }\Big )\widetilde{U}_1^\alpha \widetilde{U}_2^\beta \lesssim 1, \end{aligned}$$

for $\widetilde{U}_1, \widetilde{U}_2 \in {{\mathcal {U}}}$ and apply our usual $L^2(L^2)$ and $L^\infty (L^\infty )$ estimates. However, to bound ${\textbf{F}}^{{\underline{{\widetilde{L}}}}{{\widetilde{S}}_{j}}}$ we instead need

9.42 $$\begin{aligned} \Big (\nabla _\alpha {\widetilde{Y}}_{1\beta }-\nabla _\beta {\widetilde{Y}}_{1\alpha } - \tfrac{2\nabla _\alpha (r^*)}{r^*}{\widetilde{Y}}_{1\beta }\Big ){{\widetilde{L}}}^\alpha {{{\widetilde{S}}_{i}}}{}^\beta \lesssim {\tau _{0}^{1-\delta }}. \end{aligned}$$

We may replace $\nabla _\beta , \nabla _\alpha$ with $\partial _\beta , \partial _\alpha$, as a null decomposition combined with the bounds (2.24) implies

$$\begin{aligned} |\Gamma _{{{\widetilde{L}}}{{\widetilde{S}}_{i}}{\widetilde{Y}}_1}| + |\Gamma _{{{\widetilde{S}}_{i}}{{\widetilde{L}}}{\widetilde{Y}}_1}|\lesssim {\tau _{+}^{-1}}. \end{aligned}$$

Expanding in the null decomposition and using the bound (2.24) lets us bound all terms by the right-hand side of (9.42). The remainder follows from direct calculation. Therefore,

9.43 $$\begin{aligned} \left( \nabla _\alpha {\widetilde{Y}}_{1\beta }-\nabla _\beta {\widetilde{Y}}_{1\alpha }- \tfrac{2\partial _\alpha (r^*)}{r^*}{\widetilde{Y}}_{1\beta }\right) {\textbf{F}}^{\alpha \beta } \lesssim {\tau _{+}^{-1+\delta }}|{\underline{\alpha }}| + |\rho | + |\sigma | + |\alpha |. \end{aligned}$$

Our estimate follows. We can combine everything:

#### Theorem 9.2

If $({\textbf{F}}, \varvec{\phi })$ solves (1.14), and

$$\begin{aligned} {\mathcal {E}}_k(T)\le 1, \end{aligned}$$

then

9.44 $$\begin{aligned} {\sum _{\le k}}\left\Vert \left( \Box ^{{\mathbb {C}}}_g{\mathcal {L}}_{{\widetilde{X}}}^I\varvec{\phi }\right) {\tau _{+}^{s}}{\tau _{-}^{1/2}}w_{{\overline{\mu }}}^{1/2}\right\Vert _2 \lesssim ({\mathcal {E}}_k^{1/2}+\varepsilon _g){\mathcal {E}}_k^{1/2}. \end{aligned}$$

## Appendix: Inequalities

We start by stating Kato’s diamagnetic inequality, which will be useful in the estimates to follow. Given a complex scalar field $\phi$ and a vector field *Z*, we have the inequality

10.1 $$\begin{aligned} \left| Z(|\phi |)\right| \le |D_Z\phi |. \end{aligned}$$

This follows from the pointwise Cauchy–Schwarz inequality applied to the identity

$$\begin{aligned} Z(|\phi |) = {\mathfrak {R}}\left( \langle \phi /|\phi |, D_Z\phi \rangle \right) , \end{aligned}$$

which follows almost directly from (1.9). Consequently, in the Sobolev-type estimates to follow, we can replace all cases of $Z(\phi )$ with $D_Z(\phi )$.

### Lemma 10.1

For any $q > 2$, and for any function $\phi$ with sufficient regularity, we have the following inequality on the sphere ${\mathbb {S}}^2_r$ of radius *r*, as long as $r > t/2$, $r > 1/2$:

10.2 $$\begin{aligned} \sup _{{\mathbb {S}}^2_r}|\chi \phi | \lesssim _q {\tau _{+}^{-2/q}}\left( \sum _{|I| \le 1, Z \in {\mathbb {O}}}\left\Vert Z(\chi \phi ) \right\Vert _{L^q(S_r^2)}\right) . \end{aligned}$$

### Proof

This is a straightforward consequence of Morrey’s inequality applied to two charts on the unit sphere, and scaling to the sphere of radius *r* (and introducing a factor of $r^{-2/q}$). The presence of the cutoff $\chi$ allows us to use the estimate $r^{-2/q}\lesssim {\tau _{+}^{-2/q}}$. $\square$

### Lemma 10.2

For $2 \le q < 4$, if $r > t/4$ and $t >1$, we have

10.3 $$\begin{aligned}{} & {} \left\Vert \chi \phi \right\Vert _{L^\infty _r L^q({{\mathbb {S}}^2_r})} \lesssim _q {\tau _{+}^{-1+2/q}}{\tau _{-}^{-1/2}}\nonumber \\{} & {} \quad \left( \left\Vert {\tau _{-}^{}}{\partial _{r^*}}(\chi \phi )\right\Vert _{L^2_x} + \sum _{|I|\le 1, Z \in {\mathbb {O}}}\left\Vert Z(\chi \phi )\right\Vert _{L^2_x}\right) . \end{aligned}$$

### Proof

This follows from the Sobolev estimate on a cylinder, rescaled to a dyadic region. We first define the dyadic decomposition $\{{\mathcal {I}}_i^{\pm }\}$ for a given time slice $\Sigma _t$ as follows:

10.4 $$\begin{aligned} {\mathcal {U}}_i = \left\{ x: r^* > t/2, 2^i \le |{u^{*{}}}| + 1 \le 2^{i+1}\right\} . \end{aligned}$$

We subdivide these as follows:

10.5 $$\begin{aligned} {\mathcal {U}}^+_i= & {} \left\{ x: r^*> t/2, 2^i \le |{u^{*{}}}| +1 \le 2^{i+1}, {u^{*{}}}> 0\right\} , \quad \nonumber \\ {\mathcal {U}}^-_i= & {} \left\{ x: r^* > t/2, 2^i \le |{u^{*{}}}| + 1 \le 2^{i+1}, {u^{*{}}}< 0\right\} . \end{aligned}$$

Thus, ${\mathcal {U}}^+$ are supported in the interior, and ${\mathcal {U}}^-$ are supported in the exterior. Additionally, for any given time slice, ${\mathcal {U}}_i^+$ is empty for sufficiently large *i*. We can construct a partition of unity $\{\chi _{{\mathcal {U}}_i^\pm }\}$ such that the support of each is in the region $\{ x: 2^{i-1} \le |{u^{*{}}}| \le 2^{i+2}, r^* > t/4\}$ and derivatives satisfy the bound ${\partial _{r^*}}(\chi _{{\mathcal {U}}_i^\pm }) \lesssim 2^{-i}$ for some constant independent of *i*.

We now define the cylindrical region

10.6 $$\begin{aligned} ({\widetilde{r}}, \omega ) \in {\mathcal {A}} = [1/4, 4] \times {\mathbb {S}}^2. \end{aligned}$$

We take maps from our cylinder to the region ${\mathcal {U}}_i^\pm$ as follows:

10.7 $$\begin{aligned} ({\widetilde{r}}, \omega ) \rightarrow (t, t\pm 2^i{\widetilde{r}}\omega ), \end{aligned}$$

with an appropriate cutoff (recall that *t* is fixed). Here we scale the radial variable by approximately ${\tau _{-}^{}}$ and the spherical variables by ${\tau _{+}^{}}$. Then we take the fractional Sobolev estimates on the region ${\mathcal {A}}$

10.8a $$\begin{aligned} \left\Vert \chi \phi \right\Vert _{L^\infty ({\mathbb {R}})}&\lesssim \left\Vert \chi \phi \right\Vert _{H^{1/2 + 2\epsilon _1}}, \end{aligned}$$

10.8b $$\begin{aligned} \left\Vert \chi \phi \right\Vert _{L^q({\mathbb {R}}^2)}&\lesssim \left\Vert \chi \phi \right\Vert _{H^{1 - 2/q + 2\epsilon _2}}, \end{aligned}$$

which hold for all $\epsilon _i > 0, 2 \le q < 4$. Since the inequality

10.9 $$\begin{aligned} (1+|\xi _x|^2)^{1/4+\epsilon _1}(1+|\xi _y|^2)^{1/2-1/q+\epsilon _2} \lesssim (1+|\xi _x|^2 + |\xi _y|^2)^{1/2} \end{aligned}$$

holds in the phase space for sufficiently small $\epsilon _i$ (depending on *q*), taking charts gives us the inclusion inequality

10.10 $$\begin{aligned} \left\Vert \chi \phi \right\Vert _{L^\infty ({\mathbb {R}}_{\ge 0})L^q({\mathbb {S}}^2)} \lesssim \left\Vert \chi \phi \right\Vert _{H^1({\mathcal {A}})}. \end{aligned}$$

We can take our change of variables, noting scaling, to get the estimate (10.3).

$$\square $$

This covers our estimates for the extended exterior. We now look at the far interior.

### Lemma 10.3

If $t \ge 1$, $r < 3/4t$, we have the following estimates for compactly supported functions *f*:

10.11a $$\begin{aligned} \left\Vert f\right\Vert _{L^\infty _x}&\lesssim t^{-1/2}\sum _{\begin{array}{c} X \in \{{{\widetilde{S}}}, {{\widetilde{\Omega }}_{{0i}}}\} \\ |I| \le 1 \end{array}}\left\Vert X^I f \right\Vert _{L^6_x} \end{aligned}$$

10.11b $$\begin{aligned} \left\Vert f\right\Vert _{L^6_x}&\lesssim t^{-1}\sum _{\begin{array}{c} X \in \{{{\widetilde{S}}}, {{\widetilde{\Omega }}_{{0i}}}\} \\ |I| \le 1 \end{array}}\left\Vert X^I f \right\Vert _{L^2_x} \end{aligned}$$

### Proof

This follows from fixing time and rescaling to the unit ball, noting that for fixed time *t*

$$\begin{aligned} t\left\Vert \nabla f\right\Vert _{L^p(B_r)} \lesssim \sum _{\begin{array}{c} X \in \{{{\widetilde{S}}}, {{\widetilde{\Omega }}_{{0i}}}\} \\ |I| \le 1 \end{array}}\left\Vert X^I(f)\right\Vert _{L^p(B_r)}. \end{aligned}$$

This follows almost identically from the proof in [18], noting that $|\partial \phi |$ and $|{\widetilde{\partial }}\phi |$ are equivalent. $\square$

Finally, we consider the light cone estimate. As in [18], this is not strictly necessary in closing our estimate, as we can get our full results using an $L^2(t)L^\infty (x)$ estimate following from the time slice Sobolev estimates. However, this estimate gives us more precise control over the asymptotic behavior:

### Lemma 10.4

For $2 \le q < 4$, we have the global estimate

10.12 $$\begin{aligned} \left\Vert \chi \phi \right\Vert _{L^\infty _{v^*}L^q({\mathbb {S}}^2_{{\underline{u}^{*{}}}})} \lesssim {\tau _{+}^{-3/2-2/q}}\sum _{\begin{array}{c} X \in \{{\underline{u}^{*{}}}{{\widetilde{L}}}, {\mathbb {O}}\} \\ |I| \le 1 \end{array}}\left\Vert X^I(\chi \phi )\right\Vert _{L^2(C({u^{*{}}}))}, \end{aligned}$$

where we have parametrized the cone by $({\underline{u}^{*{}}}, \omega )$, and ${\mathbb {S}}^2_{v^*}$ is the subset of $C({u^{*{}}})$ with constant ${\underline{u}^{*{}}}$.

### Proof

This is similar to the proof of inequality (10.3), with two differences. First, due to boundary considerations along the light cone, we need to take a Sobolev extension function across the endpoints of the time slab $t \in [1, T]$. Second, we take our dyadic decomposition in ${\underline{u}^{*{}}}$ instead of ${u^{*{}}}$. This introduces a factor of ${\tau _{+}^{}}$ instead of ${\tau _{-}^{}}$ in the analogue to the radial derivative $\partial _{{\tilde{r}}}$ in the cylinder. However, this is paired with ${{\widetilde{L}}}$, a nicer behaving directional derivative.

$$\square $$

We can now put everything together:

### Theorem 10.5

Given a smooth test function $\phi$, we have the following estimates:

10.13 $$\begin{aligned} \left\Vert {\tau _{+}^{1+\delta _+}}{\tau _{-}^{1/2+\delta _-}}\chi \phi w^{1/2}\right\Vert _{L^\infty _x}&\lesssim \sum _{\begin{array}{c} |I|, |J| \le 1 \\ X \in \{{\tau _{-}^{}}{\partial _{r^*}}\} \cup {\mathbb {O}}, Y \in {\mathbb {O}} \end{array}}\left\Vert {\tau _{+}^{\delta _+}}{\tau _{-}^{\delta _-}}X^I Y^J (\chi \phi )w^{1/2}\right\Vert _{L^2_x} \end{aligned}$$

10.14 $$\begin{aligned} \left\Vert {\tau _{+}^{3/2+\delta _+}}\chi \phi w^{1/2}\right\Vert _{L^\infty (C_{{u^{*{}}}})}&\lesssim \sum _{\begin{array}{c} |I|, |J| \le 1\\ X \in \{{\underline{u}^{*{}}}{{\widetilde{L}}}, {\mathbb {O}}\}, Y \in {\mathbb {O}} \end{array}} \left\Vert {\tau _{+}^{\delta _+}}X^I Y^J(\chi \phi )w^{1/2}\right\Vert _{L^2(C_{{u^{*{}}}})} \end{aligned}$$

10.15 $$\begin{aligned} \left\Vert {\tau _{+}^{3/2+\delta _+}}(1-\chi )\phi w^{1/2}\right\Vert _{L^\infty _x}&\lesssim \sum _{\begin{array}{c} |J| \le 2 \\ Z \in \{{{\widetilde{S}}}, {{\widetilde{\Omega }}_{{0i}}}\} \end{array}}\left\Vert {\tau _{+}^{\delta _+}}Z^I((1-\chi )\phi )w^{1/2}\right\Vert _{L^2_x} \end{aligned}$$

as well as their complex covariant equivalents

10.16 $$\begin{aligned} \left\Vert {\tau _{+}^{1+\delta _+}}{\tau _{-}^{1/2+\delta _-}}\chi \phi \right\Vert _{L^\infty ({\mathbb {R}}^3)}&\lesssim \sum _{\begin{array}{c} |I|, |J| \le 1 \\ X \in \{{\tau _{-}^{}}{\partial _{r^*}}\} \cup {\mathbb {O}}, Y \in {\mathbb {O}} \end{array}}\left\Vert {\tau _{+}^{\delta _+}}{\tau _{-}^{\delta _-}} D_X^I D_Y^J (\chi \phi )\right\Vert _{L^2({\mathbb {R}}^3)}, \end{aligned}$$

10.17 $$\begin{aligned} \left\Vert {\tau _{+}^{3/2+\delta _+}}\chi \phi \right\Vert _{L^\infty (C_{{u^{*{}}}})}&\lesssim \sum _{\begin{array}{c} |I|, |J| \le 1\\ X \in \{{\underline{u}^{*{}}}{{\widetilde{L}}}, {\mathbb {O}}\}, Y \in {\mathbb {O}} \end{array}} \left\Vert {\tau _{+}^{\delta _+}}D_X^I D_Y^J(\chi \phi )\right\Vert _{L^2(C_{{u^{*{}}}})}, \end{aligned}$$

10.18 $$\begin{aligned} \left\Vert {\tau _{+}^{3/2+\delta _+}}(1-\chi )\phi \right\Vert _{L^\infty ({\mathbb {R}}^3)}&\lesssim \sum _{\begin{array}{c} |J| \le 2 \\ Z \in \{{{\widetilde{S}}}, {{\widetilde{\Omega }}_{{0i}}}\} \end{array}}\left\Vert {\tau _{+}^{\delta _+}}D_Z^I((1-\chi )\phi )\right\Vert _{L^2({\mathbb {R}}^3)}. \end{aligned}$$

### Proof

This straightforwardly follows from (10.2)–(10.12), with powers of *w* and $\delta _\pm$ added during the dyadic decomposition. $\square$

We now prove a model weighted Hardy inequality. The proof is adapted from [10] and is readily adaptable to other weighted Hardy estimates.

### Lemma 10.6

For any function $\phi \in C_0^\infty$, we have the inequality

10.19 $$\begin{aligned} {\int _{\Sigma _{t}}} (t+r)^2\left| \tfrac{\psi }{r}\right| ^2\, dx \lesssim {\int _{\Sigma _{t}}}(r-t)^2\left| \tfrac{\partial _r(r\psi )}{r}\right| ^2 \, dx. \end{aligned}$$

### Proof

By transforming into spherical coordinates and restricting to constant $\omega$, we reduce this to proving the one-dimensional inequality

10.20 $$\begin{aligned} \int _0^\infty \left( \tfrac{t+r}{r}\right) ^2(r\psi )^2 \, dr \lesssim \int _0^\infty (r-t)^2\partial _r(r\psi )^2 \, dr. \end{aligned}$$

The proof is as follows: given generic weight functions *f*, *g*, and an arbitrary constant *C*, we have the inequality

10.21 $$\begin{aligned} \int _0^\infty \left( Cf\partial _r\Psi + g\Psi \right) ^2 - \partial _r(Cfg\Psi ^2)\, dr \ge 0, \end{aligned}$$

which holds when $fg\Psi ^2$ is absolutely continuous and vanishes at 0 and at $\infty$. This is satisfied for $\Psi = r\psi$, where $\psi$ is compactly supported. We can think of *f* and *g* as weight functions, and *C* is an arbitrary constant. We can rewrite this as

10.22 $$\begin{aligned} \int _0^\infty \left( C\partial _r(fg) - g^2\right) (r\psi )^2 \lesssim \int _0^\infty C^2f^2(\partial _r(r\psi ))^2 \, dr. \end{aligned}$$

In general, we will choose *f*, *g*, *C* such that, for some $c > 0$, $(C\partial _r(fg) - g^2) > c g^2$. We select

$$\begin{aligned} f = r-t, \quad g = \tfrac{r+t}{r}. \end{aligned}$$

Then,

$$\begin{aligned} \partial _r(fg) = \tfrac{r^2+t^2}{r^2}. \end{aligned}$$

Selecting $C = 4$ we see that

$$\begin{aligned} \left( C\partial _r(fg) - g^2\right) \ge \left( \tfrac{r+t}{r}\right) ^2. \end{aligned}$$

The inequality (10.20) follows. $\square$

### Lemma 10.7

For $\tfrac{1}{2} < s \le 1$, and for compactly supported *f*, we have the estimate

10.23 $$\begin{aligned} {\int _{\Sigma _{t}}} {\tau _{+}^{2s}} \left| \tfrac{\psi }{r}\right| ^2 w \, dx&\lesssim {\int _{\Sigma _{t}}} {\tau _{-}^{2s}}\left| \tfrac{D_{r^*}(r^*\psi )}{r}\right| ^2 w \, dx. \end{aligned}$$

### Proof

Due to (10.1), it suffices to prove this if $D_{r^*}$ is replaced with ${\partial _{r^*}}$. As in the previous lemma, we reduce to the one-dimensional inequality

10.24 $$\begin{aligned} \int _0^\infty {\tau _{+}^{2s}}|\psi |^2 w \, dr \lesssim \int _0^\infty {\tau _{-}^{2s}}|{\partial _{r^*}}(r^*\psi )|^2 w \, dr. \end{aligned}$$

Since *dr* and $dr^*$ are equivalent, we can replace the former with the latter without issue. We now take inequality (10.22), with $r^*$ in place of *r*, and

10.25a $$\begin{aligned} f&= |r^*-t|^s\text {sgn}(r^*-t)w^{1/2}, \end{aligned}$$

10.25b $$\begin{aligned} g&= \tfrac{|r^*+t|^s}{r^*}w^{1/2}. \end{aligned}$$

We have that in this case is equal to

$$\begin{aligned} \partial _r^*(fg) = \text {sgn}(r^*-t)\tfrac{2r^{*2}s|r^{*2}-t^2|^{s-1}\text {sgn}(r^*-t) - |r^{*2}-t^2|^s}{r^{*2}}w + \tfrac{{\partial _{r^*}}(w)}{w}(fg). \end{aligned}$$

The last term is strictly positive, as ${\partial _{r^*}}(w)$ is supported when $r^* - t > 0$. We can rewrite

$$\begin{aligned} \partial _r^*(fg) \ge \tfrac{((2s-1)r^{*2} + t^2)|r^{*2}-t^2|^{s-1}}{r^{*2}}w. \end{aligned}$$

Choose *C* such that $(2\,s-1)C \ge 4$. Then, noting $s-1 \le 0$, it follows that

$$\begin{aligned} C\partial _r^*(fg) \ge \tfrac{4|r^{*2}+t^2|^{s}}{r^{*2}}w. \end{aligned}$$

For $s \le 1$, we have

$$\begin{aligned} C\partial _r^*(fg) - g^2 \ge g^2. \end{aligned}$$

This gives us the preliminary estimate

10.26 $$\begin{aligned} {\int _{\Sigma _{t}}}|r^*+t|^{2s}\left| \tfrac{\psi }{r}\right| ^2 w \, dx \lesssim {\int _{\Sigma _{t}}}|r^*-t|^{2s}\left| \tfrac{D_{r^*}(r^*\psi )}{r}\right| w\,dx. \end{aligned}$$

We can add a time-shifted estimate replacing *t* with $t+1$ to get the full estimate. $\square$

Similar reasoning gives us the inequality

10.27 $$\begin{aligned} {\int _{\Sigma _{t}}}{\tau _{+}^{2s}}{\tau _{0}^{1+2\delta }}\left| \tfrac{\psi }{r^*}\right| ^2(w')\,dx \lesssim {\int _{\Sigma _{t}}} {\tau _{-}^{2s}}{\tau _{0}^{1+2\delta }}\left| \tfrac{D_{r^*}(r^*\psi )}{r^*}\right| ^2(w')\, dx, \end{aligned}$$

as long as we have the inequality $s + \delta < 1$.

We now prove an estimate along the same lines which is better suited to our conformal Morawetz estimate. This is an alternate proof to a similar result in [18]

### Lemma 10.8

For *p*, *q* such that $p > -1$, $|q| < p+1$, and for test functions $\phi$, we have the inequality

10.28 $$\begin{aligned} {\int _{\Sigma _{t}}}{\tau _{-}^{p}}{\tau _{+}^{q}}|\psi |^2 w\, dx \lesssim {\int _{\Sigma _{t}}}{\tau _{-}^{p+2}}{\tau _{+}^{q}}\left| \tfrac{D_{r^*}(r^*\psi )}{r^*}\right| ^2 w\,dx. \end{aligned}$$

### Proof

This follows from the one-dimensional inequality

10.29 $$\begin{aligned} \int _0^\infty {\tau _{-}^{p}}{\tau _{+}^{q}}|r^*\psi |^2 w\, dr^* \lesssim \int _0^\infty {\tau _{-}^{p+2}}{\tau _{+}^{q}}|{\partial _{r^*}}(r^*\psi )|^2 w\, dr^*. \end{aligned}$$

Additionally, we can replace ${\tau _{-}^{}}$ and ${\tau _{+}^{}}$ with $1+|r^*-t|$ and $1+r^*+t$, respectively. We again seek to apply (10.22), with

$$\begin{aligned} f&= (1+|r^*-t|)^{p/2 + 1}{\text {sgn}}(r^*-t)(1+r^*+t)^{q/2}w^{1/2} \\ g&= (1+|r^*-t|)^{p/2}(1+r^*+t)^{q/2}w^{1/2} \end{aligned}$$

Then,

$$\begin{aligned} \partial _{r^*}(fg) = {\left\{ \begin{array}{ll} \left( \tfrac{p+1+2\delta }{1+|r^*-t|} + \tfrac{q}{1+r^*+t}\right) fg &{} r^* > t, \\ \left( \tfrac{p+1}{1+|r^*-t|} - \tfrac{q}{1+r^*+t}\right) fg &{} r^* < t. \end{array}\right. } \end{aligned}$$

Since $g^2 = fg(1+|r^*-t|)^{-1}$ and $1+|r^*-t| < 1+r^*+t$, it follows that

$$\begin{aligned} \partial _{r^*}(fg) \ge (p+1-|q|)g^2. \end{aligned}$$

The result follows. $\square$

## References

[1] Andersson, L., Blue, P.: Uniform energy bound and asymptotics for the Maxwell field on a slowly rotating Kerr black hole exterior. J. Hyperbolic Differ. Equ. **12**(4), 689–743 (2015) 10.1142/S0219891615500204

[2] Bieri, L., Miao, S., Shahshahani, S.: Asymptotic properties of solutions of the Maxwell Klein Gordon equation with small data. Commun. Anal. Geom. **25**(1), 25–96 (2017) 10.4310/CAG.2017.v25.n1.a2

[3] Blue, P., Sterbenz, J.: Uniform decay of local energy and the semi-Schwarzschild space. Commun. Math. Phys. **268**(2), 481–504 (2006) 10.1007/s00220-006-0101-6

[4] Candy, T., Kauffman, C., Lindblad, H.: Asymptotic Behavior of the Maxwell–Klein–Gordon System. In: Communications in Mathematical Physics (2019). ISSN: 1432-0916. 10.1007/s00220-019-03285-y.

[5] Christodoulou, D., Klainerman, S.: The Global Nonlinear Stability of the Minkowski Space. Princeton Mathematical Series, vol. 41. Princeton University Press, Princeton, NJ (1993)

[6] Dafermos, M., Rodnianski, I.: A new physical-space approach to decay for the wave equation with applications to black hole spacetimes. In: 16th International Congress on Mathematical Physics, pp. 421–432. World Sci. Publ. Hackensack (2010)

[7] Eardley, D.M., Moncrief, V.: The global existence of Yang–Mills–Higgs fields in 4-dimensional Minkowski space. Commun. Math. Phys. **83**(2), 171–212 (1982) 10.1007/BF01976040

[8] Fang, A., Wang, Q., Yang, S.: Global solution for Massive Maxwell–Klein–Gordon equations with large Maxwell field. Ann. PDE **7**(3), 1–69 (2021)

[9] He, L.: Scattering from infinity of the Maxwell Klein Gordon Equations. Commun. Math. Phys. **386**(3), 1 (2021) 10.1007/s00220-021-04105-y

[10] Hörmander, L.: Lectures on Nonlinear Hyperbolic Differential Equations. Vol. 26. Mathématiques & Applications (Berlin) [Mathematics & Applications]. Springer, Berlin (1997)

[11] Kauffman, C., Lindblad, H.: Global stability of Minkowski space for the Einstein–Maxwell–Klein–Gordon system in generalized wave coordinates (2021). arXiv:2109.03270

[12] Klainerman, S., Machedon, M.: Finite Energy Solutions of the Yang-Mills Equations in R3+1. Ann. Math. **142**(1), 39–119 (1995) 10.2307/2118611

[13] Klainerman, S., Machedon, M.: On the Maxwell-Klein-Gordon equation with finite energy. Duke Math. J. **74**(1), 19–44 (1994) 10.1215/S0012-7094-94-07402-4

[14] Klainerman, S., Wang, Q., Yang, S.: Global solution for massive Maxwell-Klein-Gordon equations. arXiv e-prints arXiv:1801.10380 (2018)

[15] Lindblad, H., Schlue, V.: Scattering from infinity for semilinear models of Einstein’s equations satisfying the weak null condition. ArXiv e-prints (2017). arXiv:1711.00822 [math.AP]

[16] Lindblad, H.: On the asymptotic behavior of solutions to the Einstein vacuum equations in wave coordinates. Commun. Math. Phys. **353**(1), 135–184 (2017) 10.1007/s00220-017-2876-z

[17] Lindblad, H., Rodnianski, I.: The global stability of Minkowski space-time in harmonic gauge. Ann. of Math. (2) **171**(3), 1401–1477 (2010) 10.4007/annals.2010.171.1401

[18] Lindblad, H., Sterbenz, J.: Global stability for charged-scalar fields on Minkowski space. IMRP Int. Math. Res. Pap. **2006**, 52976 (2006)

[19] Lindblad, H., Tohaneanu, M.: Global existence for quasilinear wave equations close to Schwarzschild. Commun. Part. Differ. Equ. **43**, 893 (2016) 10.1080/03605302.2018.1476529

[20] Loizelet, J.: Problèmes globaux en relativité générale. Universitè Francois Rabelais, Tours, France. PhD thesis (2008)

[21] Oh, S.-J., Tataru, D.: Local well-posedness of the (4 + 1)-dimensional Maxwell–Klein–Gordon equation at energy regularity. Ann. PDE **2**(2), 1 (2016)

[22] Oliver, J.: A vector field method for non-trapping, radiating spacetimes. J. Hyperbolic Differ. Equ. **13**(4), 735–790 (2016) 10.1142/S021989161650020X

[23] Oliver, J., Sterbenz, J.: A vector field method for radiating black hole spacetimes. Anal. PDE **13**, 1 (2017)

[24] Psarelli, M.: Asymptotic behavior of the solutions of Maxwell-Klein-Gordon field equations in 4-dimensional Minkowski space. Commun. Part. Differ. Equ. **24**(1–2), 223–272 (1999) 10.1080/03605309908821421

[25] Shu, W.-T.: Asymptotic properties of the solutions of linear and nonlinear spin field equations in Minkowski space. Commun. Math. Phys. **140**(3), 449–480 (1991) 10.1007/BF02099131

[26] Shu, W.-T.: Global existence of Maxwell-Higgs fields. In: Nonlinear hyperbolic equations and field theory (Lake Como, 1990), Vol. 253, pp. 214–227. Pitman Res. Notes Math. Ser. Longman Sci. Tech., Harlow (1992)

[27] Speck, J.: The nonlinear stability of the trivial solution to the Maxwell–Born–Infeld system. J. Math. Phys. **53**(8), 1 (2012) 10.1063/1.4740047

[28] Sterbenz, J., Tataru, D.: Local energy decay for Maxwell fields Part I: Spherically symmetric black-hole backgrounds. Int. Math. Res. Not. IMRN **11**, 3298–3342 (2015)

[29] Wei, D., Yang, S., Yu, P.: On the global dynamics of Yang–Mills–Higgs equations (2022)

[30] Yang, S.: Decay of solutions of Maxwell-Klein-Gordon equations with large Maxwell field. In: ArXiv e-prints (2015). arXiv: 1511.00251 [math.AP]

[31] Yang, S.: On global behavior of solutions of the Maxwell–Klein–Gordon equations. Adv. Math. **326**, 1 (2015)

[32] Yang, S., Pin, Yu.: On global dynamics of the Maxwell–Klein–Gordon equations. Cambridge J. Math. **7**(4), 365–467 (2019) 10.4310/CJM.2019.v7.n4.a1

[33] Zipser, N.: The global nonlinear stability of the trivial solution of the Einstein–Maxwell equations. Harvard University, Cambridge, Massachusetts. PhD thesis (2000)

